# Rhodium(III)-Catalyzed Redox-Neutral [3+3] Annulation of *N*-nitrosoanilines with Cyclopropenones: A Traceless Approach to Quinolin-4(1*H*)-One Scaffolds

**DOI:** 10.3390/molecules25020268

**Published:** 2020-01-09

**Authors:** Lingjun Liu, Jiyuan Li, Wenhao Dai, Feng Gao, Kaixian Chen, Yu Zhou, Hong Liu

**Affiliations:** 1 State Key Laboratory of Natural Medicines and Department of Medicinal Chemistry, China Pharmaceutical University, 24 Tong Jia Xiang, Nanjing 210009, China; engrossment_llj@163.com (L.L.); dwh1992@163.com (W.D.); 2 State Key Laboratory of Drug Research and Key Laboratory of Receptor Research, Shanghai Institute of Materia Medica, Chinese Academy of Sciences, 555 Zu Chong Zhi Road, Shanghai 201203, China; lijiyuan@shu.edu.cn (J.L.); zjsygao@163.com (F.G.); 3 Open Studio for Druggability Research of Marine Natural Products, Pilot National Laboratory for Marine Science and Technology (Qingdao), 1 Wenhai Road, Aoshanwei, Jimo, Qingdao 266237, China

**Keywords:** Rhodium(III), redox-neutral, [3+3] annulation, *N*-nitrosoaniline, cyclopropenones, quinolin-4(1*H*)-ones

## Abstract

A traceless approach to quinolin-4(1*H*)-one scaffolds through Rh(III)-catalyzed redox-neutral [3+3] cyclization of *N*-nitrosoanilines with cyclopropenones has been achieved. This protocol features short reaction time and atom-economical combination without extra additives, which can be further applied in the construction of privileged heterocyclic compounds in pharmaceutical chemistry.

## 1. Introduction

**Figure 1:**
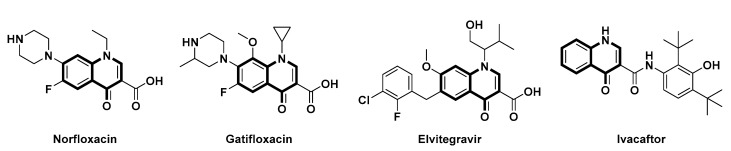
Representative drugs containing quinolin-4(1*H*)-ones.

Quinolin-4(1*H*)-ones are ubiquitously present in numerous natural products and drugs, representing an important class of privileged structures in medicinal chemistry [1,2,3,4,5], such as antibiotics norfloxacin and gatifloxacin, a HIV integrase inhibitor, elvitegravir, and a modulator of ATP-binding cassette transporters, lvacaftor (Figure 1). Therefore, the development of highly efficient protocols both in transition-metal-catalyzed C-H activation and photocatalytic methods for the construction of such *N*-heterocyclic scaffolds is an extremely hot issue in modern organic chemistry [6,7,8,9,10,11,12]. However, the existing methodologies usually require elaborate-to-access starting materials, multiple steps, or harsh reaction conditions, failing to implement a wide range of applications. Given the importance of quinolin-4(1*H*)-ones with broad biological activities, there still remains the need to develop efficient, step- and atom-economic synthetic strategies.

**Scheme 1:**
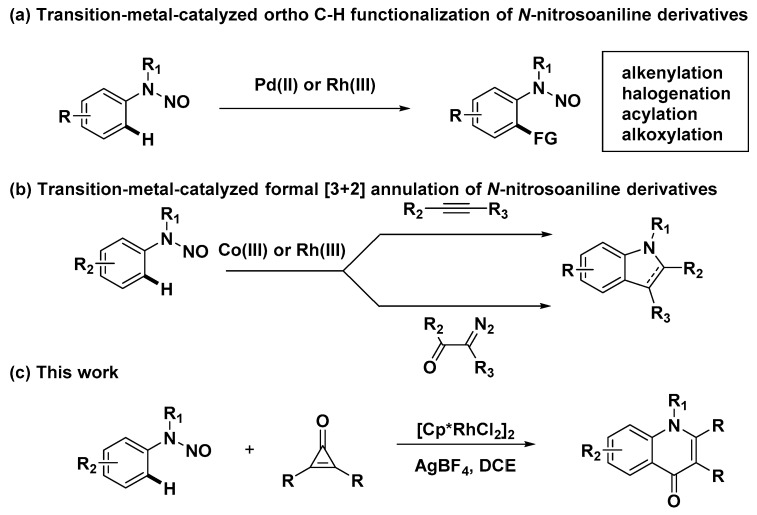
Transition-metal-catalyzed C-H functionalization of *N*-nitrosoanilines.

In the past decade, transition-metal-catalyzed redox-neutral C-H activation reactions have emerged as a robust and versatile methodology, avoiding stoichiometric amounts of external oxidants [13,14,15]. Recently, *N*-nitroso [16,17,18] as a novel directing group has aroused increasing attention and has been successfully employed in transition-metal (e.g., Pd, Rh, etc.) catalyzed C-H functionalization (Scheme 1a) [19,20,21,22]. In 2013, Zhu’s group reported the pioneering work of Rh(III)-catalyzed redox-neutral [3+2] annulation of *N*-nitrosoanilines with internal alkynes to form efficiently indole derivatives (Scheme 1b) [23,24,25,26,27,28]. Similarly, several formal [3+2] annulations between *N*-nitrosoanilines and diazo compounds [29] as well as propargyl alcohols [30] utilizing the *N*−nitroso group as an internal oxidant have been reported to prepare diversified indole scaffolds, in which the substrate involving the *N*−nitroso group seems to be an excellent synthon to build these intriguing privileged structures via a C-H bond activation and further annulation cascade. Therefore, in continuation of our recent efforts on transition-metal-catalyzed C-H annulations for the construction of heterocyclic scaffolds [31,32,33,34,35,36], we surprisingly found a new redox-neutral [3+3] annulation of *N*-nitrosoanilines with cyclopropenones [37,38,39] to generate a different substituted quinolin-4(1*H*)-one scaffold (Scheme 1c), which is a desirable privileged structure for further drug discovery. However, coincidentally, a similar work was reported by Cheng [40] after our work was finished and ready to submit. Compared with Cheng’s strategy, this method without extra additives also enables the efficient preparation of quinolin-4(1*H*)-ones in a much shorter time (2 h vs. 12 h), and has a good substrate scope. 

## 2. Results and Discussions

We initiated our studies by examining the reaction conditions of the coupling of *N*-nitrosoaniline, **1a**, with diphenylcyclopropenone, **2a**, in the presence of a Rh(III) catalyst. As shown in Table 1, three Rh(III) catalysts were firstly explored in dichloroethane (DCE), and the desired product, **3a**, could only be afforded in 13% yield under the presence of [Cp*RhCl_2_]_2_, whereas the other two Rh(III) catalysts or Rh(III)-free were not effective (Table 1, entries 1–4). The structure of **3a** was also unambiguously confirmed by an X-ray crystallographic analysis (see the Supplementary Material for details). However, further explorations demonstrated that a large amount of side product of dimerization of cyclopropenone [41,42] was generated simultaneously in this transformation, which resulted in a low yield of the desired product. Based on these results, we wondered whether lowering the concentration of cyclopropenone could inhibit the formation of the dimerization side product. To our delight, when the concentration was reduced from 0.1 M to 0.02 M, the yield of the desired product was increased dramatically, increasing the yield of **3a** to 72% (entries 5,6). Inspired by the results, we further screened the silver salts and the results revealed that AgBF_4_ was still the most effective, while no desired product was formed in the absence of the silver additive (entries 7–9). Further explorations for reaction solvents displayed that DCE was the best choice for this transformation (entries 10,11). In addition, we attempted some complex additives with HOAc, CsF, or Zn(OAc)_2_, respectively, but they led to a slightly decreasing yield (entries 12–14). Similarly, reducing the reaction temperature to 80 °C or 60 °C was also detrimental to this transformation (entries 15,16).

**Scheme 2:**
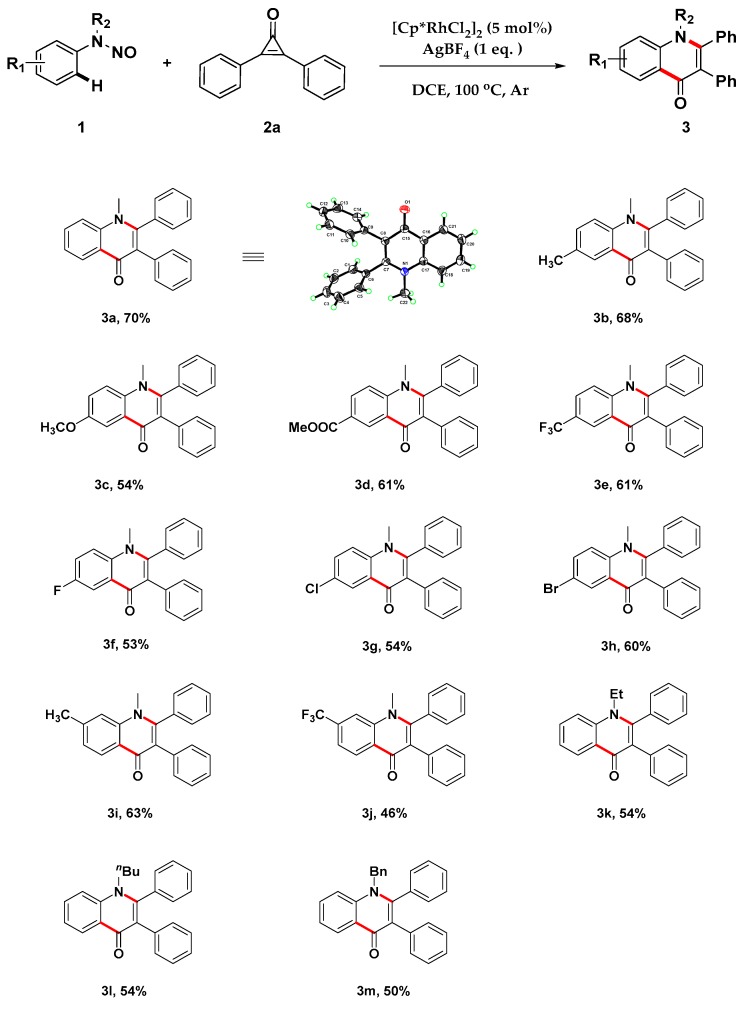
Scope of *N*-nitrosoanilines. Reaction conditions: **1** (0.2 mmol), **2** (0.4 mmol), AgBF_4_ (0.2 mmol), DCE (10 mL), sealed tube under argon, 2 h. The percentage represents isolated yield.

With the optimized reaction conditions in hand, we firstly investigated the scope of *N*-nitrosoanilines, and the results indicated that this formal [3+3] annulation reaction could tolerate various substituents on both the aromatic ring (R_1_) and the nitrogen atom (R_2_) to generate diversified quinolin-4(1*H*)-one derivatives in moderate to good yields (Scheme 2). The introduction of electron-donating groups (CH_3_ and OCH_3_) or electron-withdrawing groups (COOMe and CF_3_) at the 4-position of aniline **1** was tolerant and had no influence on the yields (**3b**–**3h**). Likewise, halogen-substituted anilines were also compatible in this catalytic system, giving the target compounds **3f**–**3h**. When meta-substituted anilines were employed, the C-H bond activation took place at the less sterically hindered position, irrespective of the electronic nature of the substituents, and both electron-donating and electron-withdrawing groups were converted smoothly into the desired products **3i** and **3j**. Additionally, different *N*-substituents were explored, and the results showed that the substrates bearing alkyl and benzylic substituents could afford the desired products in moderate yields (**3k**–**3m**).

**Scheme 3:**
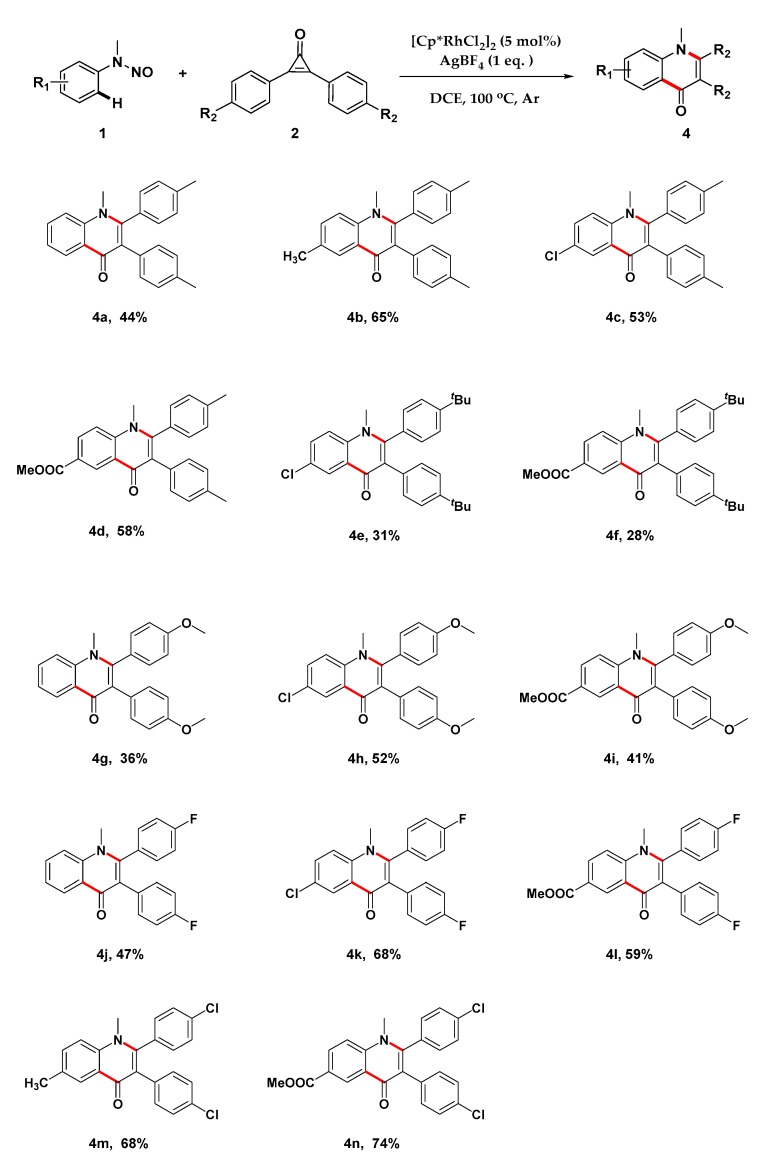
Scope of cyclopropenones. Reaction conditions: **1** (0.2 mmol), **2** (0.3 mmol), AgBF_4_ (0.2 mmol), DCE (10 mL), sealed tube under argon, 2 h. The percentage represents isolated yield.

Next, the scope of cyclopropenones was further tested (Scheme 3), and the results demonstrated that different cyclopropenones could proceed smoothly to provide the corresponding products. The cyclopropenones bearing an electron-donating group at the para position of the phenyl group, such as methyl, *tert*-butyl, and methoxyl, were well tolerated under standard conditions, giving the desired products in moderate to good yields (**4a**–**4i**), regardless of whether electron-donating groups or electron-withdrawing groups were equipped into the *N*-aniline ring. Moreover, halogen-substituted phenyl groups could also be smoothly transformed into the corresponding products in moderate to good yields (**4j**–**4n**).

**Scheme 4:**
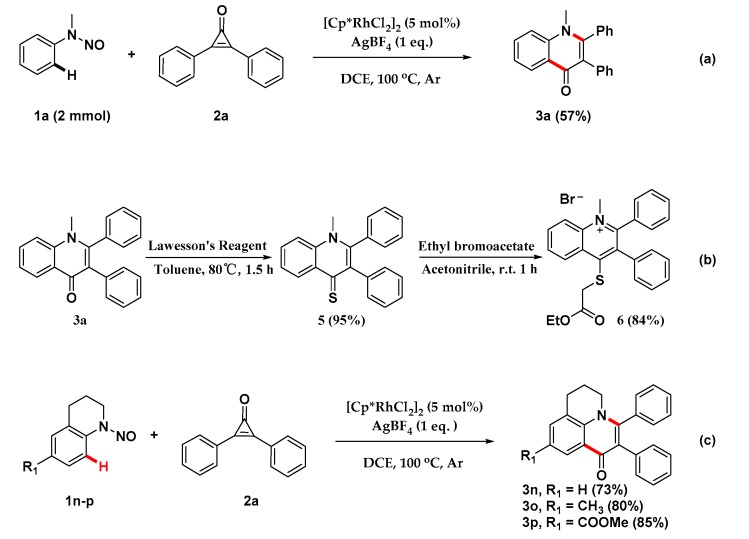
Gram experiment and derivatization of coupled product. (**a**) Gram-scale experiment. (**b**) Derivatization of final product. (**c**) Late-stage functionalization for tetrahydroquinolines.

Intrigued by the privileged heterocyclic product derived from our strategy, we have further explored the gram-scale preparation of this transformation, its synthetic utility, and the late-stage functionalization for some important privileged scaffolds. As shown in Scheme 4, the redox-neutral [3+3] annulation could be carried out on a gram scale to produce **3a** in a 57% yield (Scheme 4a). The synthetic utility of the obtained quinolin-4(1*H*)-one derivatives has been demonstrated by the following transformations into potentially bioactive molecules (Scheme 4b). Treatment of **3a** with Lawesson’s reagent furnished thioketone **5** in a 95% yield, which could be further converted into thio-substituted product **6** in the presence of ethyl bromoacetate with a high yield. More interestingly, this strategy could also be used in the late-stage functionalization for tetrahydroquinoline privileged scaffolds to afford highly fused heterocyclic scaffolds, **3n**–**3p** (Scheme 4c).

**Scheme 5:**
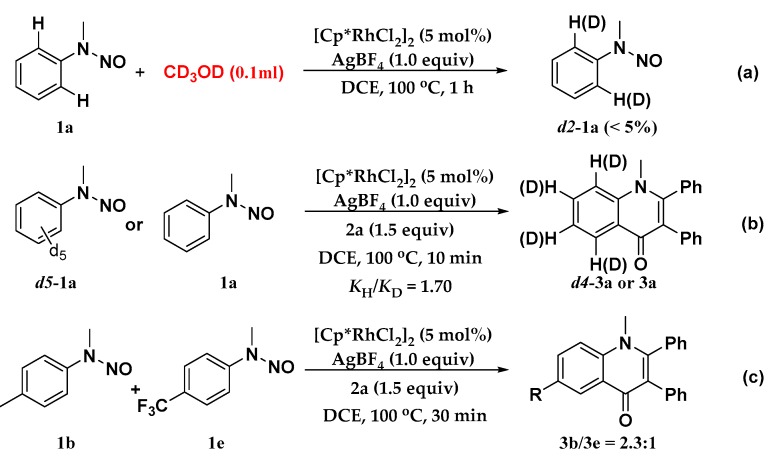
Mechanistic studies. (**a**) Hydrogen–deuterium (H/D) exchange experiment. (**b**) KIE experiment. (**c**) Intermolecular competition experiment.

To understand the reaction mechanism, control experiments were carried out (Scheme 5). Firstly, the hydrogen–deuterium (H/D) exchange experiment was conducted to gain insight into the C-H cleavage step. No deuterated *N*-nitrosoaniline was observed after treating with CD_3_OD, indicating that rhodium-mediated C-H bond cleavage is irreversible (Scheme 5a). D_5_-**1a** and **1a** were then subjected to the standard conditions, and the kinetic isotope effect (KIE) was measured. The value of kH/kD is 1.7, implying that the C-H bond cleavage was the rate-determining step in the transformation (Scheme 5b) [43]. Furthermore, to probe the electronic preference, an intermolecular competition experiment was carried out, and the result suggested that the electron-rich substrate, **1b**, reacted at a higher rate (Scheme 5c).

**Scheme 6:**
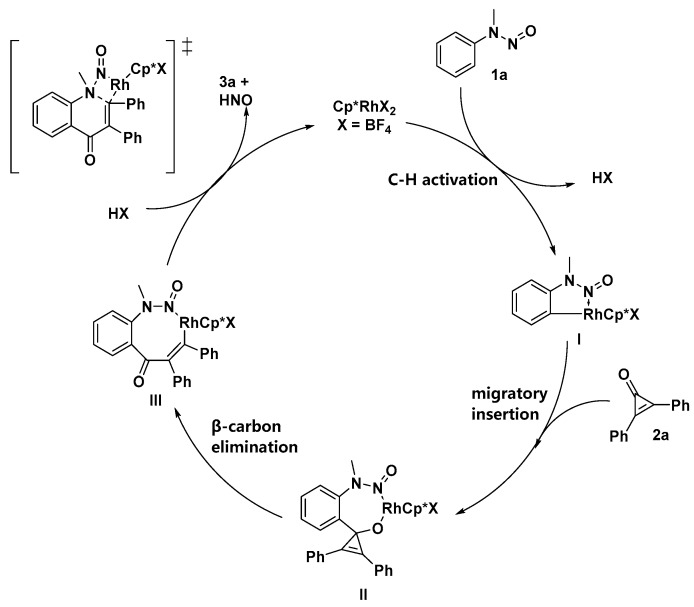
Proposed mechanism.

On the basis of these results and literature precedents [20,21], in order to gain insight into this reaction mechanism, the mechanism of the coupling of *N*-nitrosoaniline with cyclopropenone is proposed in Scheme 6. A cationic Rh(III) species can easily undergo *ortho* C-H insertion of *N*-nitrosoaniline **1a** to afford intermediate **I**. Then, intermediate **I** can be saturated by cyclopropenone coordination and subsequently undergo migratory insertion of the Rh-C bond into the carbonyl group of cyclopropenone **2a** to afford the alkoxide intermediate **II**, which is followed by β-carbon elimination to afford the Rh(III) alkenyl intermediate **III**. Finally, intermediate **III** undergoes a direct cyclization pathway to yield the six-membered ring product, **3a**, releasing HNO with the regeneration of the Rh(III) catalyst.

## 3. Materials and Methods 

### 3.1. General Information

Unless otherwise noted, the reagents (chemicals) were purchased from commercial sources and used without further purification. Water was deionized before being used. Analytical thin layer chromatography (TLC) was HSGF 254 (0.15–0.2 mm thickness). Compound spots were visualized by UV light (254 nm). Column chromatography was performed on silica gel FCP 300–400. NMR spectra were run on a 400 or 500 MHz instrument. Chemical shifts were reported in parts per million (ppm, δ) downfield from tetramethylsilane. Proton coupling patterns are described as singlet (s), doublet (d), triplet (t), quartet (q), multiplet (m), and broad (br). Low- and high-resolution mass spectra (LRMS and HRMS) were measured on a spectrometer. *N*-nitrosoanilines **1** and cyclopropenones **2** were prepared according to the previous literature [23,44,45,46].

### 3.2. General Procedures for Rhodium(III)-Catalyzed Redox-Neutral [3+3] Annulation of N-nitrosoanilines with Cyclopropenones (***3*** and ***4***)

To a 35 mL Schlenk tube was sequentially added *N*-nitrosoanilines **1** (0.2 mmol), cyclopropenone **2** (0.4 mmol for product **3**, 0.3 mmol for product **4**), catalyst (5 mol%), Ag salt (0.2 mmol), and solvent (10 mL). The reaction was sealed under argon and stirred at 100 °C for 2 h. After the reaction was completed (detected by TLC), solvent was removed under reduced pressure, and the crude mixture was purified by flash column chromatography on silica gel with a PE/EA (4/1, *v*/*v*) solvent system to afford the final product, **3**, and with a CH_2_Cl_2_/CH_3_OH (50/1, *v*/*v*) solvent system to afford the final product, **4**.

### 3.3. General Procedures for 1-Methyl-2,3-diphenylquinoline-4(1H)-thione (***5***)

To a solution of **3a** (50 mg, 0.16 mmol) in 20 mL toluene was added Lawesson’s reagent (64 mg, 0.16 mmol), and the reaction was stirred at 80 °C for 1.5 h. After the reaction was completed, the mixture was filtered, and the precipitate was washed with cold ethanol to afford the compound, **5**, as a brown solid in a 95% yield.

### 3.4. General Procedures for 4-((2-Ethoxy-2-oxoethyl)thio)-1-methyl-2,3-diphenylquinolin-1-ium (***6***)

A 50 mL reaction flask was charged with acetonitrile and compound **5** (20 mg, 0.06 mmol), and ethyl bromoacetate (1.2 equiv) was added for 1 h at room temperature. After the reaction was completed, the solvent was removed, and the residue was purified by flash column chromatography on silica gel eluting with methanol to afford the compound, **6**, in a 84% yield.

### 3.5. Analytical Characterization Data of Products

*1-Methyl-2,3-diphenylquinolin-4(1H)-one* (**3a**). Light yellow solid (70%). m.p. 218.3–218.8 °C; ^1^H NMR (500 MHz, Chloroform-*d*) δ 8.59 (dd, *J* = 8.0, 1.7 Hz, 1H), 7.73 (ddd, *J* = 8.7, 7.0, 1.7 Hz, 1H), 7.57 (d, *J* = 8.6 Hz, 1H), 7.46–7.41 (m, 1H), 7.30–7.26 (m, 3H), 7.18–7.14 (m, 2H), 7.13–7.09 (m, 2H), 7.07–7.02 (m, 3H), 3.55 (s, 3H); ^13^C NMR (125 MHz, Chloroform-*d*) δ 176.4, 152.2, 141.6, 135.9, 135.2, 132.4, 131.5, 129.7, 128.8, 128.4, 127.6, 127.6, 126.8, 126.2, 124.5, 123.7, 115.9, 37.8. IR (KBr, cm^−1^): 3056, 1616, 1531, 1482, 1321, 1068, 754. HRMS (Electrospray ionization ESI) *m*/*z* [M + H]^+^ calcd. for C_22_H_18_NO: 312.1383, found: 312.1384.

*1,6-Dimethyl-2,3-diphenylquinolin-4(1H)-one* (**3b**). Light yellow solid (68%). m.p. 187.5–187.8 °C; ^1^H NMR (400 MHz, Chloroform-*d*) δ 8.39–8.35 (m, 1H), 7.55 (dd, *J* = 8.7, 2.2 Hz, 1H), 7.48 (d, *J* = 8.8 Hz, 1H), 7.29–7.26 (m, 3H), 7.17–7.08 (m, 4H), 7.06–7.01 (m, 3H), 3.53 (s, 3H), 2.52 (s, 3H); ^13^C NMR (125 MHz, Chloroform-*d*) δ 176.3, 151.9, 139.7, 136.2, 135.3, 133.8, 133.6, 131.6, 129.8, 128.8, 128.4, 127.6, 126.9, 126.7, 126.2, 124.2, 115.8, 37.7, 21.1. HRMS (ESI) *m*/*z* [M + H]^+^ calcd. for C_23_H_20_NO: 326.1539, found: 326.1539.

*6-Methoxy-1-methyl-2,3-diphenylquinolin-4(1H)-one* (**3c**). Yellow solid (54%). m.p. 212.4–212.7 °C; ^1^H NMR (400 MHz, Chloroform-*d*) δ 8.00 (d, *J* = 3.1 Hz, 1H), 7.54 (d, *J* = 9.3 Hz, 1H), 7.37–7.35 (m, 1H), 7.29–7.28 (m, 3H), 7.18–7.08 (m, 4H), 7.07–7.01 (m, 3H), 3.95 (s, 3H), 3.56 (s, 3H); ^13^C NMR (125 MHz, Chloroform-*d*) δ 175.9, 156.7, 151.8, 136.5, 136.4, 135.4, 131.8, 130.0, 129.1, 128.7, 128.1, 127.8, 126.5, 123.8, 123.3, 117.9, 106.7, 56.2, 38.2. HRMS (ESI) *m*/*z* [M + H]^+^ calcd. for C_23_H_20_NO_2_: 342.1489, found: 342.1495.

*6-Methoxycarbonyl-1-methyl-2,3-diphenylquinoline-4(1H)-one* (**3d**). Light yellow solid (61%). m.p. 236.7–237.2 °C; ^1^H NMR (600 MHz, DMSO-d6) δ 8.86 (d, J = 2.3 Hz, 1H), 8.27 (dd, J = 9.0, 2.2 Hz, 1H), 7.92 (d, *J* = 9.1 Hz, 1H), 7.23–7.06 (m, 4H), 6.94–6.91 (m, 2H), 6.88–6.85 (m, 2H), 3.92 (s, 3H), 3.47 (s, 3H), 2.27 (s, 3H), 2.19 (s, 3H); ^13^C NMR (125 MHz, Chloroform-*d*) δ 176.3, 166.7, 152.6, 144.2, 135.3, 134.8, 132.8, 131.3, 130.1, 129.6, 129.1, 128.6, 127.7, 126.5, 126.2, 125.6, 125.3, 116.2, 52.4, 38.1. HRMS (ESI) *m*/*z* [M + H]^+^ calcd. for C_24_H_20_NO_3_: 370.1438, found: 370.1430.

*6-(Trifluoromethyl)-1-methyl-2,3-diphenyl-quinolin-4(1H)-one* (**3e**). Light yellow solid (61%). m.p. 226.7–227.0 °C; ^1^H NMR (400 MHz, Chloroform-*d*) δ 8.86 (d, *J* = 2.2 Hz, 1H), 7.95–7.90 (m, 1H), 7.67 (d, *J* = 9.0 Hz, 1H), 7.33–7.27 (m, 3H), 7.18–7.06 (m, 5H), 7.06–6.98 (m, 2H), 3.57 (s, 3H). ^13^C NMR (125 MHz, Chloroform-*d*) δ 175.9, 152.9, 143.3, 135.2, 134.6, 131.3, 129.6, 129.2, 128.6, 128.5 (q, *J* = 3.0 Hz) 127.7, 126.6, 126.3, 125.9, 125.6 (q, *J* = 3.0 Hz), 125.1, 123.3 (q, *J* = 271.8 Hz), 116.9, 38.0. ^19^F NMR (471 MHz, Chloroform-*d*) δ -61.9. HRMS (ESI) *m*/*z* [M + H]+ calcd. for C_23_H_17_F_3_NO: 380.1257, found 380.1251.

*6-Fluoro-1-methyl-2,3-diphenylquinolin-4(1H)-one* (**3f**). White solid (53%). m.p. 213.5–213.8 °C; ^1^H NMR (500 MHz, Chloroform-*d*) δ 8.22 (dd, *J* = 8.9, 3.1 Hz, 1H), 7.58 (dd, *J* = 9.3, 4.1 Hz, 1H), 7.46 (ddd, *J* = 9.3, 7.5, 3.1 Hz, 1H), 7.30–7.27 (m, 3H), 7.17–7.09 (m, 4H), 7.08–7.00 (m, 3H), 3.56 (s, 3H); ^13^C NMR (125 MHz, Chloroform-*d*) δ 175.6, 159.4 (d, *J* = 243.9 Hz), 152.3, 138.1, 135.7, 134.9, 131.4, 129.7, 129.0, 128.5, 128.3 (d, *J* = 6.8 Hz), 127.7, 126.4, 123.9, 120.9 (d, *J* = 24.8 Hz), 118.2 (d, *J* = 7.6 Hz), 112.1 (d, *J* = 22.3 Hz), 38.1. ^19^F NMR (471 MHz, Chloroform-*d*) δ -118.0. HRMS (ESI) *m*/*z* [M + H]^+^ calcd. for C_22_H_17_FNO: 330.1289, found: 330.1288.

*6-Chloro-1-methyl-2,3-diphenylquinolin-4(1H)-one* (**3g**). Light yellow solid (54%). m.p. 248.4–248.9 °C; ^1^H NMR (500 MHz, Chloroform-*d*) δ 8.53 (d, *J* = 2.5 Hz, 1H), 7.66 (dd, *J* = 9.1, 2.6 Hz, 1H), 7.52 (d, *J* = 9.1 Hz, 1H), 7.30–7.27 (m, 3H), 7.18–7.09 (m, 4H), 7.09–6.98 (m, 3H), 3.54 (s, 3H); ^13^C NMR (125 MHz, Chloroform-*d*) δ 175.3, 152.4, 140.0, 135.5, 134.9, 132.6, 131.4, 130.0, 129.7, 129.0, 128.5, 127.8, 127.7, 126.9, 126.5, 124.8, 117.7, 38.0. HRMS (ESI) *m*/*z* [M + H]^+^ calcd. for C_22_H_17_ClNO: 346.0993, found: 346.0996.

*6-Bromo-1-methyl-2,3-diphenylquinolin-4(1H)-one* (**3h**). Light yellow solid (60%). m.p. 243.1–244.2 °C; ^1^H NMR (500 MHz, Chloroform-*d*) δ 8.69 (d, *J* = 2.5 Hz, 1H), 7.79 (dd, *J* = 9.0, 2.5 Hz, 1H), 7.45 (d, *J* = 9.1 Hz, 1H), 7.30–7.28 (m, 3H), 7.16–7.09 (m, 4H), 7.08–6.99 (m, 3H), 3.53 (s, 3H); ^13^C NMR (125 MHz, Chloroform-*d*) δ 175.2, 152.4, 140.4, 135.5, 135.3, 134.8, 131.4, 130.1, 129.7, 129.0, 128.5, 128.2, 127.7, 126.5, 125.0, 117.9, 117.6, 37.9. HRMS (ESI) *m*/*z* [M + H]^+^ calcd. for C_22_H_17_Br NO: 390.0488, found: 390.0482.

*1,7-Dimethyl-2,3-diphenylquinolin-4(1H)-one* (**3i**). Light yellow solid (63%). m.p. 286.1–287.6 °C; ^1^H NMR (500 MHz, Chloroform-*d*) δ 8.46 (d, *J* = 8.2 Hz, 1H), 7.35 (s, 1H), 7.30–7.26 (m, 4H), 7.16–7.14 (m, 2H), 7.12–7.08 (m, 2H), 7.06–7.01 (m, 3H), 3.52 (s, 3H), 2.56 (s, 3H); ^13^C NMR (125 MHz, Chloroform-*d*) δ 176.3, 151.9, 143.1, 141.8, 136.0, 135.3, 131.6, 129.8, 128.8, 128.4, 127.5, 127.4, 126.2, 125.4, 124.8, 124.3, 115.6, 37.7, 22.5. HRMS (ESI) *m*/*z* [M + H]^+^ calcd. for C_23_H_20_NO: 326.1539, found: 326.1546.

*1-Methyl-2,3-diphenyl-7-(trifluoromethyl)quinolin-4(1H)-one* (**3j**). Light yellow solid (46%). m.p. 221.4–222.7 °C; ^1^H NMR (500 MHz, Chloroform-*d*) δ 8.69 (d, *J* = 8.3 Hz, 1H), 7.84 (s, 1H), 7.65 (dd, *J* = 8.4, 1.4 Hz, 1H), 7.32–7.28 (m, 3H), 7.18–7.11 (m, 4H), 7.09–7.00 (m, 3H), 3.59 (s, 3H); ^13^C NMR (125 MHz, Chloroform-*d*) δ 175.8, 153.1, 141.2, 135.3, 134.7, 134.0 (q, *J* = 32.5 Hz), 131.3, 129.6, 129.2, 129.0, 128.6, 127.7, 126.6, 125.6, 124.0 (q, *J* = 262.5 Hz), 119.7 (q, *J* = 3.2 Hz), 113.6 (q, *J* = 4.2 Hz), 38.0. ^19^F NMR (471 MHz, Chloroform-*d*) δ -62.6. HRMS (ESI) *m*/*z* [M + H]^+^ calcd. for C_23_H_17_F_3_NO: 380.1257, found: 380.1252.

*1-Ethyl-2,3-diphenylquinolin-4(1H)-one* (**3k**). White solid (54%). m.p. 247.4–247.9 °C; ^1^H NMR (400 MHz, Chloroform-*d*) δ 8.61 (d, *J* = 8.0 Hz, 1H), 7.75–7.68 (m, 1H), 7.60 (d, *J* = 8.7 Hz, 1H), 7.43 (t, *J* = 7.5 Hz, 1H), 7.29–7.26 (m, 3H), 7.22–7.17 (m, 2H), 7.13–7.07 (m, 2H), 7.06–7.00 (m, 3H), 4.08 (q, *J* = 7.0 Hz, 2H), 1.30 (t, *J* = 7.0 Hz, 3H); ^13^C NMR (150 MHz, Chloroform-*d*) δ 176.5, 152.2, 140.3, 136.2, 135.2, 132.6, 131.6, 129.6, 129.0, 128.6, 128.2, 127.8, 127.4, 126.5, 125.0, 123.8, 116.3, 43.9, 14.7. HRMS (ESI) *m*/*z* [M + H]^+^ calcd. for C_23_H_20_NO: 326.1539, found: 326.1532.

*1-Butyl-2,3-diphenylquinolin-4(1H)-one* (**3l**). White solid (54%). m.p. 119.1–120.4 °C; ^1^H NMR (500 MHz, Chloroform-*d*) δ 8.61 (dd, *J* = 8.1, 1.7 Hz, 1H), 7.71 (ddd, *J* = 8.7, 7.0, 1.7 Hz, 1H), 7.56 (d, *J* = 8.7 Hz, 1H), 7.44–7.40 (m, 1H), 7.30–7.27 (m, 3H), 7.19–7.16 (m, 2H), 7.10 (dd, *J* = 8.3, 6.5 Hz, 2H), 7.06–7.00 (m, 3H), 3.99–3.92 (m, 2H), 1.70–1.68 (m, 2H), 1.20–1.13 (m, 2H), 0.77 (t, *J* = 7.4 Hz, 3H); ^13^C NMR (125 MHz, Chloroform-*d*) δ 176.2, 152.1, 140.3, 136.0, 135.0, 132.3, 131.5, 129.5, 128.8, 128.3, 127.9, 127.6, 127.1, 126.3, 124.7, 123.6, 116.2, 48.7, 30.9, 19.9, 13.5. HRMS (ESI) *m*/*z* [M + H]^+^ calcd. for C_25_H_24_NO: 354.1852, found: 354.1857.

*1-Benzyl-2,3-diphenylquinolin-4(1H)-one* (**3m**). Light yellow solid (50%). m.p. 74.3–75.6 °C; ^1^H NMR (500 MHz, Chloroform-*d*) δ 8.60 (dd, *J* = 8.0, 1.7 Hz, 1H), 7.55 (ddd, *J* = 8.7, 7.0, 1.7 Hz, 1H), 7.41–7.35 (m, 2H), 7.32–7.26 (m, 3H), 7.19–7.02 (m, 10H), 7.01–6.98 (m, 2H), 5.25 (s, 2H); ^13^C NMR (125 MHz, Chloroform-*d*) δ 176.6, 152.6, 140.9, 136.6, 135.9, 134.7, 132.4, 131.5, 129.4, 129.1, 128.9, 128.2, 127.7, 127.6, 127.5 127.0, 126.3, 125.7, 124.9, 123.8, 117.2, 52.7. HRMS (ESI) *m*/*z* [M + H]^+^ calcd. for C_28_H_22_NO: 388.1696, found: 388.1698.

*2,3-Diphenyl-6,7-dihydro-1H,5H-pyrido [3,2,1-ij]quinolin-1-one* (**3n**). Yellow solid (73%). m.p. 280.2–281.4 °C; ^1^H NMR (500 MHz, Chloroform-*d*) δ 8.42 (dd, *J* = 8.1, 1.6 Hz, 1H), 7.45 (dd, *J* = 7.1, 1.5 Hz, 1H), 7.33–7.24 (m, 4H), 7.18–7.13 (m, 2H), 7.13–7.07 (m, 2H), 7.06–7.00 (m, 3H), 3.82–3.75 (m, 2H), 3.08–3.04 (m, 2H), 2.12–2.05 (m, 2H); ^13^C NMR (125 MHz, Chloroform-*d*) δ 176.3, 151.3, 138.2, 136.0, 134.9, 131.5, 131.5, 129.5, 128.7, 128.4, 127.5, 126.9, 126.8, 126.2, 125.5, 124.1, 123.2, 50.2, 28.0, 22.1. IR (KBr, cm^−1^): 3045, 1616, 1544, 1484, 1438, 1307, 1039, 703. HRMS (ESI) *m*/*z* [M + H]^+^ calcd. for C_24_H_20_NO: 338.1539, found: 338.1536.

*9-Methyl-2,3-diphenyl-6,7-dihydro-1H,5H-pyrido[3,2,1-ij]quinolin-1-one* (**3o**). Yellow solid (80%). m.p. 251.2–252.3 °C; ^1^H NMR (500 MHz, Chloroform-*d*) δ 8.22–8.17 (m, 1H), 7.30–7.26 (m, 2H), 7.26–7.23 (m, 2H), 7.16–7.07 (m, 4H), 7.05–7.00 (m, 3H), 3.81–3.70 (m, 2H), 3.81–3.70 (m, 2H), 2.46 (s, 3H), 2.11–2.03 (m, 2H); ^13^C NMR (125 MHz, Chloroform-*d*) δ 176.1, 150.9, 136.3, 136.2, 134.9, 133.0, 133.0, 131.6, 129.5, 128.6, 128.4, 127.5, 126.8, 126.7, 126.1, 124.7, 123.8, 50.1, 27.9, 22.2, 21.1. IR (KBr, cm^−1^): 3056, 1625, 1536, 1490, 1317, 1274, 1079, 711. HRMS (ESI) *m*/*z* [M + H]^+^ calcd. for C_25_H_22_NO: 352.1696, found: 352.1697.

*9-Methoxycarbonyl-2,3-diphenyl-6,7-dihydro-1H,5H-pyrido[3,2,1-ij]quinolin-1-one* (**3p**). Yellow solid (85%). m.p. 249.1–250.7 °C; ^1^H NMR (400 MHz, Chloroform-*d*) δ9.06–9.03 (m, 1H), 8.09–8.06 (m, 1H), 7.30–7.26 (m, 3H), 7.18–7.08 (m, 4H), 7.08–6.99 (m, 3H), 3.95 (s, 3H), 3.82–3.75 (m, 2H), 3.12–3.06 (m, 2H), 2.14–2.05 (m, 2H); ^13^C NMR (125 MHz, Chloroform-*d*) δ 176.3, 166.9, 151.8, 141.0, 135.4, 134.4, 131.5, 131.3, 129.4, 128.9, 128.6, 128.1, 127.6, 127.3, 126.5, 126.2, 125.2, 124.6, 52.3, 50.4, 27.9, 21.8. IR (KBr, cm^−1^): 3057, 1714, 1627, 1498, 1442, 1301, 1215, 1072, 701. HRMS (ESI) *m*/*z* [M + H]^+^ calcd. for C_26_H_22_NO_3_: 396.1594, found: 396.1593.

*1-Methyl-2,3-di-p-tolylquinolin-4(1H)-one* (**4a**). White solid (44%). m.p. 186.3–186.7 °C; ^1^H NMR (500 MHz, Chloroform-*d*) δ 8.59 (dd, *J* = 8.0, 1.7 Hz, 1H), 7.74–7.67 (m, 1H), 7.56 (d, *J* = 8.6 Hz, 1H), 7.42 (ddd, *J* = 8.0, 7.0, 0.9 Hz, 1H), 7.09 (d, *J* = 7.8 Hz, 2H), 7.04 (d, *J* = 8.1 Hz, 2H), 6.94–6.91 (m, 4H), 3.53 (s, 3H), 2.32 (s, 3H), 2.22 (s, 3H); ^13^C NMR (125 MHz, Chloroform-*d*) δ 176.7, 152.6, 141.9, 138.9, 135.8, 133.1, 132.6, 132.5, 131.5, 129.8, 129.4, 128.6, 127.9, 126.9, 124.7, 123.9, 116.1, 38.0, 21.7, 21.6. HRMS (ESI) *m*/*z* [M + H]^+^ calcd. for C_24_H_22_NO: 340.1696, found: 340.1694.

*1,6-Dimethyl-2,3-di-p-tolylquinolin-4(1H)-one* (**4b**). White solid (65%). m.p. 233.7–233.9 °C; ^1^H NMR (500 MHz, Chloroform-*d*) δ 8.39–8.33 (m, 1H), 7.52 (dd, *J* = 8.8, 2.1 Hz, 1H), 7.45 (d, *J* = 8.7 Hz, 1H), 7.08 (d, *J* = 7.9 Hz, 2H), 7.02 (d, *J* = 8.1 Hz, 2H), 6.92–6.90 (m, 4H), 3.50 (s, 3H), 2.50 (s, 3H), 2.31 (s, 3H), 2.22 (s, 3H); ^13^C NMR (125 MHz, Chloroform-*d*) δ 176.1, 151.7, 139.4, 138.3, 135.2, 133.4, 133.1, 132.8, 132.2, 131.1, 129.4, 128.8, 128.1, 126.7, 126.3, 123.9, 115.5, 37.4, 21.2, 21.1, 20.8. HRMS (ESI) *m*/*z* [M + H]^+^ calcd. for C_25_H_24_NO: 354.1852, found: 354.1850.

*6-Chloro-1-methyl-2,3-di-p-tolylquinolin-4(1H)-one* (**4c**). Light yellow solid (53%). m.p. 240.2–241.7 °C; ^1^H NMR (600 MHz, DMSO-d6) δ 8.19 (d, *J* = 2.5 Hz, 1H), 7.87 (d, *J* = 9.1 Hz, 1H), 7.83 (dd, *J* = 9.1, 2.6 Hz, 1H), 7.18–7.12 (m, 4H), 6.93–6.89 (m, 2H), 6.87–6.83 (m, 2H), 3.45 (s, 3H), 2.26 (s, 3H), 2.18 (s, 3H); ^13^C NMR (150 MHz, Chloroform-*d*) δ 175.7, 152.7, 140.2, 139.1, 136.0, 132.7, 132.6, 132.2, 131.3, 129.9, 129.7, 129.4, 128.6, 127.9, 126.9, 124.9, 118.0, 38.1, 21.7, 21.6. HRMS (ESI) *m*/*z* [M + H]^+^ calcd. for C_24_H_21_ClNO: 374.1306, found: 374.1305.

*6-Methoxycarbonyl-1-methyl-2,3-di-p-tolylquinolin-4(1H)-one* (**4d**). White solid (58%). m.p. 238.1–239.4 °C; ^1^H NMR (600 MHz, DMSO-d6) δ 8.86 (d, *J* = 2.3 Hz, 1H), 8.27 (dd, *J* = 9.0, 2.2 Hz, 1H), 7.92 (d, *J* = 9.1 Hz, 1H), 7.23–7.06 (m, 4H), 6.94–6.91 (m, 2H), 6.88–6.85 (m, 2H), 3.92 (s, 3H), 3.47 (s, 3H), 2.27 (s, 3H), 2.19 (s, 3H); ^13^C NMR (150 MHz, Chloroform-*d*) δ 176.7, 167.0, 152.8, 144.4, 139.1, 136.1, 132.8, 132.5, 132.2, 131.3, 130.3, 129.7, 129.5, 128.7, 126.2, 125.7, 125.3, 116.4, 52.6, 38.2, 21.7, 21.6. HRMS (ESI) *m*/*z* [M + H]^+^ calcd. for C_26_H_24_NO_3_: 398.1751, found: 398.1744.

*2,3-Bis(4-(tert-butyl)phenyl)-6-chloro-1-methylquinolin-4(1H)-one* (**4e**). White solid (31%). m.p. 296.0–297.6 °C; ^1^H NMR (500 MHz, Chloroform-*d*) δ 8.56 (d, *J* = 2.5 Hz, 1H), 7.66 (dd, *J* = 9.1, 2.6 Hz, 1H), 7.54 (d, *J* = 9.0 Hz, 1H), 7.28 (d, *J* = 6.2 Hz, 2H), 7.10 (d, *J* = 8.0 Hz, 2H), 7.04 (d, *J* = 8.1 Hz, 2H), 6.91 (d, *J* = 8.0 Hz, 2H), 3.62 (s, 3H), 1.27 (s, 9H), 1.22 (s, 9H); ^13^C NMR (150 MHz, Chloroform-*d*) δ 175.5, 153.1, 152.2, 148.9, 140.1, 132.8, 132.6, 132.1, 131.1, 130.0, 129.7, 128.0, 127.0, 125.3, 125.1, 124.6, 117.9, 38.3, 35.0, 34.6, 31.6, 31.5. HRMS (ESI) *m*/*z* [M + H]^+^ calcd. for C_30_H_33_ClNO: 458.2245, found: 458.2248.

*2,3-Bis(4-(tert-butyl)phenyl)-6-methoxycarbonyl-1-methylquinolin-4(1H)-one* (**4f**). White solid (28%). m.p. 273.8–274.5 °C; ^1^H NMR (500 MHz, Chloroform-*d*) δ 9.23 (d, *J* = 2.2 Hz, 1H), 8.35 (dd, *J* = 9.0, 2.2 Hz, 1H), 7.61 (d, *J* = 9.0 Hz, 1H), 7.28 (d, *J* = 2.4 Hz, 2H), 7.12–7.08 (m, 2H), 7.06–7.03 (m, 2H), 6.93–6.89 (m, 2H), 3.99 (s, 3H), 3.64 (s, 3H), 1.27 (s, 9H), 1.21 (s, 9H); ^13^C NMR (150 MHz, Chloroform-*d*) δ 176.5, 167.0, 153.2, 152.3, 149.0, 144.4, 132.8, 132.6, 132.1, 131.1, 130.3, 129.7, 126.4, 125.9, 125.4, 125.3, 124.6, 116.4, 52.6, 38.4, 35.0, 34.6, 31.6, 31.5. HRMS (ESI) *m*/*z* [M + H]^+^ calcd. for C_32_H_36_NO_3_: 482.269, found: 482.2686.

*2,3-Bis(4-methoxyphenyl)-1-methylquinolin-4(1H)-one* (**4g**). White solid (36%). m.p. 192.7–193.2 °C; ^1^H NMR (500 MHz, Chloroform-*d*) δ 8.57 (d, *J* = 8.1 Hz, 1H), 7.71 (t, *J* = 8.0 Hz, 1H), 7.55 (d, *J* = 8.7 Hz, 1H), 7.42 (t, *J* = 7.5 Hz, 1H), 7.06 (d, *J* = 8.1 Hz, 2H), 6.96 (d, *J* = 8.2 Hz, 2H), 6.81 (d, *J* = 8.1 Hz, 2H), 6.68 (d, *J* = 8.1 Hz, 2H), 3.79 (s, 3H), 3.73 (s, 3H), 3.55 (s, 3H);^13^C NMR (125 MHz, Chloroform-*d*) δ 176.9, 159.9, 158.1, 152.4, 141.9, 132.8, 132.5, 131.3, 128.7, 127.9, 127.8, 127.0, 124.5, 123.8, 116.2, 114.2, 113.5, 55.6, 55.5, 38.1. HRMS (ESI) *m*/*z* [M + H]^+^ calcd. for C_24_H_22_NO_3_: 372.1594, found: 372.1596.

*6-Chloro-2,3-bis(4-methoxyphenyl)-1-methylquinolin-4(1H)-one* (**4h**). White solid (52%). m.p. 241.5–242.1 °C; ^1^H NMR (500 MHz, Chloroform-*d*) δ 8.51 (d, *J* = 2.5 Hz, 1H), 7.63 (dd, *J* = 9.1, 2.6 Hz, 1H), 7.50 (d, *J* = 9.1 Hz, 1H), 7.05 (d, *J* = 8.6 Hz, 2H), 6.94 (d, *J* = 8.6 Hz, 2H), 6.81 (d, *J* = 8.3 Hz, 2H), 6.68 (d, *J* = 8.3 Hz, 2H), 3.79 (s, 3H), 3.73 (s, 3H), 3.53 (s, 3H); ^13^C NMR (125 MHz, Chloroform-*d*) δ 175.8, 160.1, 158.2, 152.7, 140.3, 132.7, 132.7, 131.3, 130.0, 128.3, 128.0, 127.5, 127.0, 124.8, 118.0, 114.2, 113.6, 55.6, 55.5, 38.2. HRMS (ESI) *m*/*z* [M + H]^+^ calcd. for C_24_H_21_ClNO_3_: 406.1204, found: 406.1203.

*6-Methoxycarbonyl-2,3-bis(4-methoxyphenyl)-1-methylquinolin-4(1H)-one* (**4i**). White solid (41%). m.p. 242.3–243.7 °C; ^1^H NMR (500 MHz, Chloroform-*d*) δ 9.19 (d, *J* = 2.2 Hz, 1H), 8.32 (dd, *J* = 9.0, 2.2 Hz, 1H), 7.58 (d, *J* = 9.0 Hz, 1H), 7.10–7.03 (m, 2H), 6.98–6.92 (m, 2H), 6.85–6.78 (m, 2H), 6.73–6.66 (m, 2H), 3.96 (s, 3H), 3.79 (s, 3H), 3.73 (s, 3H), 3.55 (s, 3H); ^13^C NMR (125 MHz, Chloroform-*d*) δ 176.8, 167.0, 160.1, 158.2, 152.8, 144.5, 132.9, 132.7, 131.3, 130.4, 128.1, 127.5, 126.3, 125.6, 125.4, 116.5, 114.3, 113.6, 55.6, 55.5, 52.6, 38.3. HRMS (ESI) *m*/*z* [M + H]^+^ calcd. for C_26_H_24_NO_5_: 430.1649, found: 430.1648.

*2,3-Bis(4-fluorophenyl)-1-methylquinolin-4(1H)-one* (**4j**). White solid (47%). m.p. 239.2–241.8 °C; ^1^H NMR (500 MHz, Chloroform-*d*) δ 8.56 (dd, *J* = 8.1, 1.7 Hz, 1H), 7.74 (ddd, *J* = 8.6, 6.9, 1.6 Hz, 1H), 7.57 (d, *J* = 8.6 Hz, 1H), 7.45 (t, *J* = 7.6 Hz, 1H), 7.17–7.10 (m, 2H), 7.05–6.93 (m, 4H), 6.83 (t, *J* = 8.8 Hz, 2H), 3.55 (s, 3H); ^13^C NMR (125 MHz, Chloroform-*d*) δ 176.4, 162.7 (d, *J* = 248.9 Hz), 161.5 (d, *J* = 243.6 Hz), 151.3, 141.6, 133.1 (d, *J* = 8.0 Hz), 132.6, 131.7 (d, *J* = 8.2 Hz), 131.1 (d, *J* = 3.7 Hz), 129.0 (d, J = 4.6 Hz), 127.6, 126.7, 124.0, 123.7, 116.0, 115.9 (d, *J* = 13.2 Hz), 114.8 (d, *J* = 21.2 Hz), 37.8. ^19^F NMR (471 MHz, Chloroform-*d*) δ −111.0, −116.2. HRMS (ESI) *m*/*z* [M + H]^+^ calcd. for C_22_H_16_F_2_NO: 348.1194, found: 348.1192.

*6-Chloro-2,3-bis(4-fluorophenyl)-1-methylquinolin-4(1H)-one* (**4k**). White solid (68%). m.p. 293.3–294.2 °C; ^1^H NMR (500 MHz, Chloroform-*d*) δ 8.51 (d, *J* = 2.6 Hz, 1H), 7.67 (dd, *J* = 9.1, 2.6 Hz, 1H), 7.52 (d, *J* = 9.1 Hz, 1H), 7.17–7.10 (m, 2H), 7.06–6.93 (m, 4H), 6.84 (t, *J* = 8.7 Hz, 2H), 3.54 (s, 3H); ^13^C NMR (125 MHz, Chloroform-*d*) δ 175.2, 162.8 (d, *J* = 249.3 Hz), 161.5 (d, *J* = 244.1 Hz), 151.5, 140.0, 133.0 (d, *J* = 8.0 Hz), 132.8, 131.6 (d, *J* = 8.2 Hz), 131.3 (d, *J* = 3.5 Hz), 130.8 (d, *J* = 3.7 Hz), 130.3, 126.8, 124.1, 117.8, 116.0 (d, *J* = 21.7 Hz), 114.9 (d, *J* = 213 Hz), 114.8, 38.0. ^19^F NMR (471 MHz, Chloroform-*d*) δ -110.6, -115.8. HRMS (ESI) *m*/*z* [M + H]^+^ calcd. for C_22_H_15_ClF_2_NO: 382.0805, found: 382.0795.

*6-Methoxycarbonyl-2,3-bis(4-fluorophenyl)-1-methylquinolin-4(1H)-one* (**4l**). White solid (59%). m.p. 213.5–214.2 °C; ^1^H NMR (500 MHz, Chloroform-*d*) δ 9.19 (d, *J* = 2.1 Hz, 1H), 8.35 (dd, *J* = 9.0, 2.2 Hz, 1H), 7.60 (d, *J* = 9.0 Hz, 1H), 7.17–7.12 (m, 2H), 7.06–6.94 (m, 4H), 6.84 (t, *J* = 8.7 Hz, 2H), 3.97 (s, 3H), 3.57 (s, 3H); ^13^C NMR (125 MHz, Chloroform-*d*) δ 176.5, 166.8, 163.1 (d, *J* = 249.3 Hz), 161.0 (d, *J* = 244.3 Hz), 152.0, 144.4, 133.3 (d, *J* = 9.4 Hz), 133.1, 131.9 (d, *J* = 8.3 Hz), 131.3 (d, *J* = 3.2 Hz), 130.9 (d, *J* = 3.6 Hz), 130.3, 126.3, 125.9, 125.1, 116.5 (d, *J* = 14.1 Hz), 116.2, 115.2 (d, *J* = 21.3 Hz), 52.7, 38.4. ^19^F NMR (471 MHz, Chloroform-*d*) δ −110.5, −115.7. HRMS (ESI) *m*/*z* [M + H]^+^ calcd. for C_24_H_18_F_2_NO_3_: 406.1249, found: 406.1247.

*2,3-Bis(4-chlorophenyl)-1,6-dimethylquinolin-4(1H)-one* (**4m**). White solid (68%). m.p. 226.1–227.3 °C; ^1^H NMR (500 MHz, Chloroform-*d*) δ 8.35–8.32 (m, 1H), 7.56 (dd, *J* = 8.7, 2.3 Hz, 1H), 7.47 (d, *J* = 8.8 Hz, 1H), 7.32–7.28 (m, 2H), 7.14–7.07 (m, 4H), 6.99–6.92 (m, 2H), 3.52 (s, 3H), 2.51 (s, 3H); ^13^C NMR (125 MHz, Chloroform-*d*) δ 176.3, 150.9, 139.9, 135.5, 134.6, 134.4, 134.3, 133.6, 133.1, 132.6, 131.3, 129.3, 128.3, 127.1, 126.8, 123.2, 116.1, 38.0, 21.3. HRMS (ESI) *m*/*z* [M + H]^+^ calcd. for C_23_H_18_C_l2_NO: 394.0760, found: 394.0758.

*2,3-Bis(4-chlorophenyl)-6-methoxycarbonyl-1-methylquinolin-4(1H)-one* (**4n**). White solid (74%). m.p. 278.4–279.1 °C; ^1^H NMR (500 MHz, Chloroform-*d*) δ 9.17 (d, *J* = 2.1 Hz, 1H), 8.35 (dd, *J* = 9.0, 2.2 Hz, 1H), 7.59 (d, *J* = 9.0 Hz, 1H), 7.36–7.29 (m, 2H), 7.16–7.07 (m, 4H), 6.99–6.91 (m, 2H), 3.97 (s, 3H), 3.55 (s, 3H); ^13^C NMR (125 MHz, Chloroform-*d*) δ 176.3, 166.8, 151.6, 144.4, 135.9, 133.8, 133.3, 133.1, 133.0, 132.9, 131.2, 130.3, 129.5, 128.4, 126.3, 125.9, 124.6, 116.5, 52.7, 38.4. HRMS (ESI) *m*/*z* [M + H]^+^ calcd. for C_24_H_18_C_l2_NO_3_: 438.0658, found: 438.0661.

*1-Methyl-2,3-diphenylquinoline-4(1H)-thione* (**5**). Brown solid (95%). ^1^H NMR (400 MHz, DMSO-*d*_6_) δ 9.02 (dd, *J* = 8.3, 1.5 Hz, 1H), 8.00 (d, *J* = 8.7 Hz, 1H), 7.89 (ddd, *J* = 8.6, 7.0, 1.6 Hz, 1H), 7.63–7.58 (m, 1H), 7.33–7.23 (m, 5H), 7.11–7.06 (m, 2H), 7.02–6.98 (m, 1H), 6.96–6.91 (m, 2H), 3.61 (s, 3H). ^13^C NMR (125 MHz, DMSO-*d*_6_) δ 140.8, 137.3, 136.9, 135.2, 133.8, 132.9, 131.5, 130.7, 129.6, 129.0, 128.5, 127.6, 126.1, 126.0, 118.7, 39.2. HRMS (ESI) *m*/*z* [M + H]^+^ calcd. for C_22_H_17_NS: 327.1082, found: 327.1072.

*4-((2-Ethoxy-2-oxoethyl)thio)-1-methyl-2,3-diphenylquinolin-1-ium bromide* (**6**). White solid (84%). ^1^H NMR (400 MHz, DMSO-*d*_6_) δ 8.97 (d, *J* = 8.5 Hz, 1H), 8.70 (d, *J* = 9.0 Hz, 1H), 8.38 (t, *J* = 8.0 Hz, 1H), 8.20 (t, *J* = 7.8 Hz, 1H), 7.40 (s, 5H), 7.27 (d, *J* = 5.6 Hz, 3H), 7.17–7.10 (m, 2H), 4.25 (s, 3H), 3.95 (q, *J* = 7.1 Hz, 2H), 3.54 (s, 2H), 1.05 (t, *J* = 7.1 Hz, 3H). ^13^C NMR (125 MHz, DMSO-*d*_6_) δ 168.0, 158.3, 155.2, 140.3, 138.3, 136.1, 135.7, 132.9, 131.0, 130.6, 130.5, 129.6, 129.1, 128.8, 128.6, 128.5, 121.1, 61.8, 43.7, 37.2, 14.2. HRMS (ESI) *m*/*z* [M + H]^+^ calcd. for C_26_H_24_NO_2_S^+^: 414.1522, found: 414.1512.

## 4. Conclusions

In summary, we have developed a traceless approach to quinolin-4(1*H*)-one derivatives through rhodium(III)-catalyzed C−H annulation of *N*-nitrosoanilines with cyclopropenones. This reaction system provides a straightforward and atom-economical route for constructing the six-membered quinolin-4(1*H*)-one scaffolds, which may find important synthetic applications in the construction of heterocyclic compounds in pharmaceutical chemistry.

## Figures and Tables

**Table 1:**
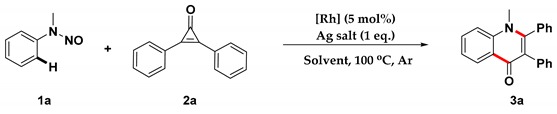
Optimization of Reaction Conditions ^a^.

Entry	[Rh]	[Ag]	Solvent	Yield (%) ^b^
1	[Cp*RhCl_2_]_2_	AgBF_4_	DCE	13
2	Rh(PPh_3_)_3_Cl	AgBF_4_	DCE	0
3	Rh(COD)_2_(BF_4_)	AgBF_4_	DCE	0
4	/	AgBF_4_	DCE	0
5 ^c^	[Cp*RhCl_2_]_2_	AgBF_4_	DCE	49
6 ^d^	[Cp*RhCl_2_]_2_	AgBF_4_	DCE	72 (70) ^j^
7 ^d^	[Cp*RhCl_2_]_2_	AgSbF_6_	DCE	40
8 ^d^	[Cp*RhCl_2_]_2_	AgOTf	DCE	23
9 ^d^	[Cp*RhCl_2_]_2_	/	DCE	0
10 ^d^	[Cp*RhCl_2_]_2_	AgBF_4_	THF	69
11 ^d^	[Cp*RhCl_2_]_2_	AgBF_4_	Acetone	66
12 ^d,e^	[Cp*RhCl_2_]_2_	AgBF_4_	DCE	49
13 ^d,f^	[Cp*RhCl_2_]_2_	AgBF_4_	DCE	49
14 ^d,g^	[Cp*RhCl_2_]_2_	AgBF_4_	DCE	67
15 ^d,h^	[Cp*RhCl_2_]_2_	AgBF_4_	DCE	57
16 ^d,i^	[Cp*RhCl_2_]_2_	AgBF_4_	DCE	0

^a^ Reaction conditions: **1a** (0.2 mmol), **2a** (0.2 mmol), Ag salt (0.2 mmol), solvent (2 mL), sealed tube under argon, 2 h. ^b^ NMR yield using CH_2_Br_2_ as internal standard. ^c^ solvent (5 mL). ^d^ solvent (10 mL). ^e^ HOAc (20 mol%) was added. ^f^ CsF (20 mol%) was added. ^g^ Zn(OAc)_2_ (20 mol%) was added. ^h^ at 80 °C. ^i^ at 60 °C. ^j^ isolated yield. DCE: dichloroethane. THF: tetrahydrofuran.

## Supplementary Materials

The following are available online: Figures S1 and S2, Control Experiments for the Mechanistic Studies; Figure S3, X-ray Crystallographic Data; Figure S4, Copies of the ^1^H-NMR, ^13^C-NMR, and ^19^F-NMRspectra.
