# Supplementary material for: Rhodium(III)-Catalyzed Redox-Neutral [3+3] Annulation of N-nitrosoanilines with Cyclopropenones: A Traceless Approach to Quinolin-4(1H)-One Scaffolds
Source: Molecules. 2020 Jan 9;25(2):268. doi: 10.3390/molecules25020268 (PMC7024356; doi:10.3390/molecules25020268)

Supplementary Materials

Rhodium(III)-catalyzed Redox-neutral [3+3] Annulation of *N*-Nitrosoanilines with Cyclopropenones: a Traceless Approach to Quinolin-4(1*H*)-one Scaffolds

Lingjun Liu [1,2], Jiyuan Li [2], Wenhao Dai [1,2], Feng Gao [2], Kaixian Chen*[,1,2,3], Yu Zhou *[,2] and Hong Liu *[,1,2,3]

[1] State Key Laboratory of Natural Medicines and Department of Medicinal Chemistry, China Pharmaceutical University, 24 Tong Jia Xiang, Nanjing, Jiangsu 210009, P. R. China.

[2] State Key Laboratory of Drug Research and Key Laboratory of Receptor Research, Shanghai Institute of Materia Medica, Chinese Academy of Sciences, 555 Zu Chong Zhi Road, Shanghai 201203, China.

[3] Open Studio for Druggability Research of Marine Natural Products, Pilot National Laboratory for Marine Science and Technology (Qingdao), 1 Wenhai Road, Aoshanwei, Jimo, Qingdao, 266237, China.

* Correspondence: kxchen@simm.ac.cn (K.C.); zhouyu@simm.ac.cn (Y.Z.); hliu@simm.ac.cn (H.L)

Table of Contents

1. Figure S1-S2: Mechanistic investigation ···1

2. Figure S3:X-ray Crystallographic Data ···2

3. Figure S4: Copies of ^{1}H-NMR, ^{13}C-NMR and ^{19}F-NMRspectra …………..… 2

1. Control Experiments for the Mechanistic Studies

(a) H/D exchange of *N-nitrosoanilines* (1a) under CH_3OD/Rh(III) catalytical system.

To an oven-dried sealed tube charged with *N*-nitrosoaniline (**1a**) (27.3 mg, 0.20 mmol) and $[Cp^*RhCl_2]_2$ (6.2 mg, 5 mol %), CH_3OD (0.1 mL) and $AgBF_4$ (39.0 mg, 0.20 mmol), DCE (10 mL) was added under argon atmosphere. The reaction mixture was then allowed to stir at 100 ℃ for 1 h. The corresponding reaction mixture was filtered through a pad of celite, washed with DCM and concentrated under reduced pressure. The deuterium incorporation d_2-**1a** was determined to be <5% by ^{1}H NMR method (**Figure S1**).

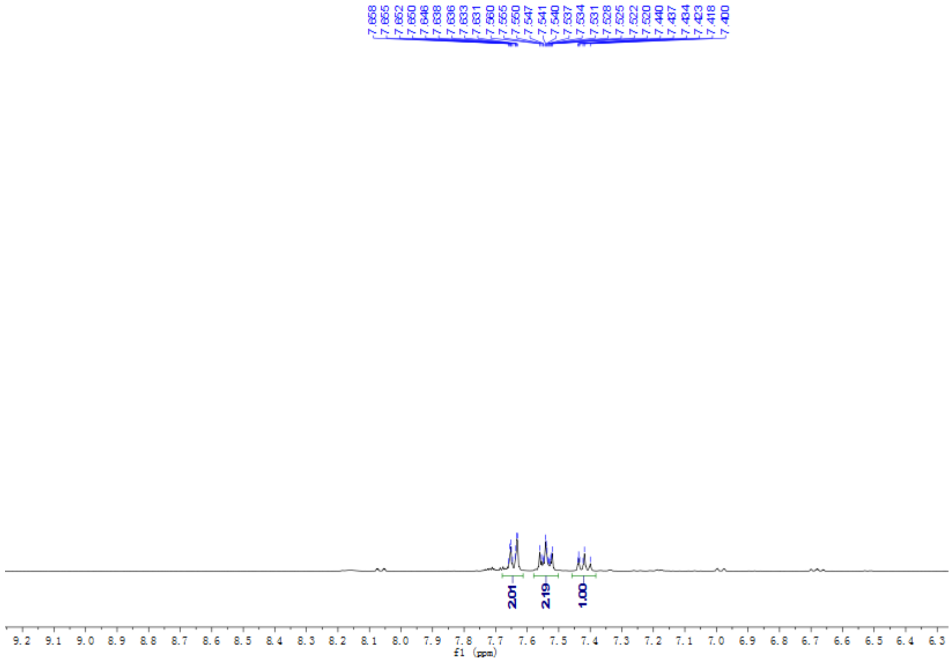

Figure S1. The ^{1}H NMR spectra of d_2-1a

(b) Kinetic isotope effect of the transformation

Compound d_5-**1a** (27.3 mg, 0.20 mmol), **1a** (27.3 mg, 0.20 mmol), 2a (43.6 mg, 0.30 mmol), $[Cp^*RhCl_2]_2$ (6.2 mg, 5 mol %) and $AgBF_4$ (39.0 mg, 0.20 mmol) was combined in a 30 mL dried sealed tube under argon atmosphere. The reaction mixture was magnetically stirred and heated to 100 ℃ for 10 min, then the corresponding reaction mixture was filtered through a pad of celite, washed with DCM and concentrated under reduced pressure. The residue was purified by flash chromatography on silica gel using ethyl acetate/dichloromethane/petroleum ether as eluent to afford the desired products **3a** and d_4-**3a** as yellow solid. The deuterium incorporation was determined to be k_H/k_D = 1.7 by ^{1}H NMR method (**Figure S2**).

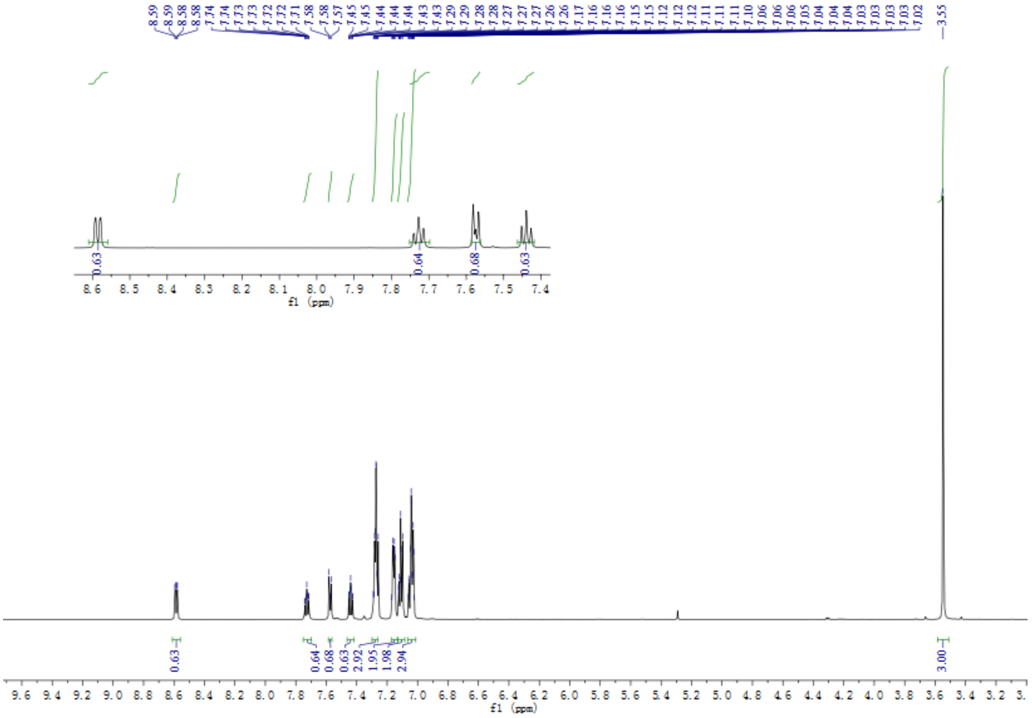

Figure S2. The conversion of 1a and d_5-1a was monitored by ^{1}H-NMR method

(c) Intermolecular competition experiments between 1d and 1k

N-methyl-*N*-(4-methylphenyl)nitrous amide (**1b**) (30.0 mg, 0.20 mmol), *N*-methyl-*N*-(4-(trifluoromethyl)phenyl)nitrous amide (**1e**) (40.8 mg, 0.20 mmol), $[Cp^*RhCl_2]_2$ (6.2 mg, 5 mol %), $AgBF_4$ (39.0 mg, 0.20 mmol), DCE (10 mL) and diphenylcyclopropenone (**2a**) (61.8 mg, 0.30 mmol) were added to a 35 mL Schlenk tube under Ar. The mixture was stirred at 100 ℃ for 30 min. After cooling to ambient temperature, the corresponding reaction mixture was washed with DCM and concentrated under reduced pressure. The residue was purified by flash chromatography on silica gel to afford the final products **3d** and **3k**.

2. X-ray Crystallographic Data

X-ray Single Crystal Structure Analysis of **3a**

X-ray crystallographic data of **3a** was solutions at T = 173 K：$C_{22}H_{17}NO$，*Mr* = 311.36, monoclinic. Space group *P-1*, a = 11.8987 (5) Å, b = 8.1773 (3) Å, c = 16.9235 (8) Å, α = 90°, β = 100.020(2)°, γ = 90 °, *V* = 1621.53 (12) Å^3, *Z* = 4.

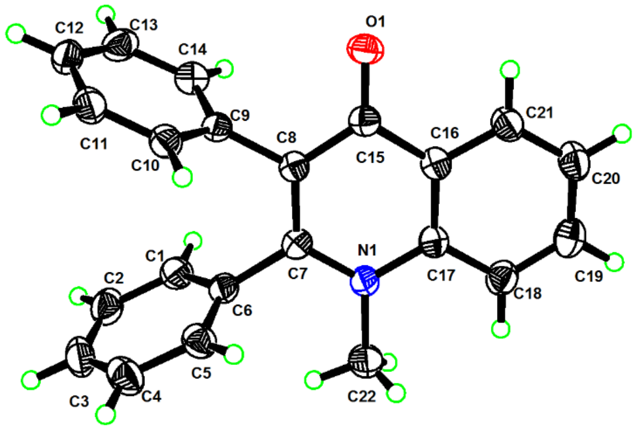

Figure S3: The crystal structure of **3a** by X-ray analysis.

These data can be obtained free of charge from the Cambridge Crystallographic Data Centre via www.ccdc.cam.ac.uk/data_request/cif, the CCDC number is 1968674.

3. ^{1}H-NMR, ^{13}C-NMR and ^{19}F-NMRspectra

(1) The ^{1}H NMR and ^{13}C NMR spectrum for **3a**

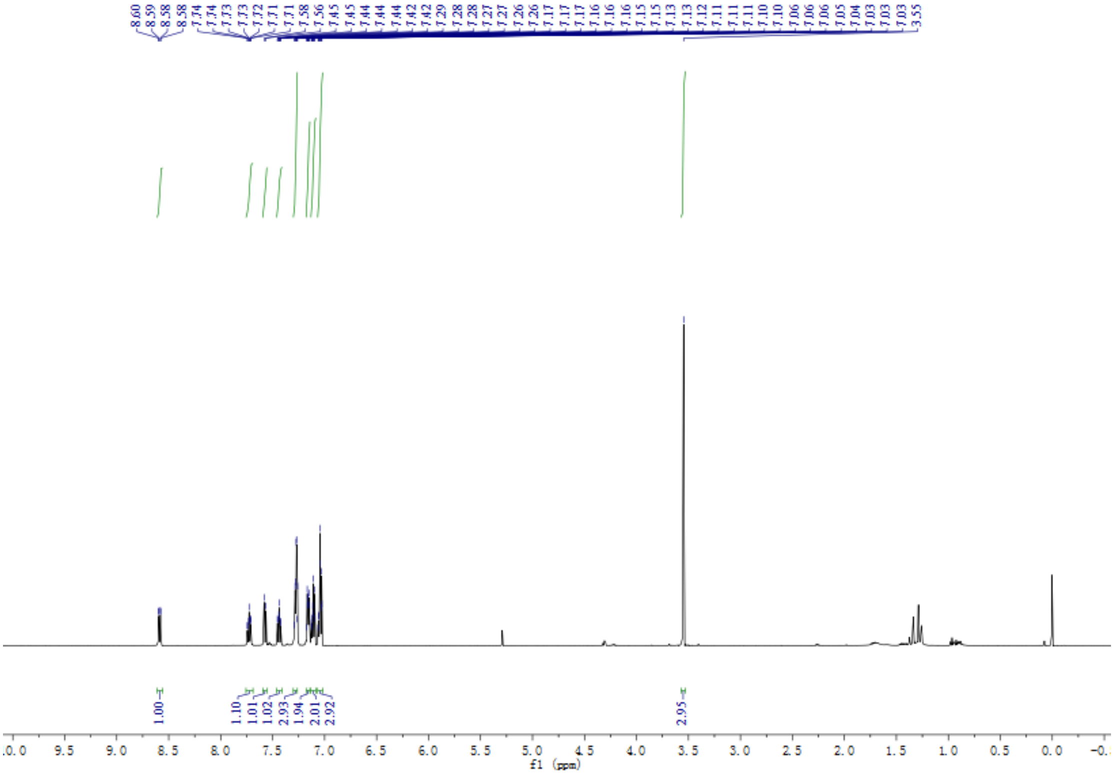

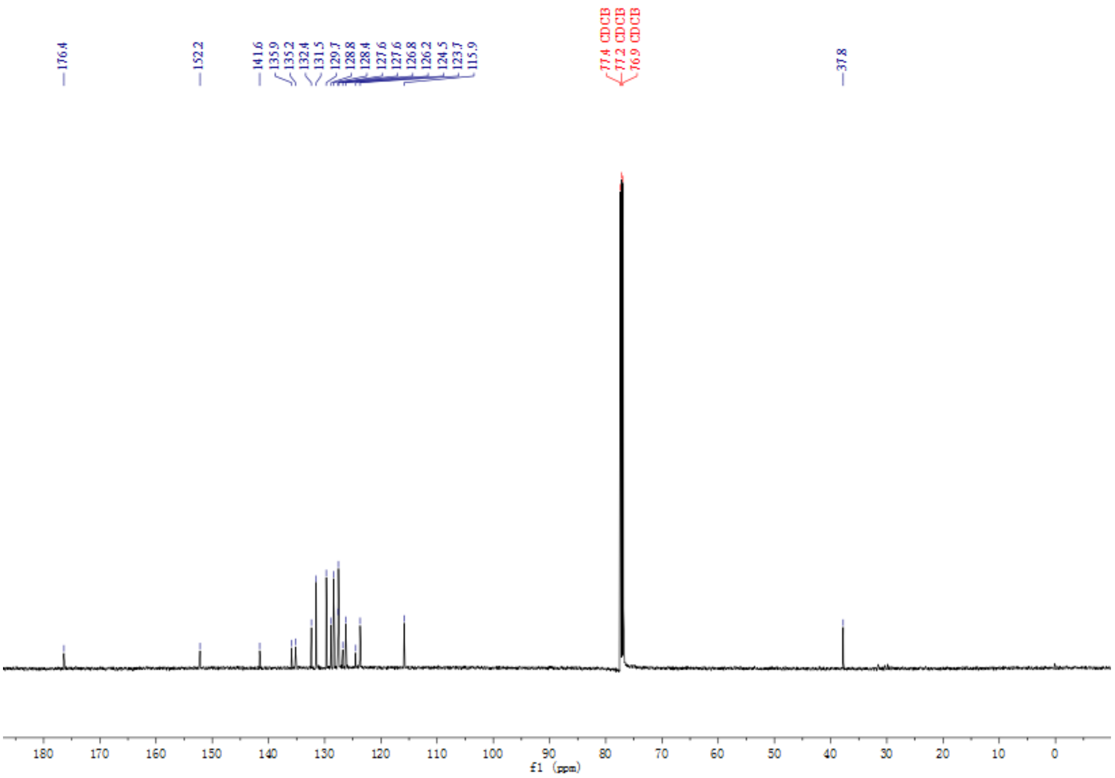

(2) The ^{1}H NMR and ^{13}C NMR spectrum for **3b**

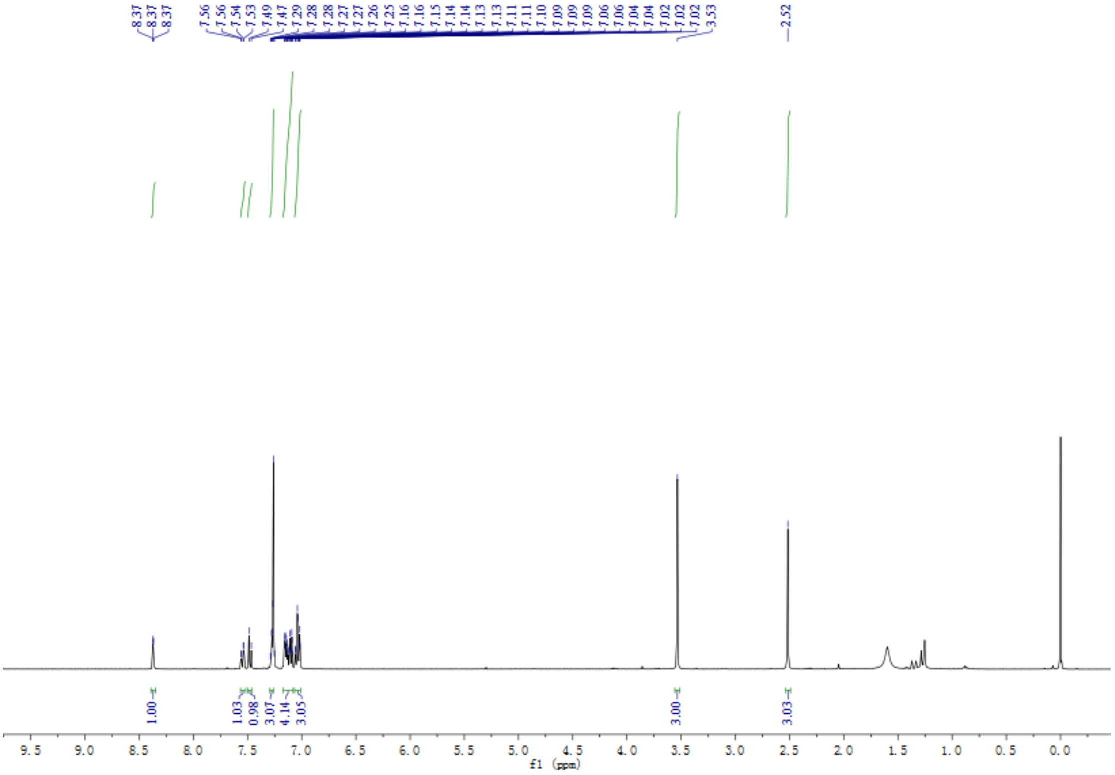

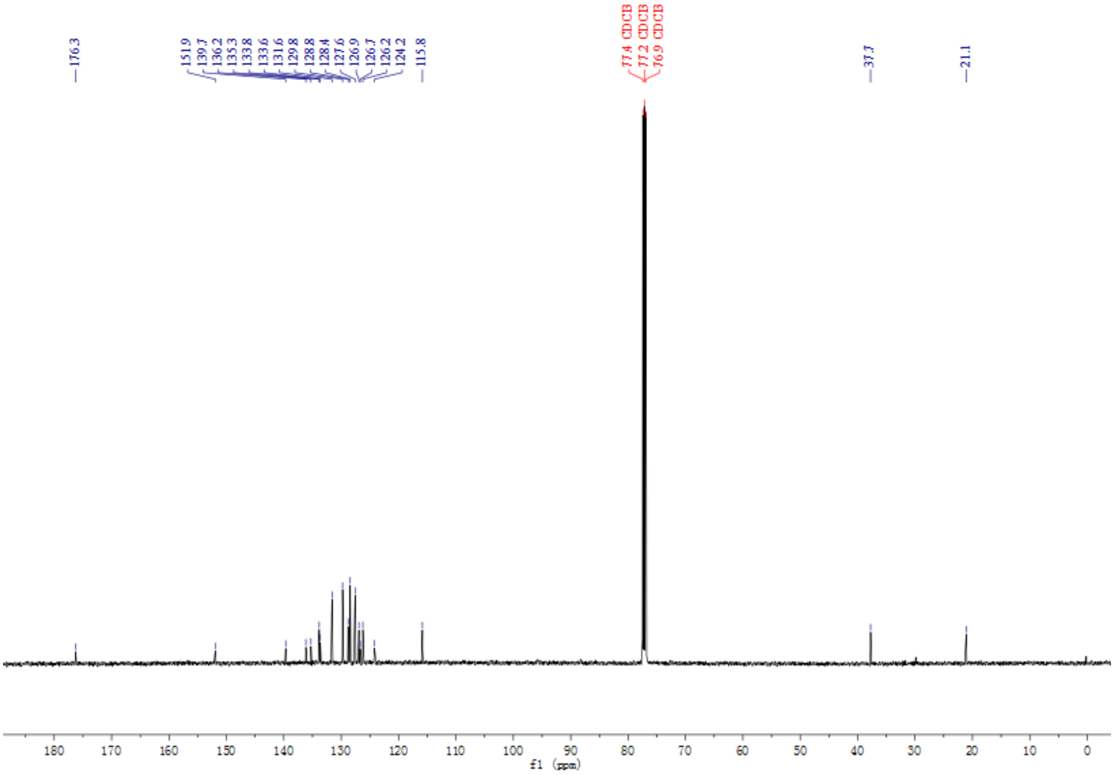

(3) The ^{1}H NMR and ^{13}C NMR spectrum for **3c**

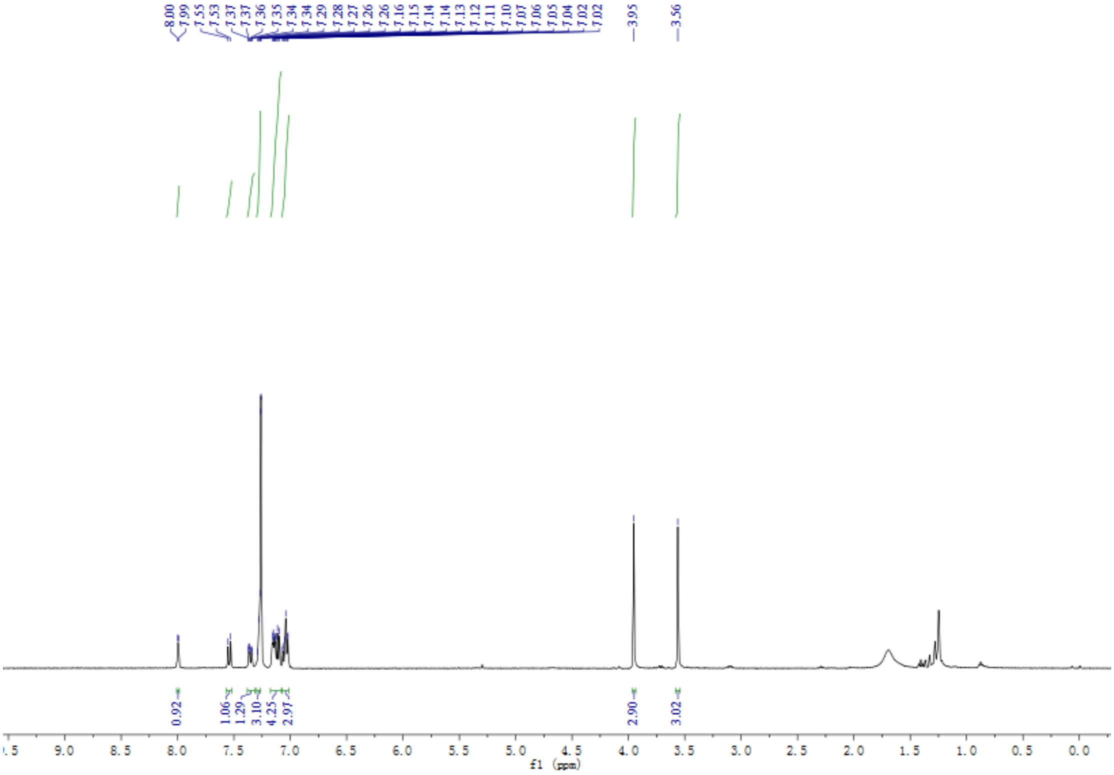

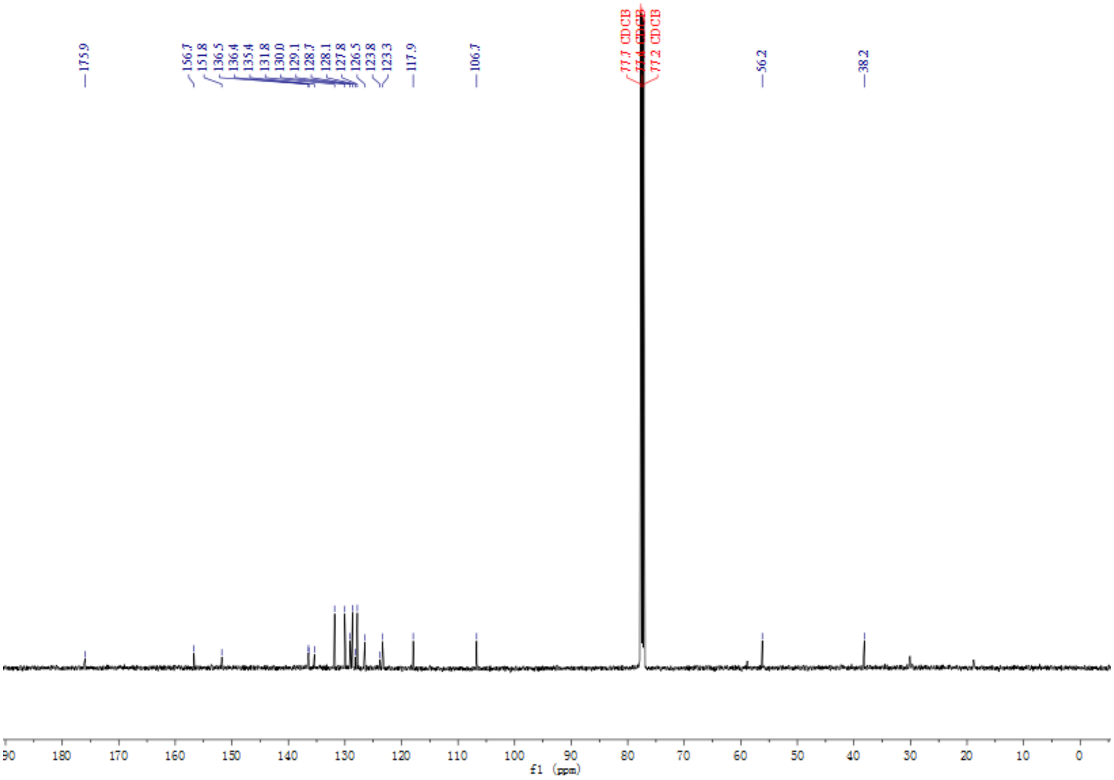

(4) The ^{1}H NMR and ^{13}C NMR spectrum for **3d**

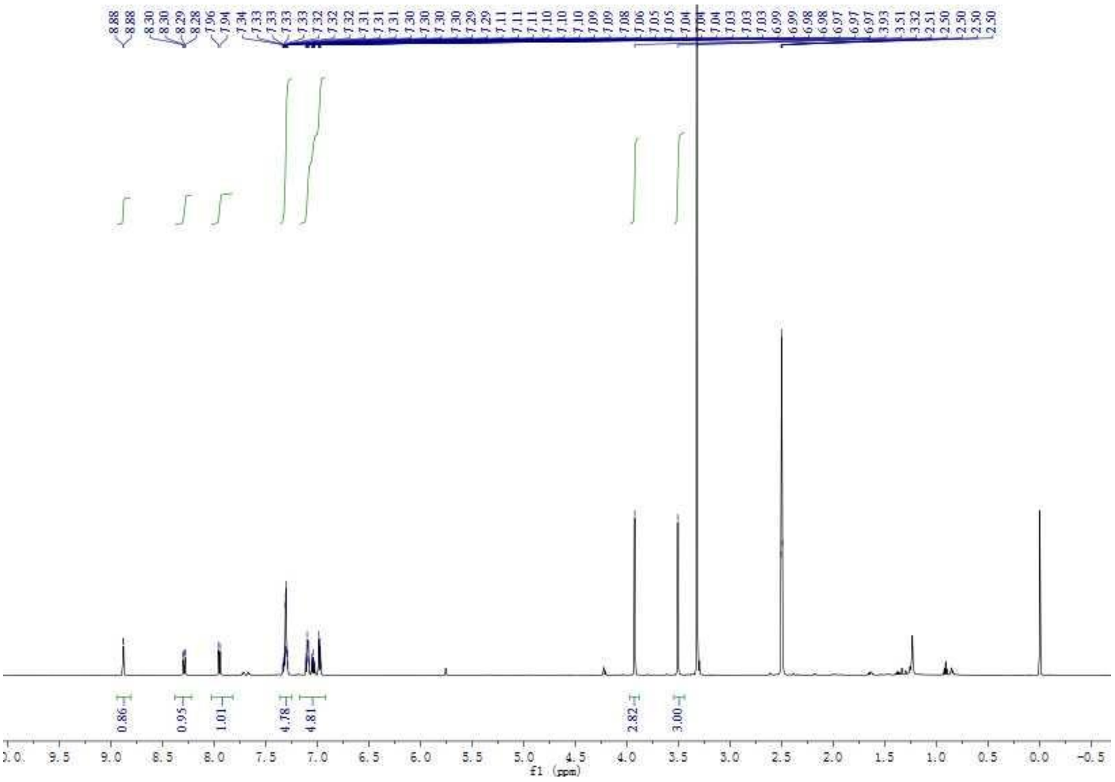

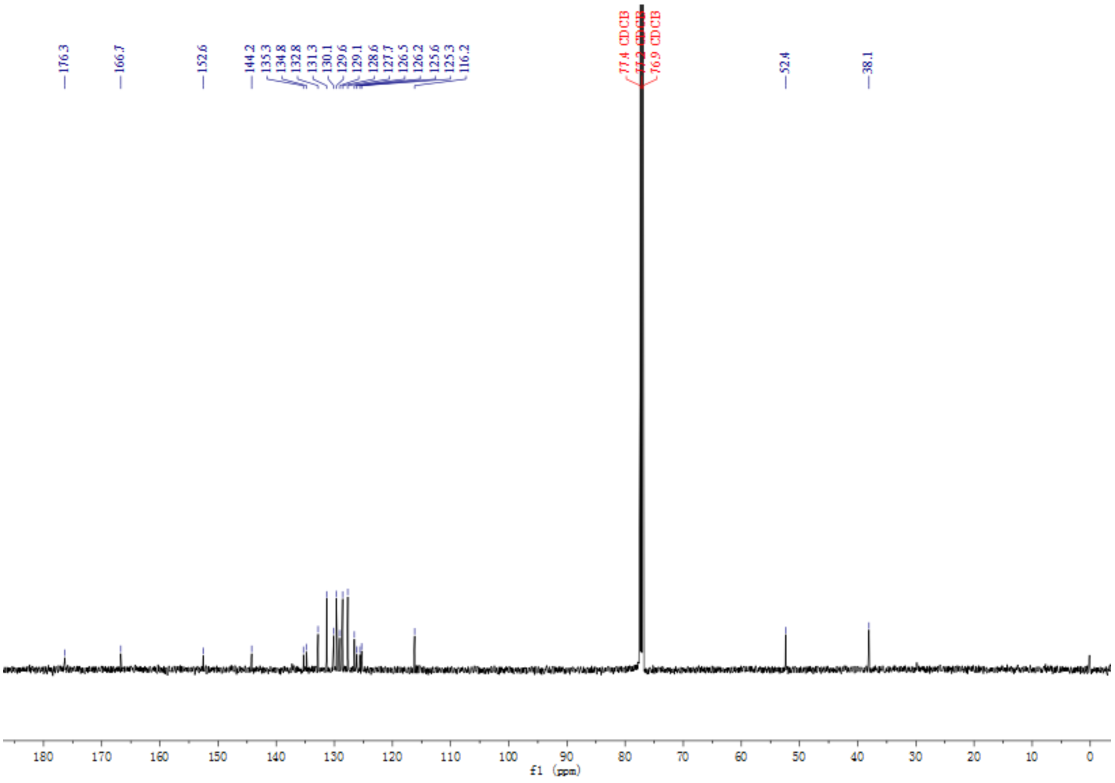

(5) The ^{1}H-NMR, ^{13}C-NMR and ^{19}F-NMRspectra for **3e**

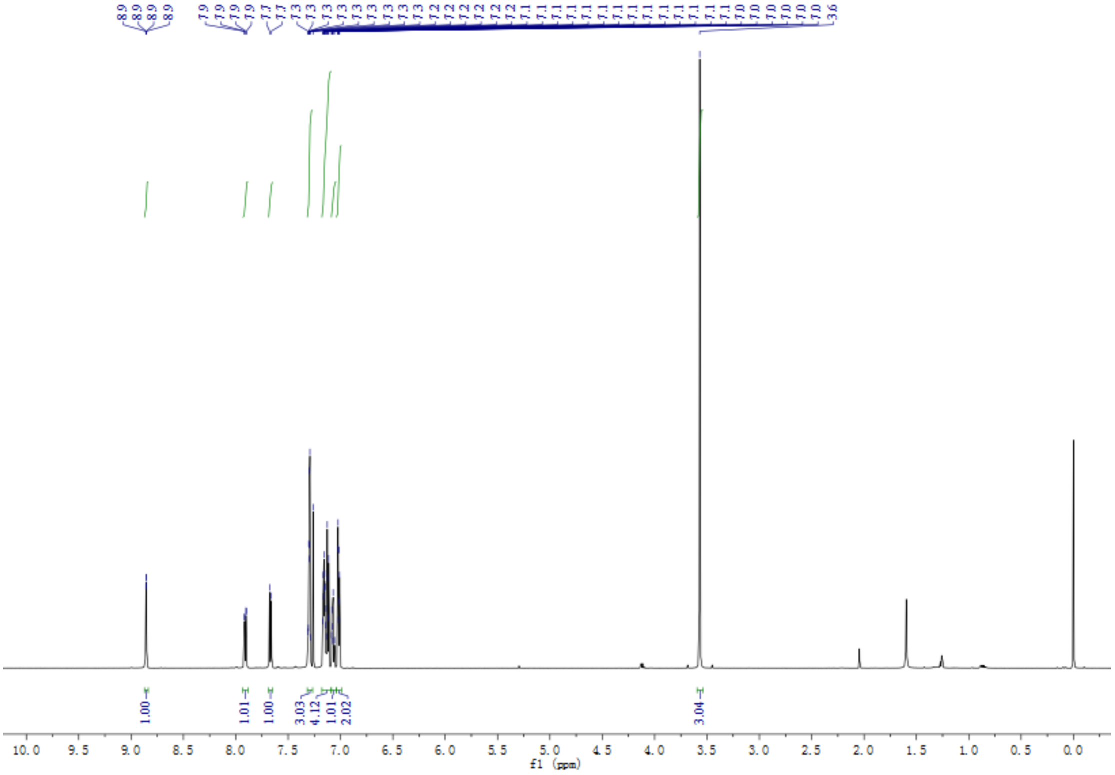

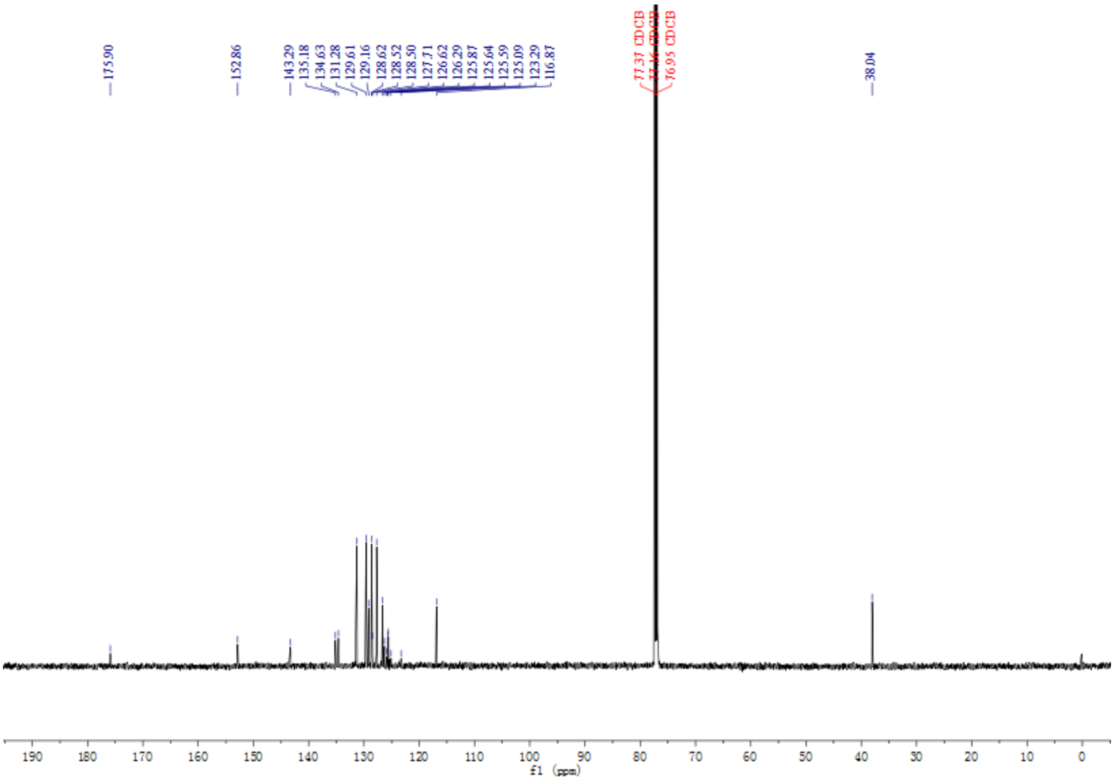

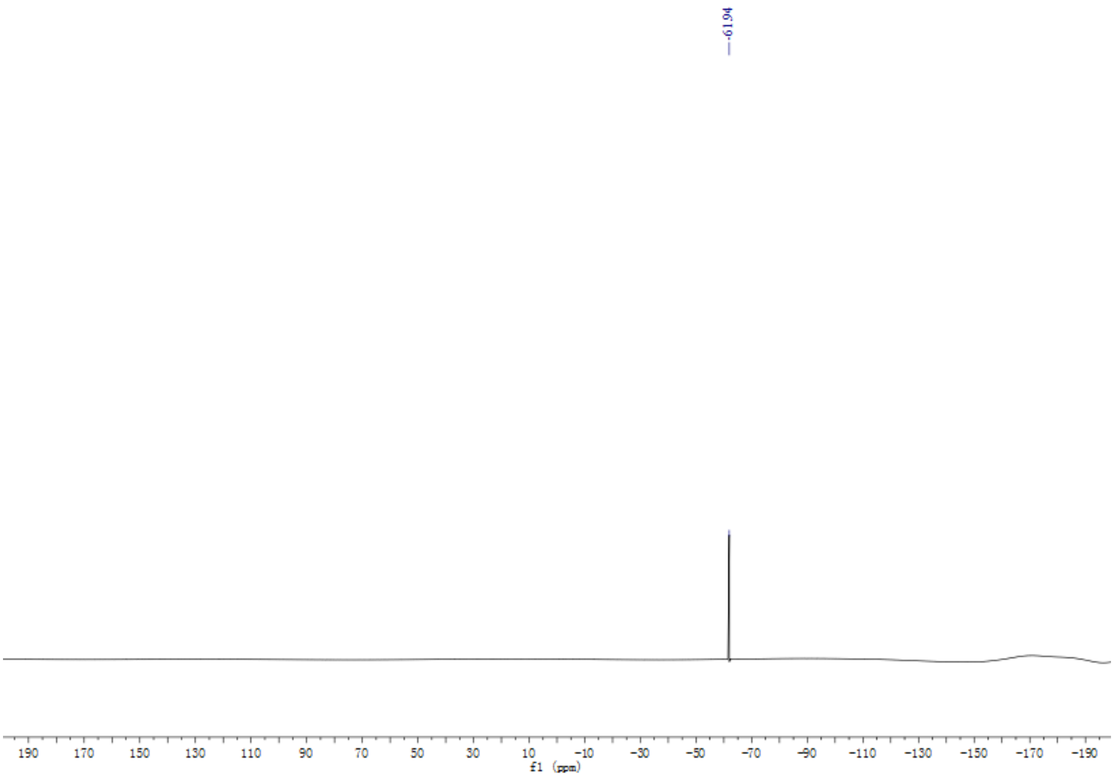

(6) The ^{1}H-NMR, ^{13}C-NMR and ^{19}F-NMRspectra for **3f**

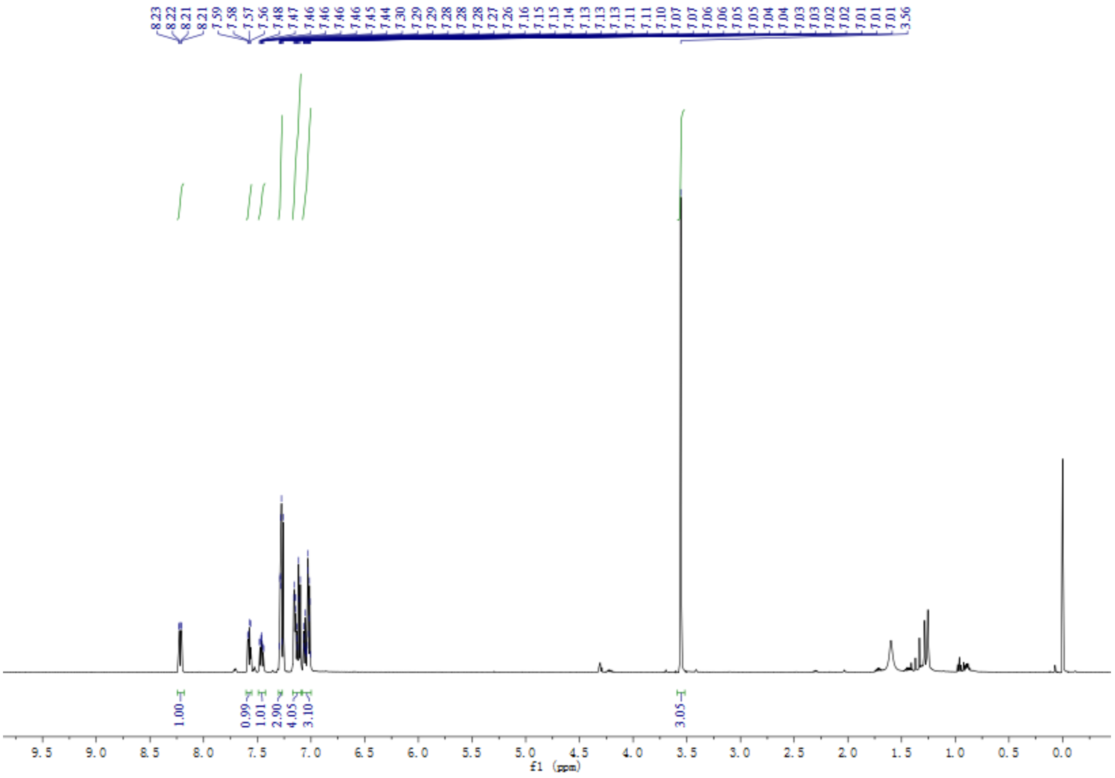

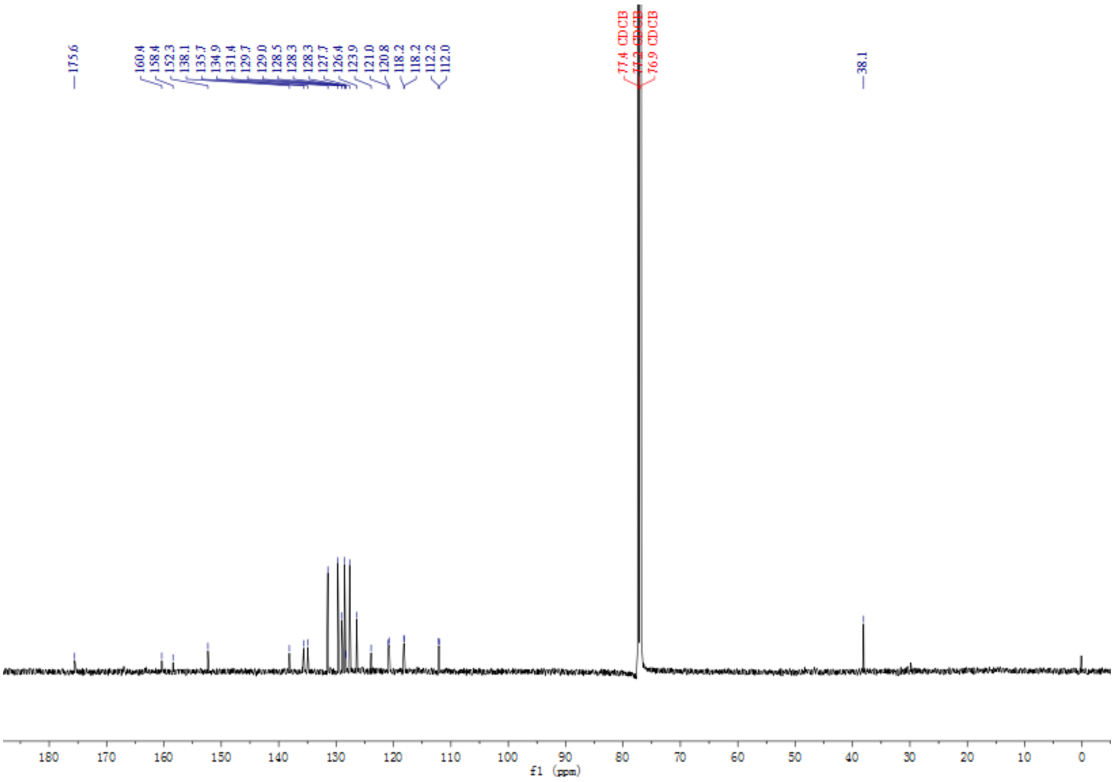
175.6
160.4
158.4
152.3
138.1
135.7
134.9
131.4
129.7
129.7
129.0
128.5
128.3
128.3
127.7
126.4
123.9
121.0
120.8
118.2
118.2
112.2
112.0
77.4 CDCl3
76.9 CDCl3
38.1
f1 (ppm)

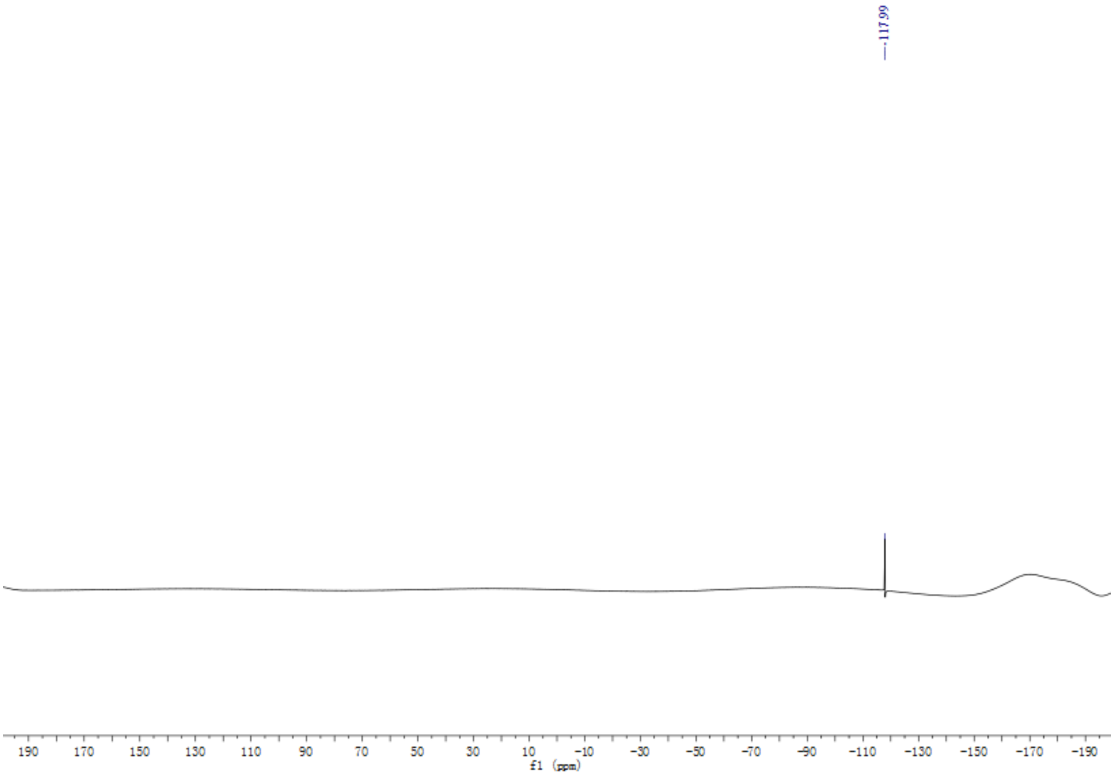
-117.99
f1 (ppm)

(7) The ^{1}H NMR and ^{13}C NMR spectrum for **3g**

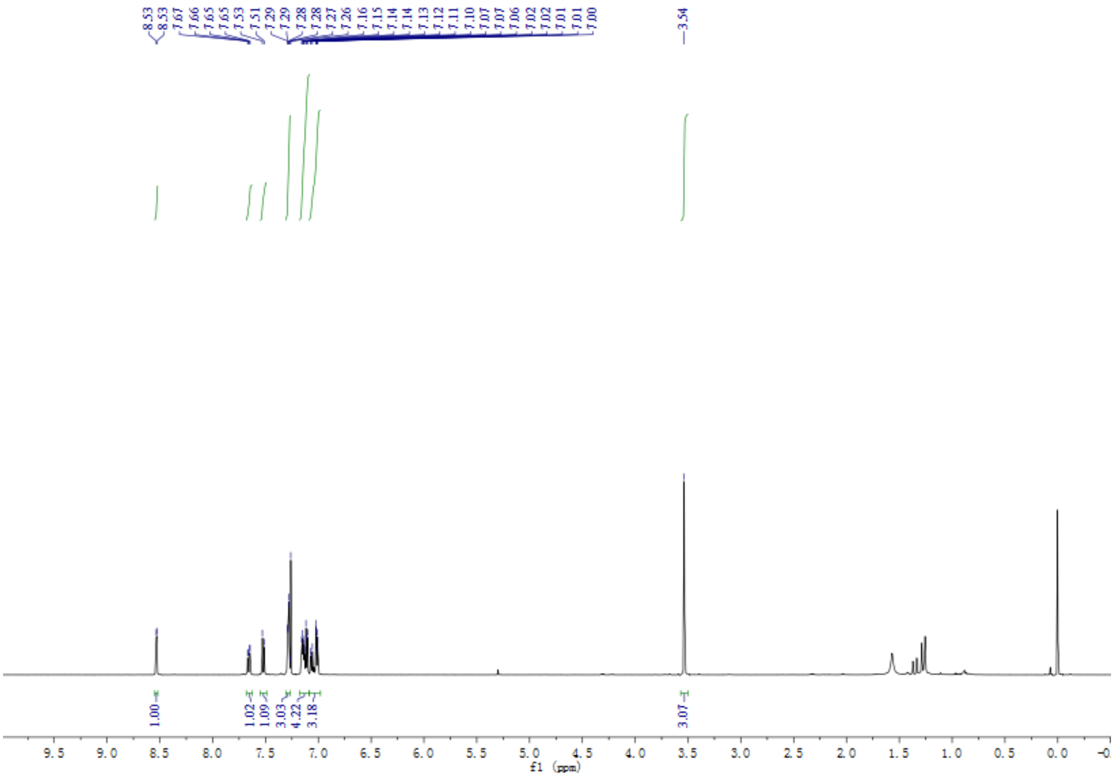

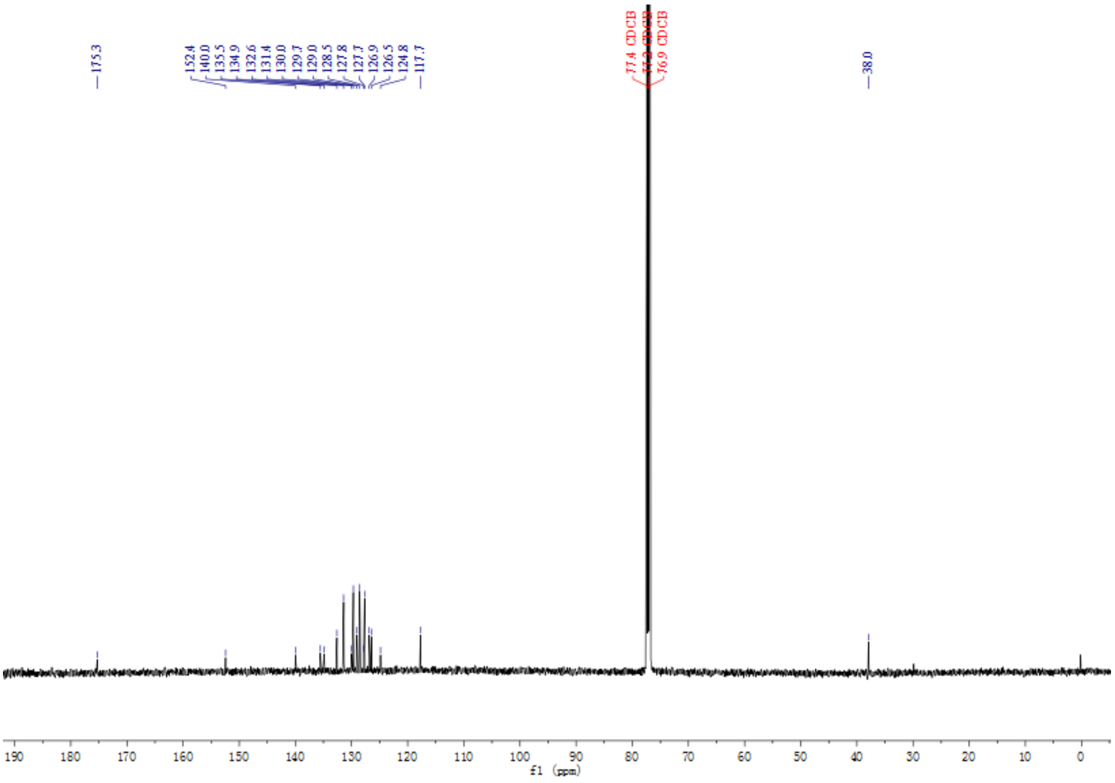

(8) The ^{1}H NMR and ^{13}C NMR spectrum for **3h**

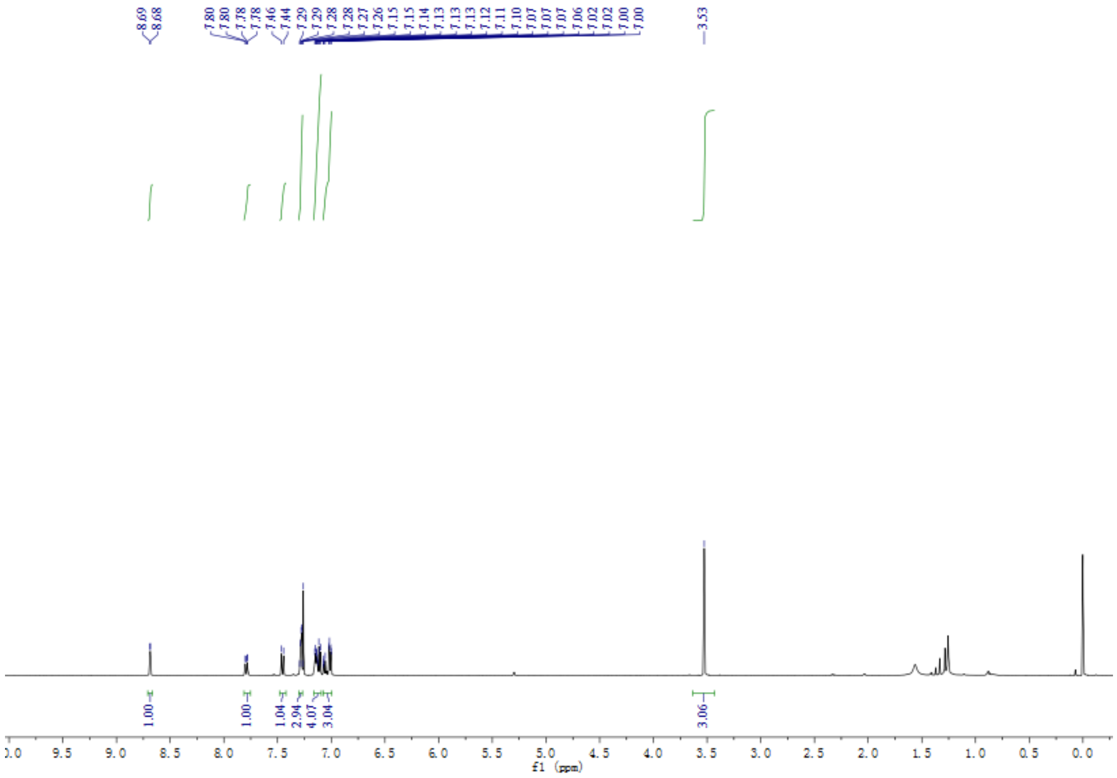

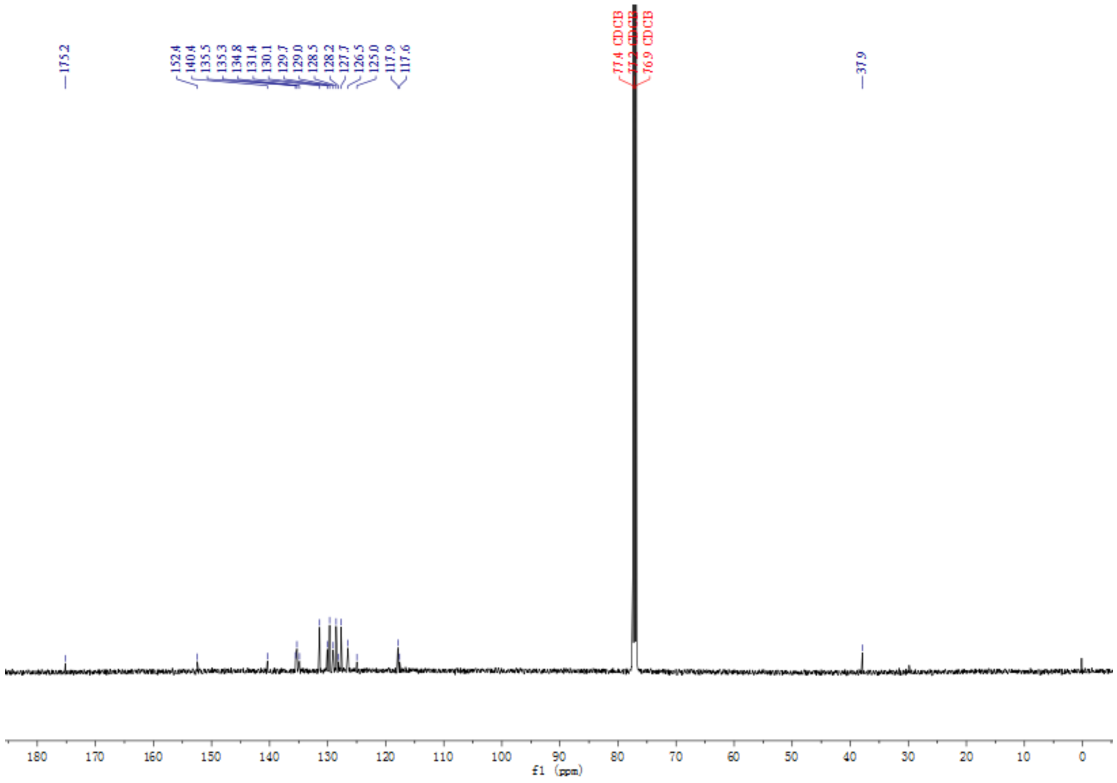

(9) The ^{1}H NMR and ^{13}C NMR spectrum for **3i**

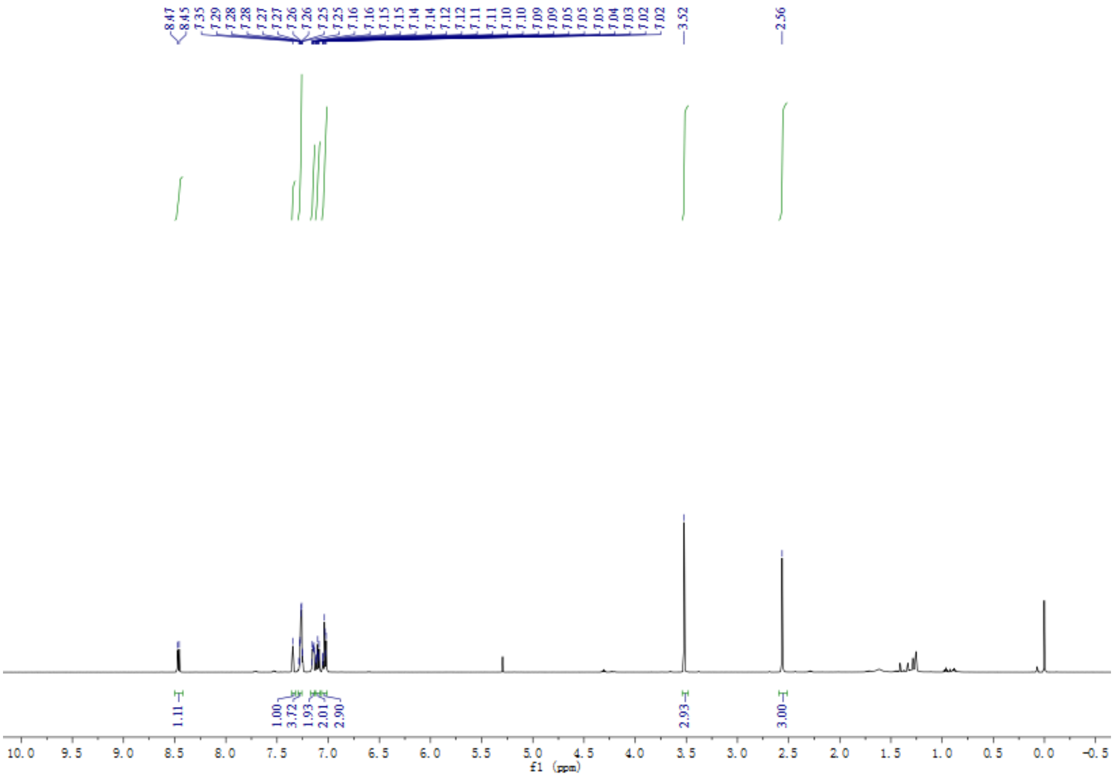

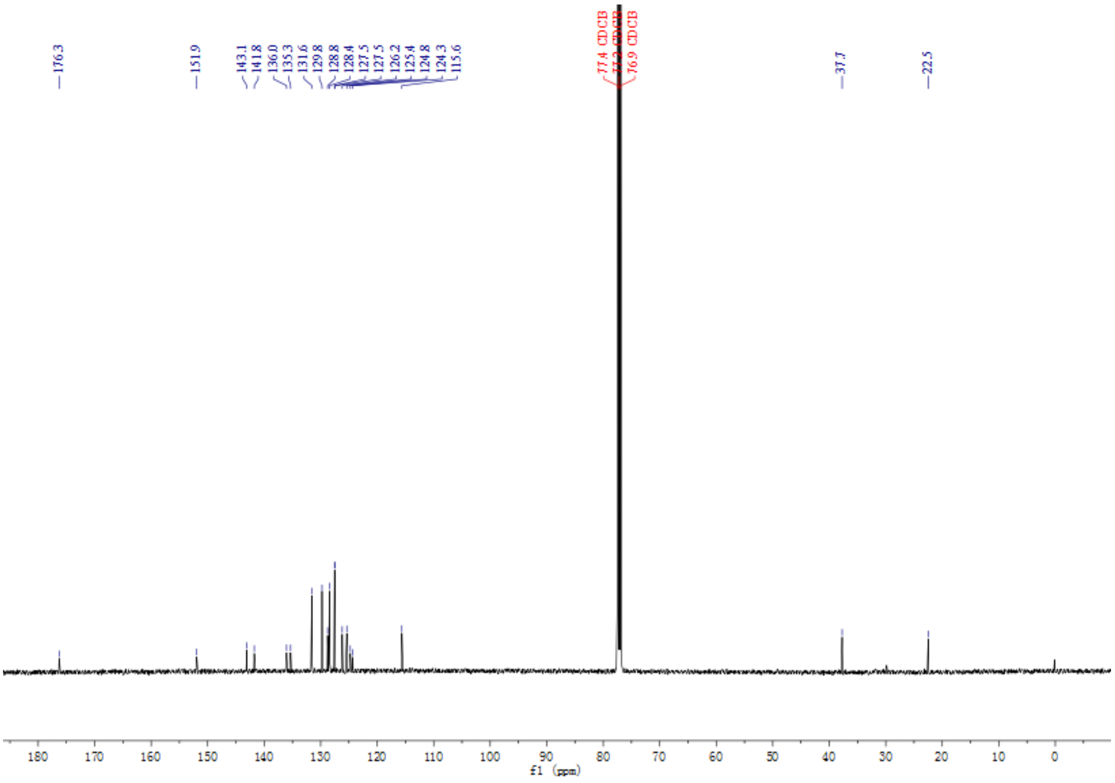

(10) The ^{1}H-NMR, ^{13}C-NMR and ^{19}F-NMRspectra for **3j**

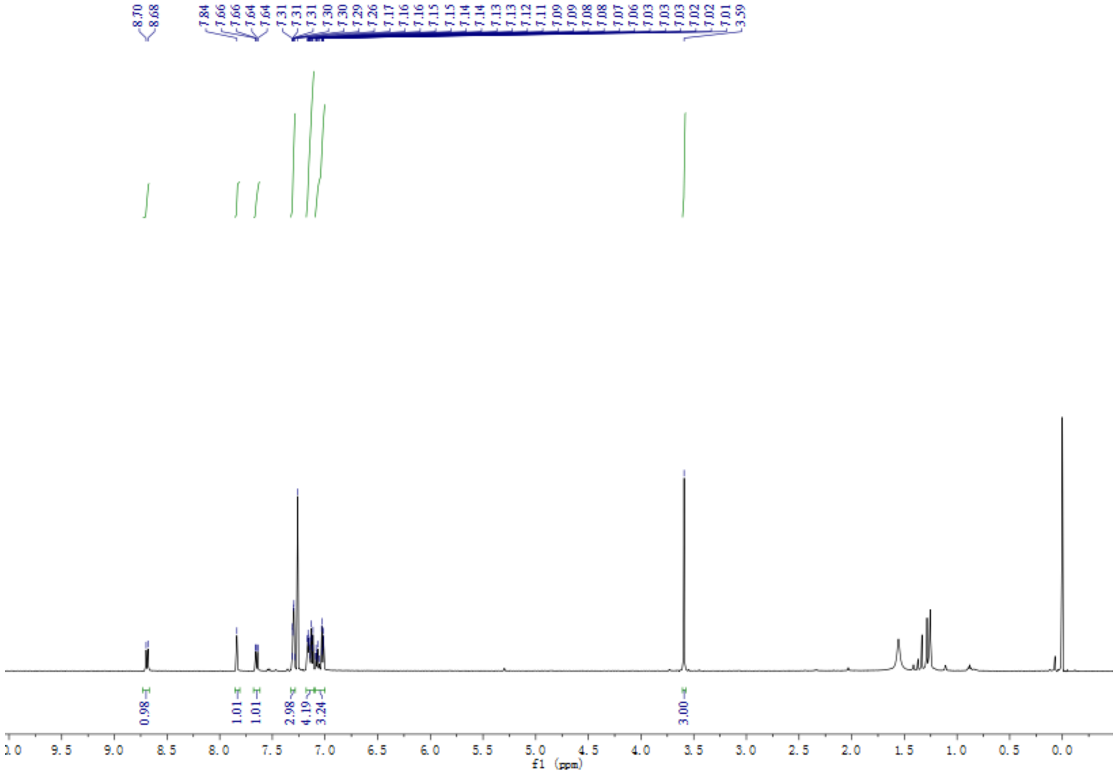

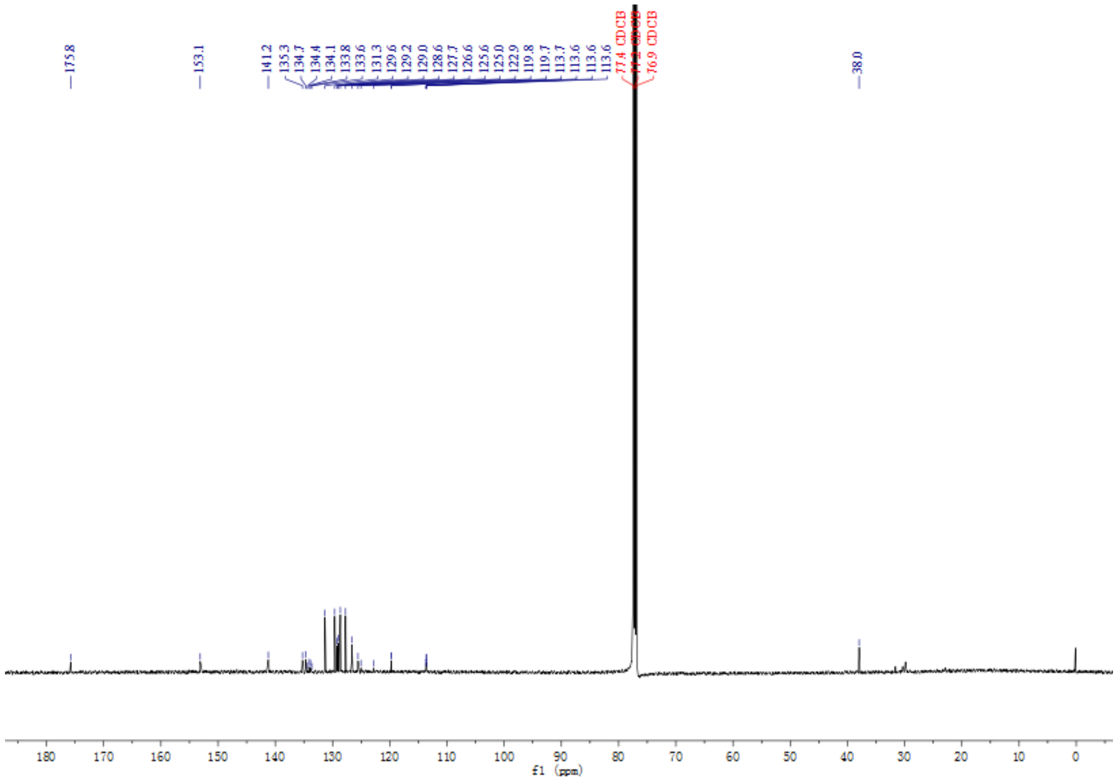

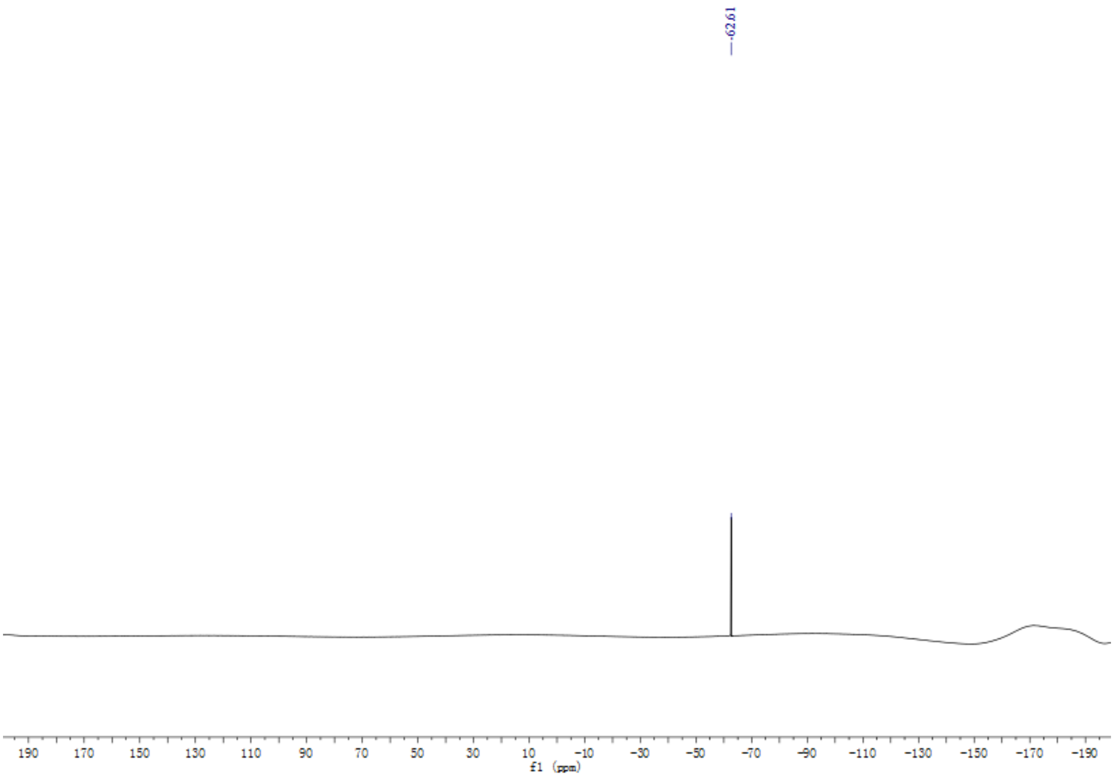

(11) The ^{1}H NMR and ^{13}C NMR spectrum for **3k**

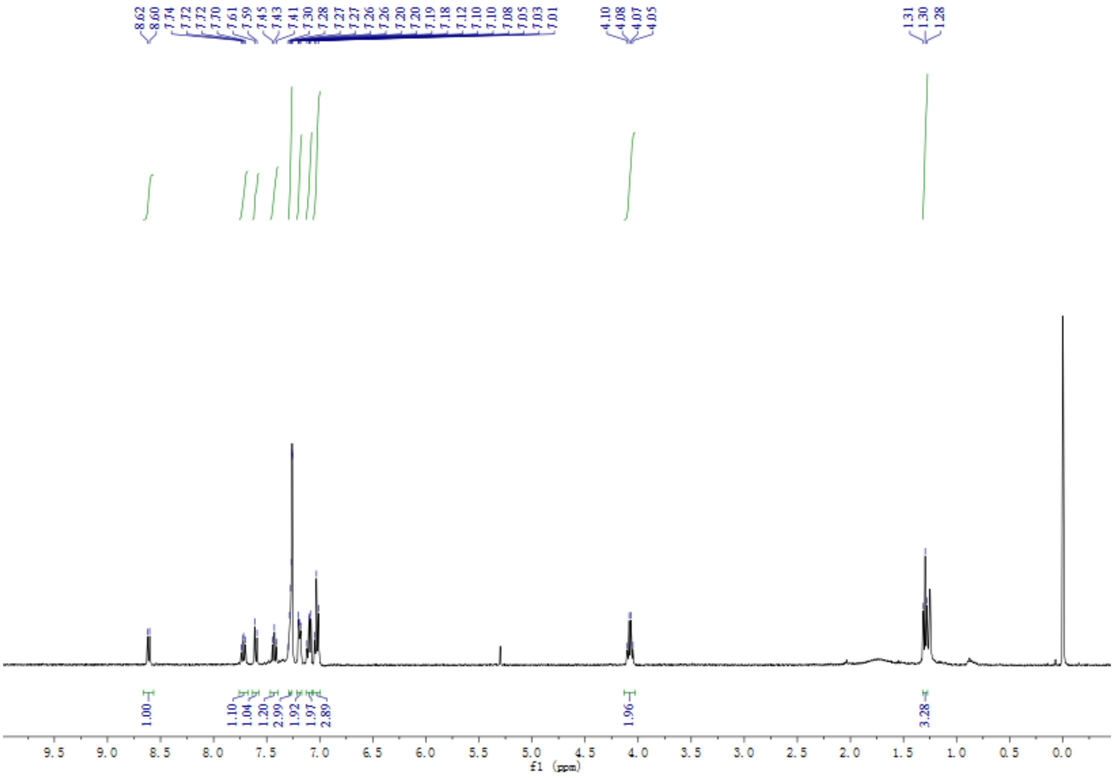

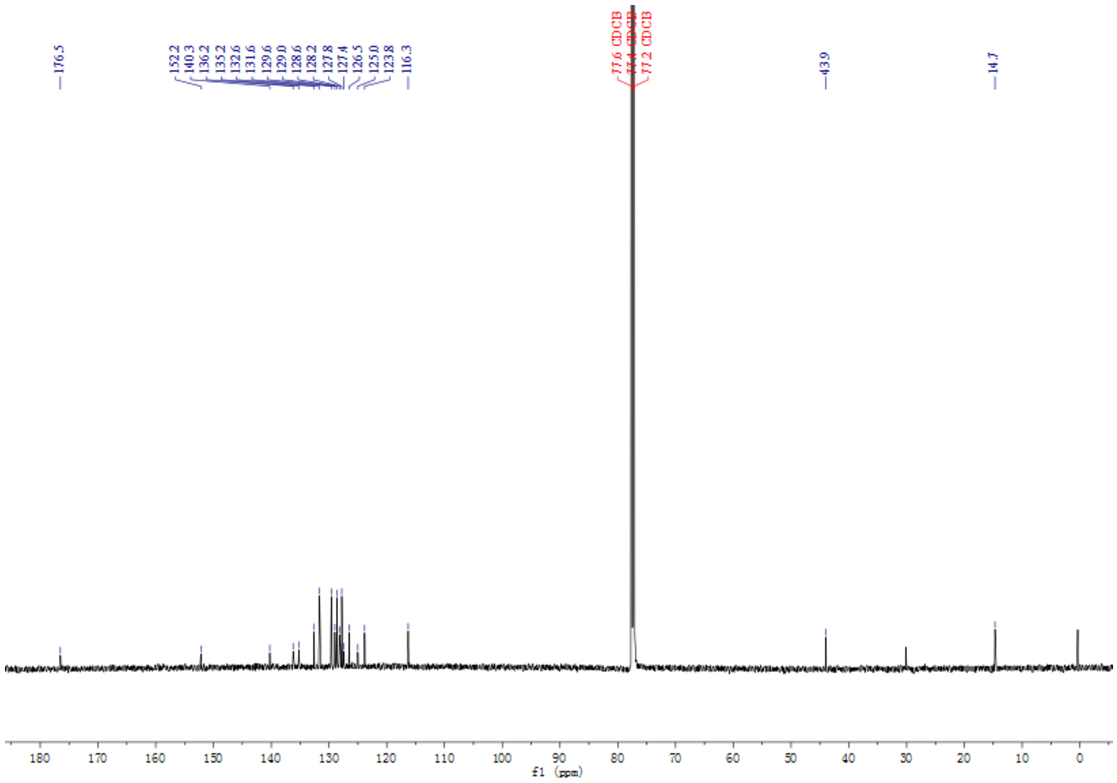

(12) The ^{1}H NMR and ^{13}C NMR spectrum for **3l**

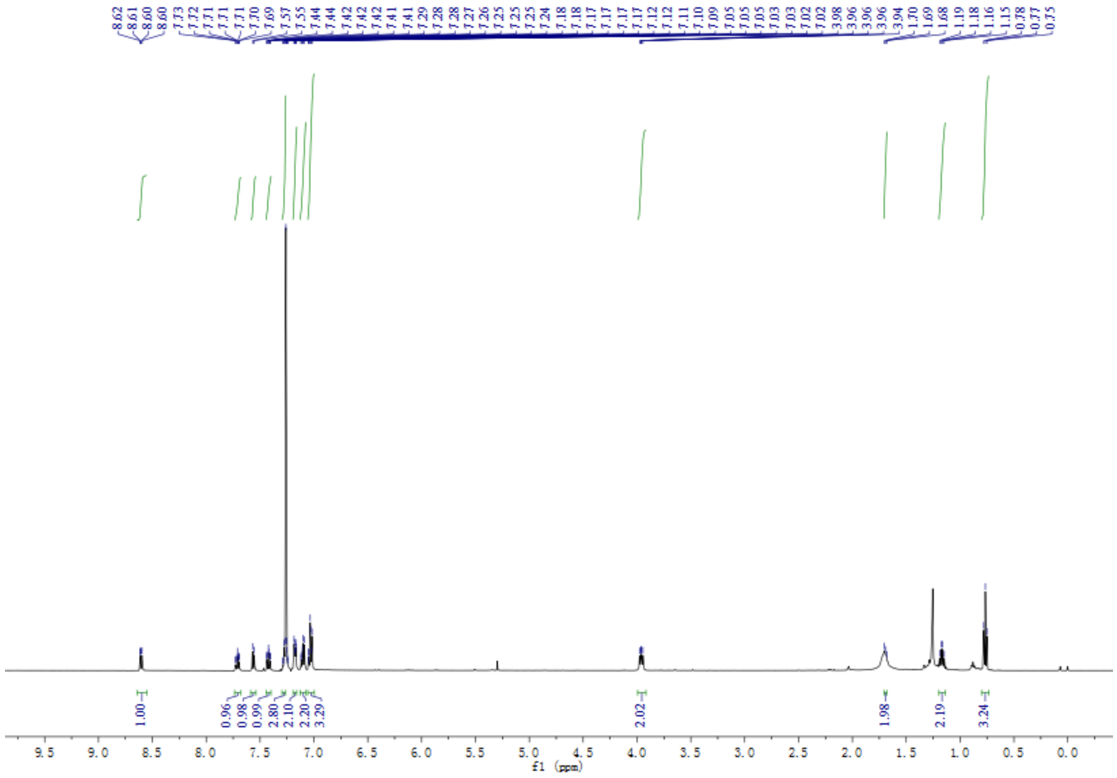

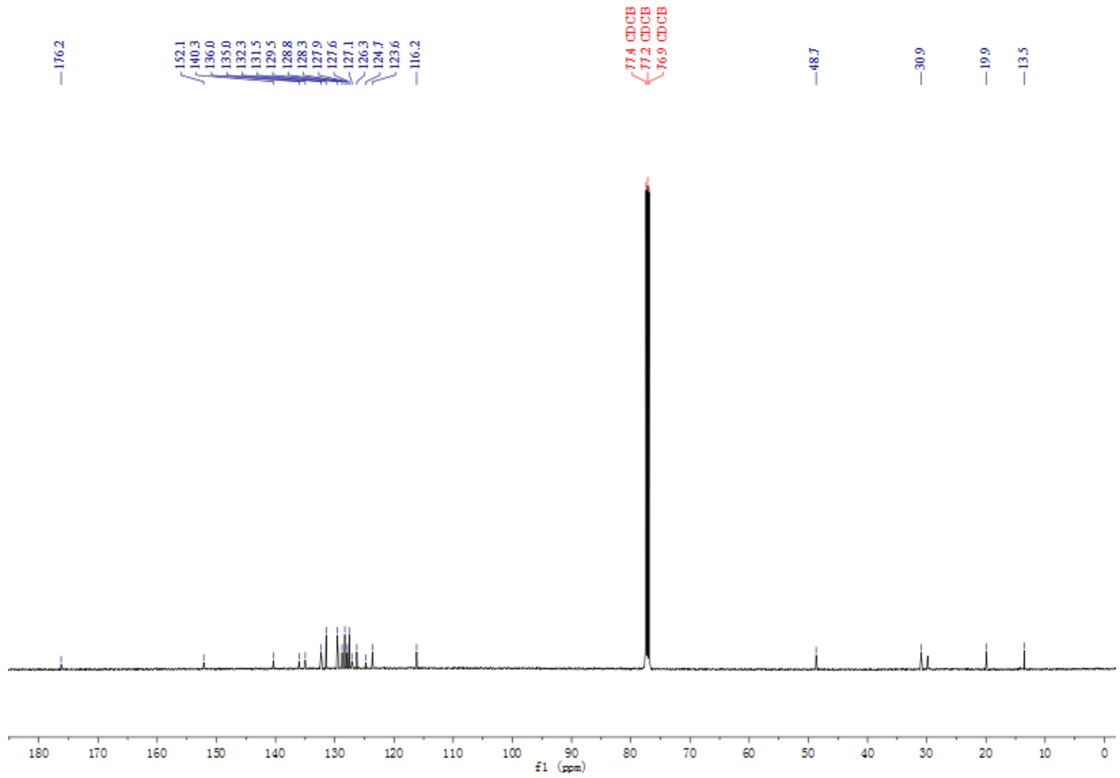

(13) The ^{1}H NMR and ^{13}C NMR spectrum for **3m**

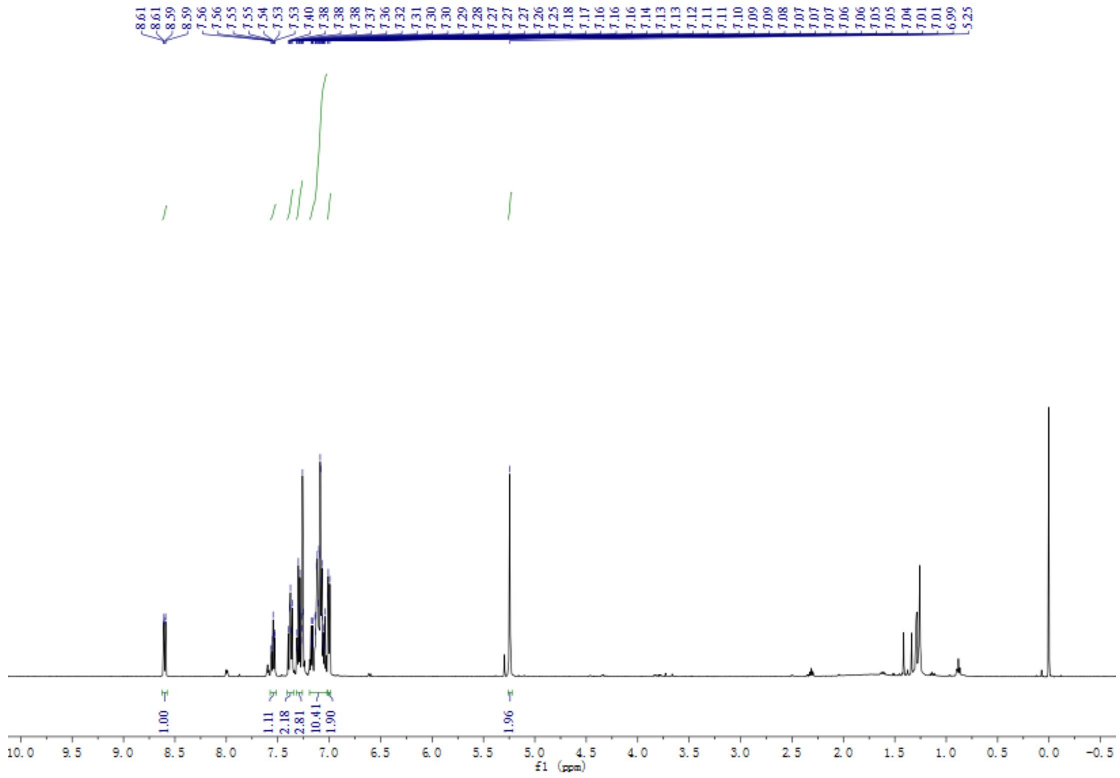

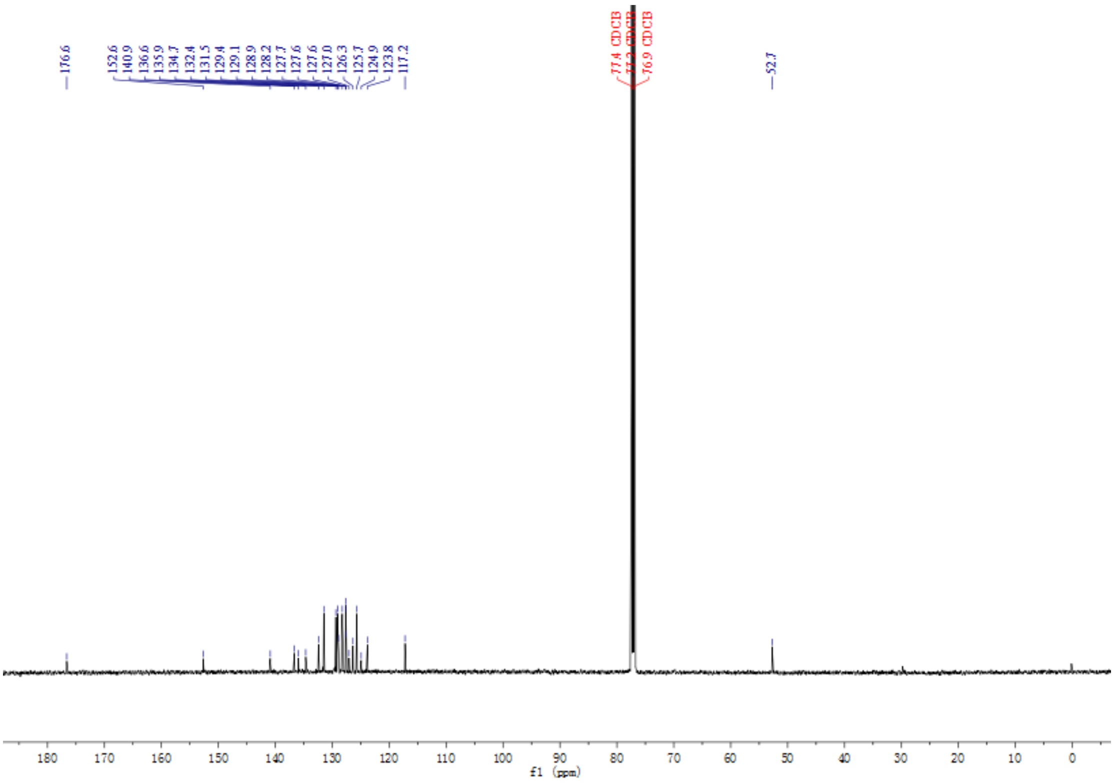

(14) The ^{1}H NMR and ^{13}C NMR spectrum for **3n**

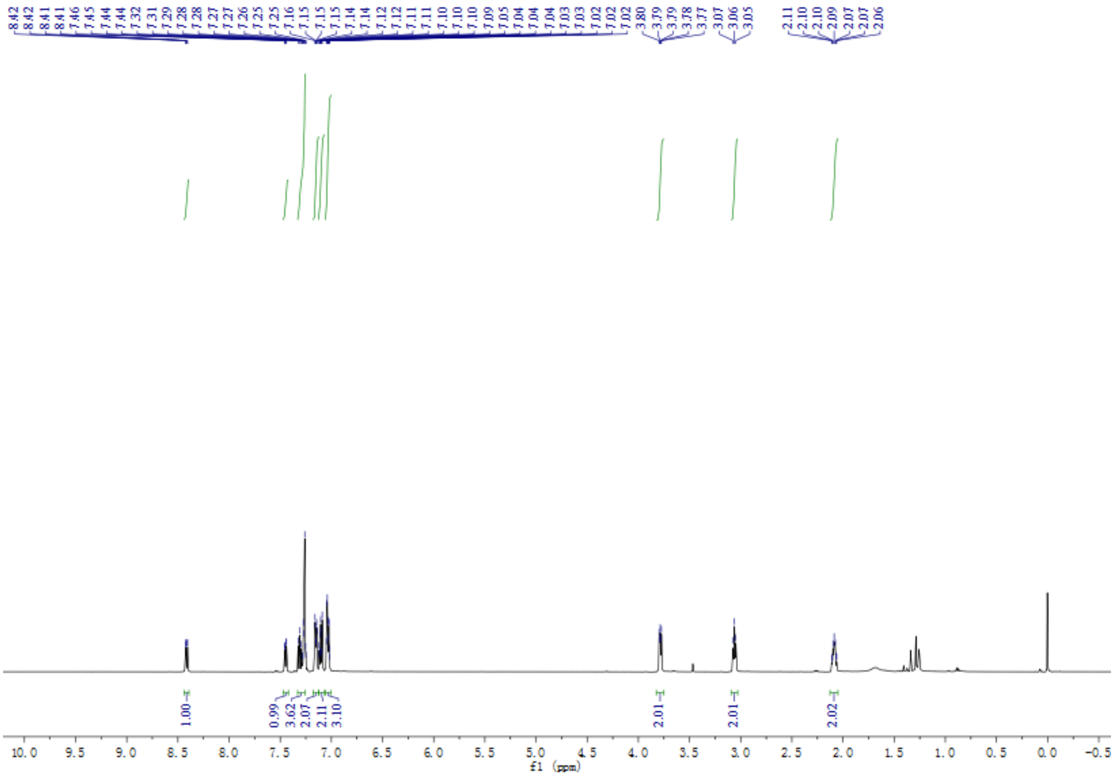

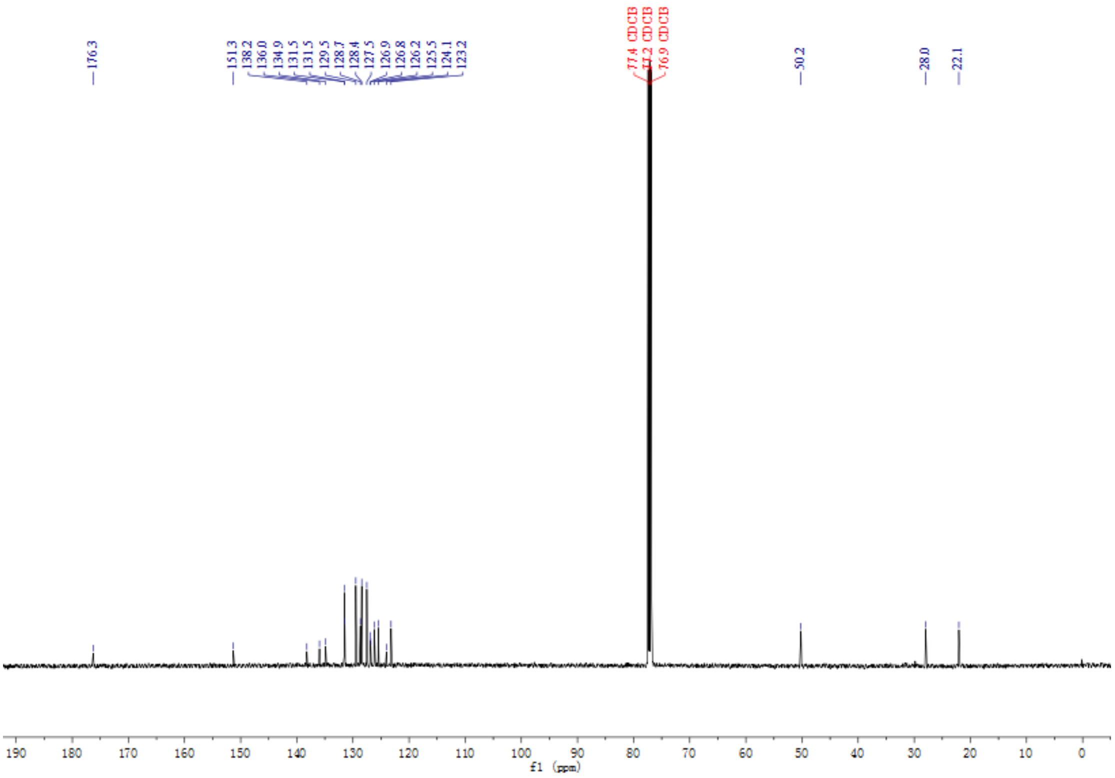

(15) The ^{1}H NMR and ^{13}C NMR spectrum for **3o**

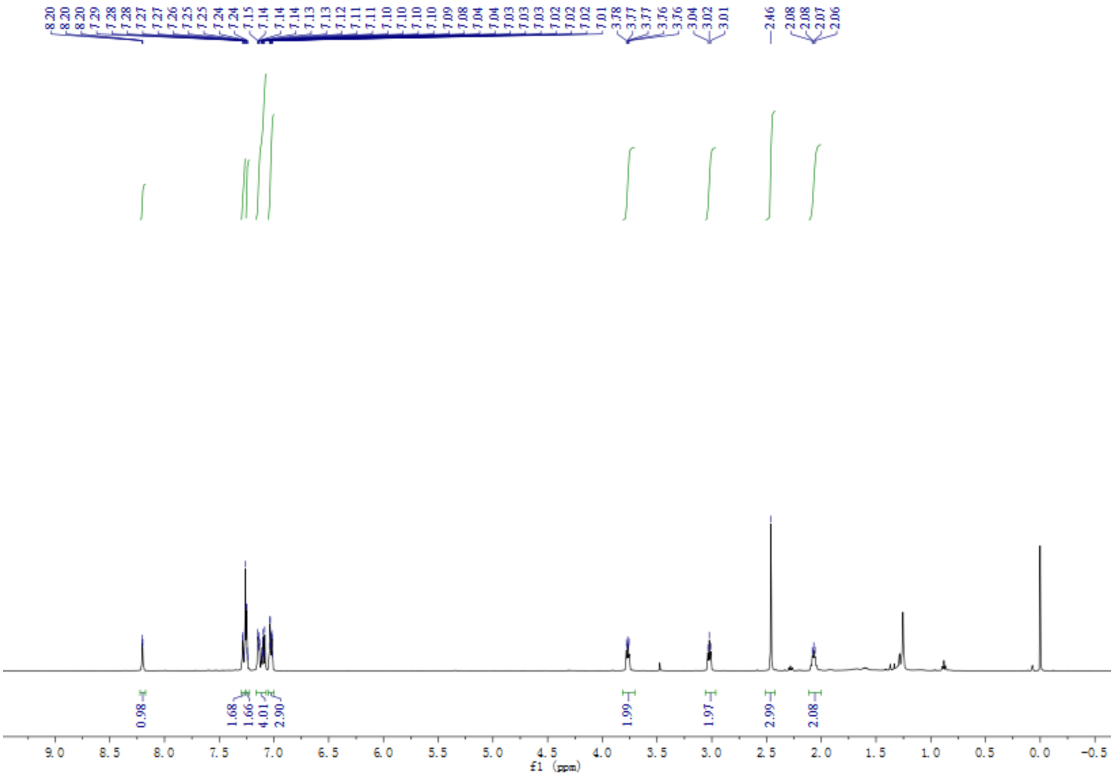

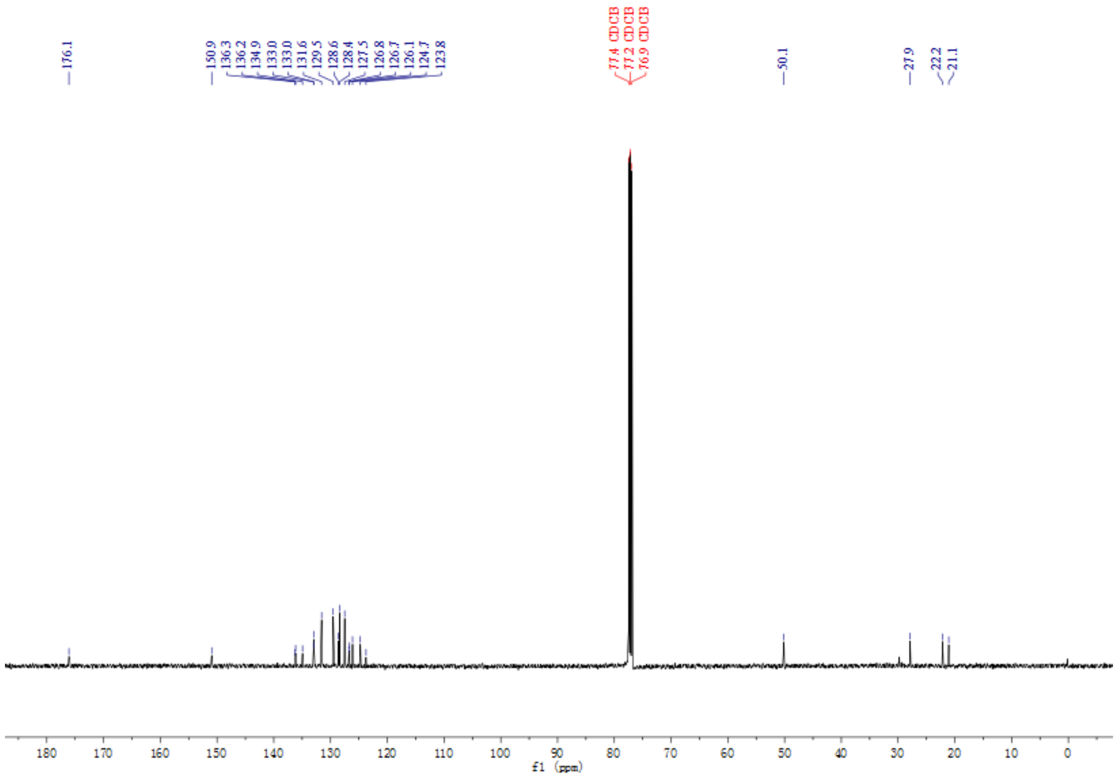

(16) The ^{1}H NMR and ^{13}C NMR spectrum for **3p**

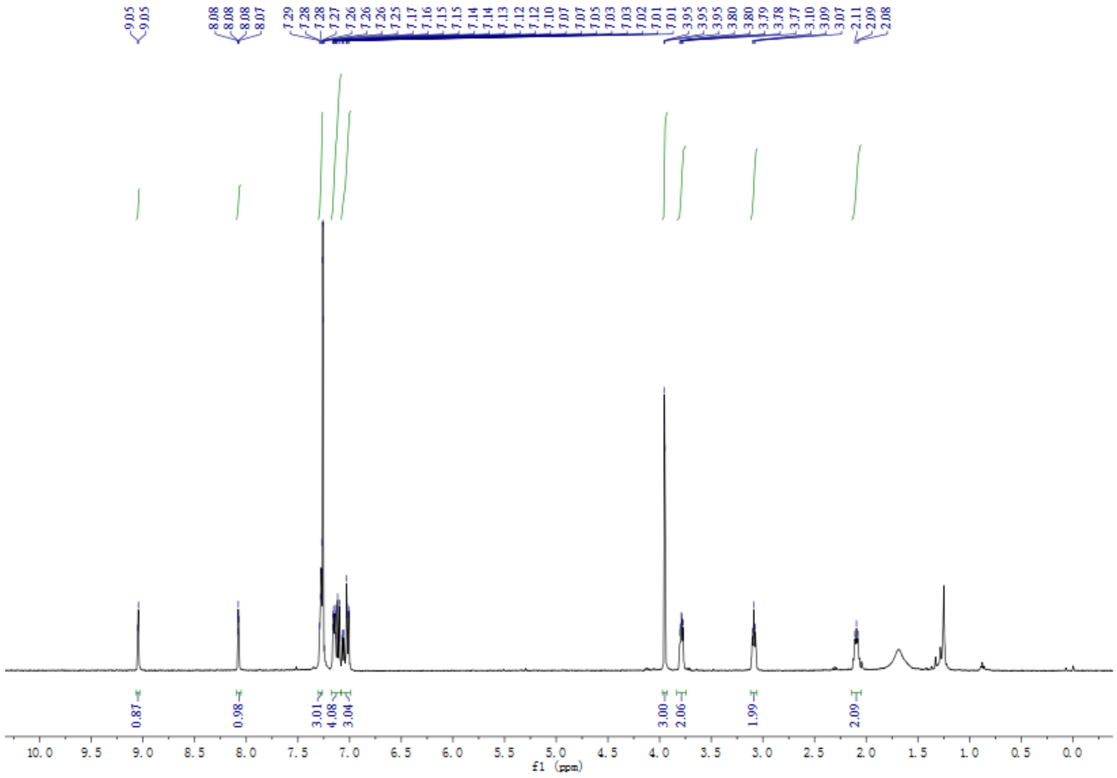

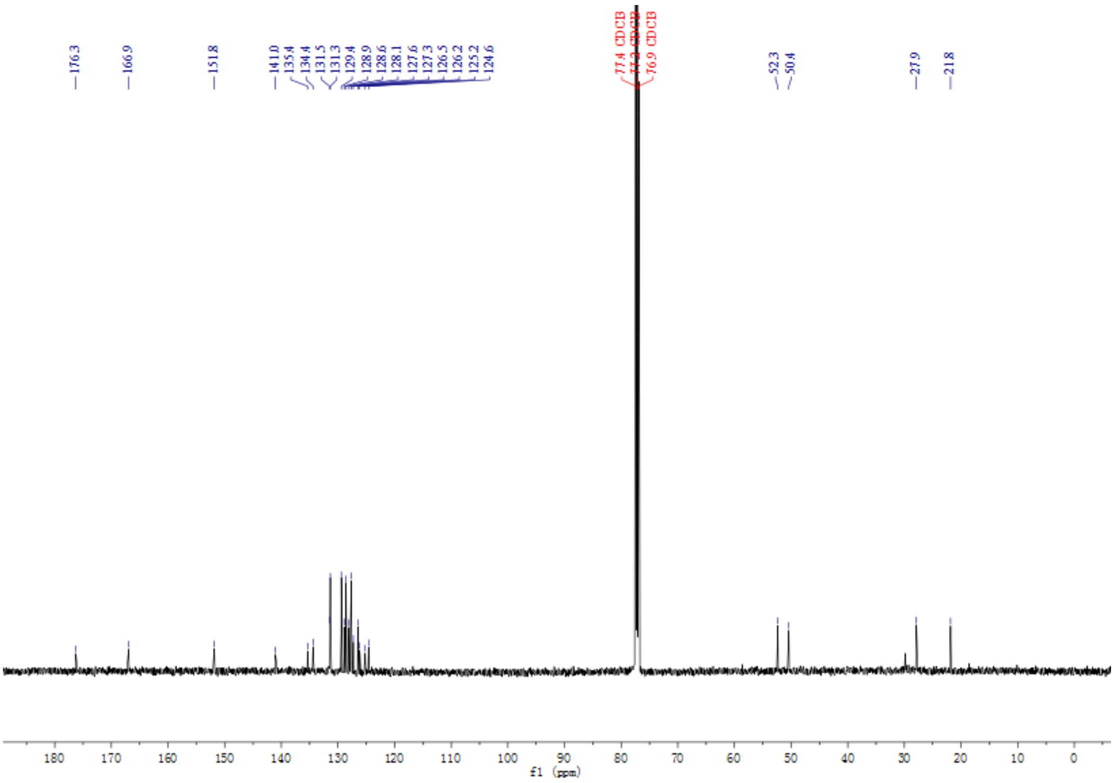

(17) The ^{1}H NMR and ^{13}C NMR spectrum for **4a**

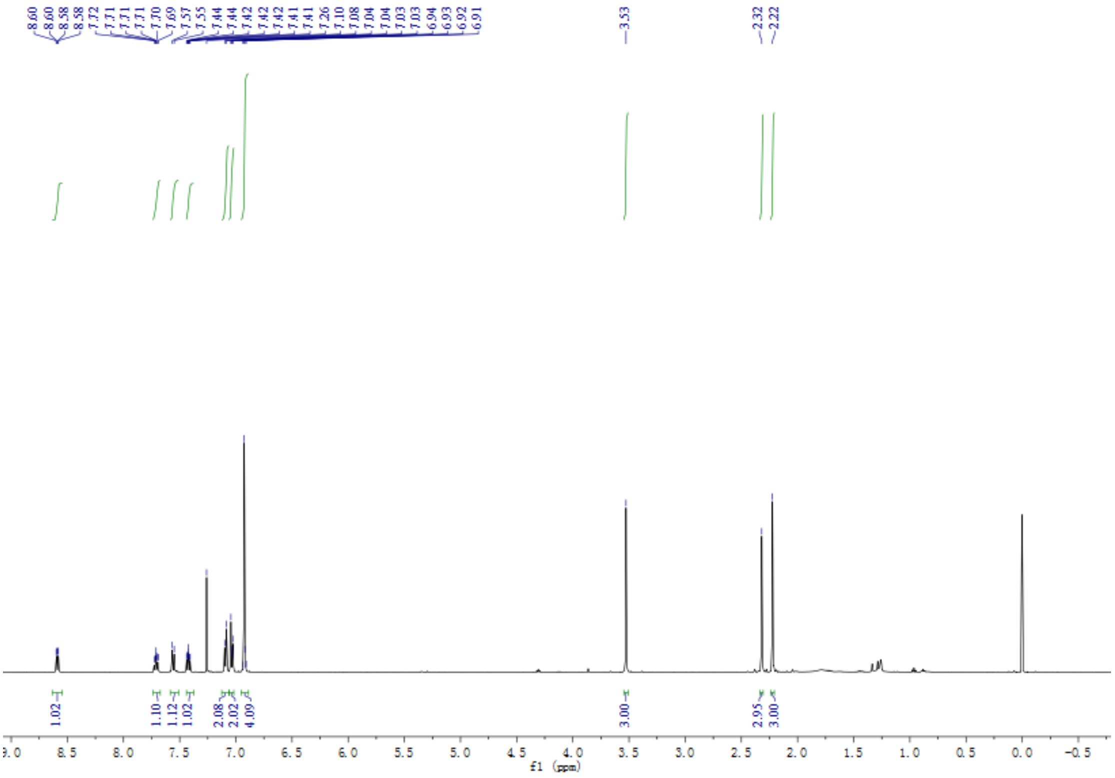

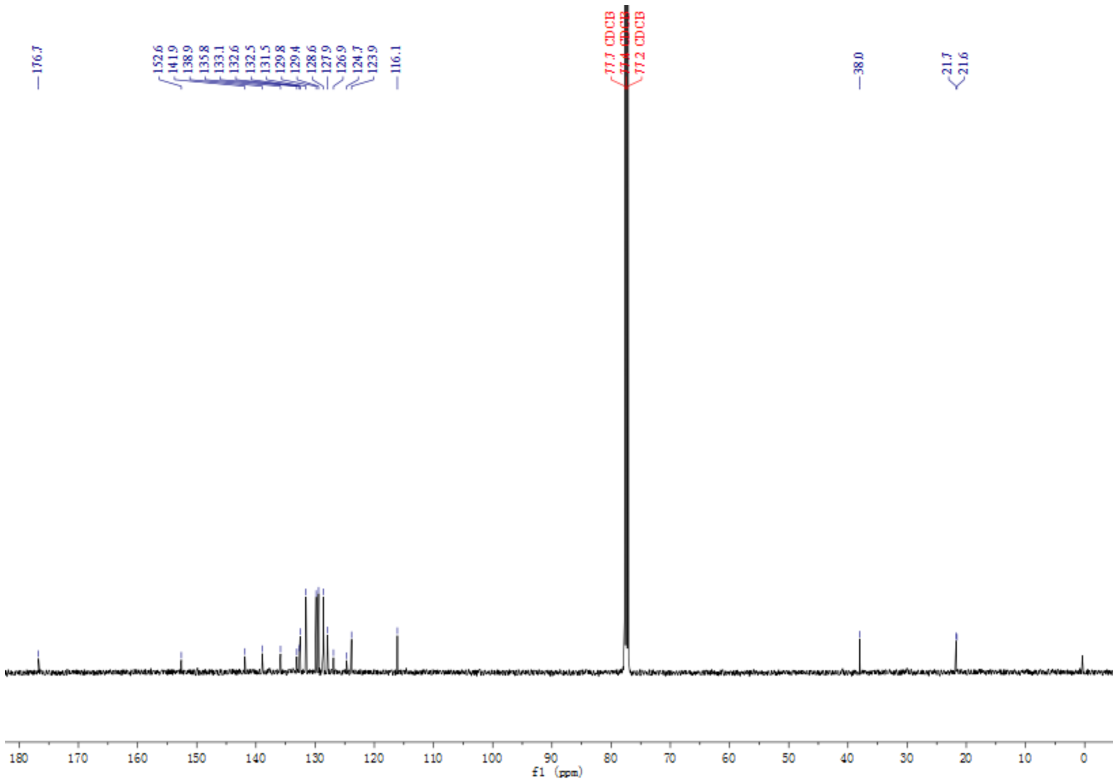

(18) The ^{1}H NMR and ^{13}C NMR spectrum for **4b**

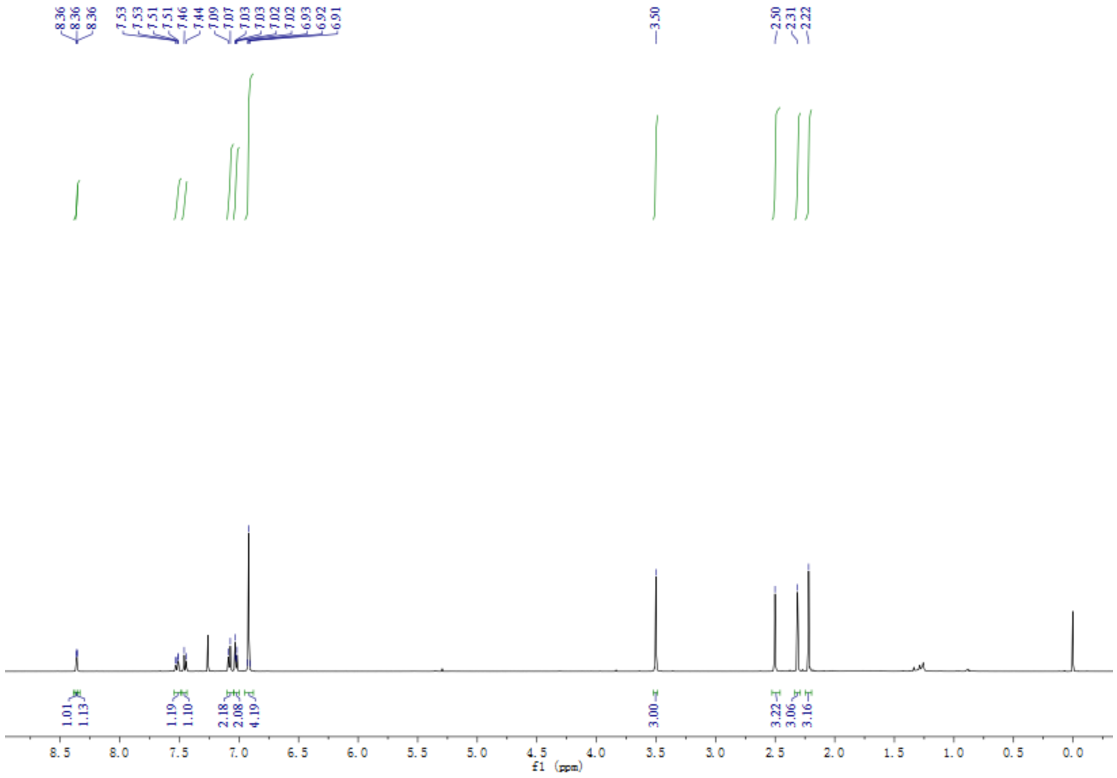

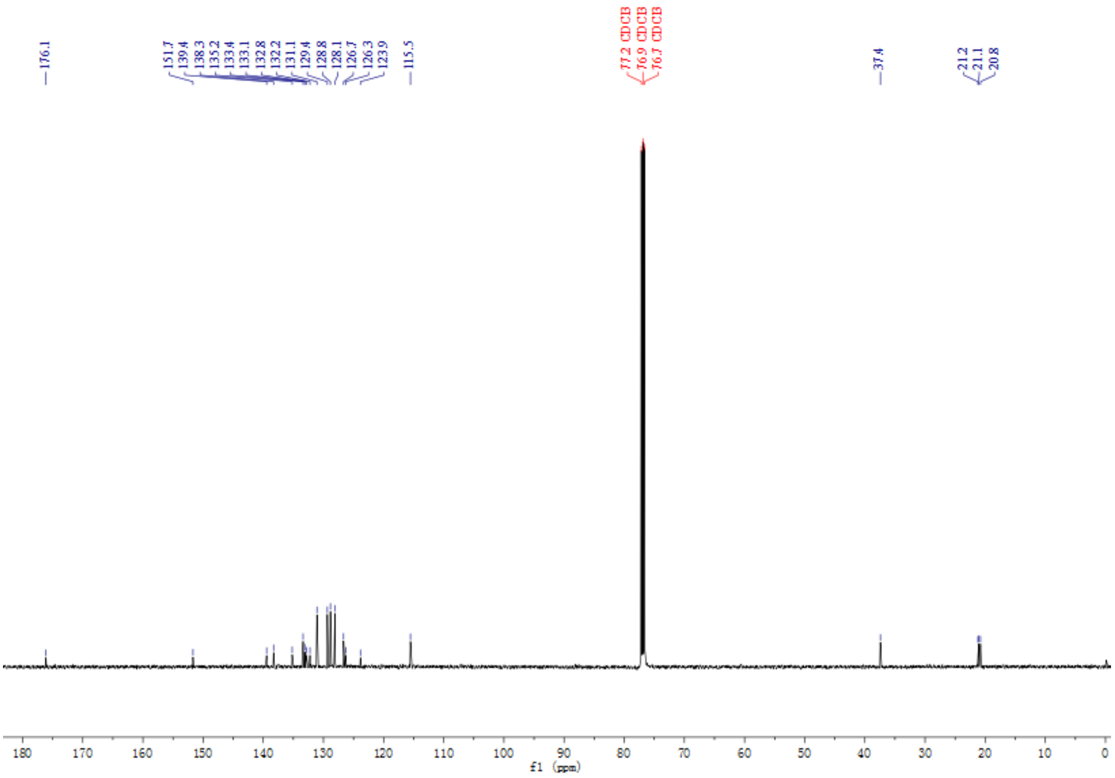

(19) The ^{1}H NMR and ^{13}C NMR spectrum for **4c**

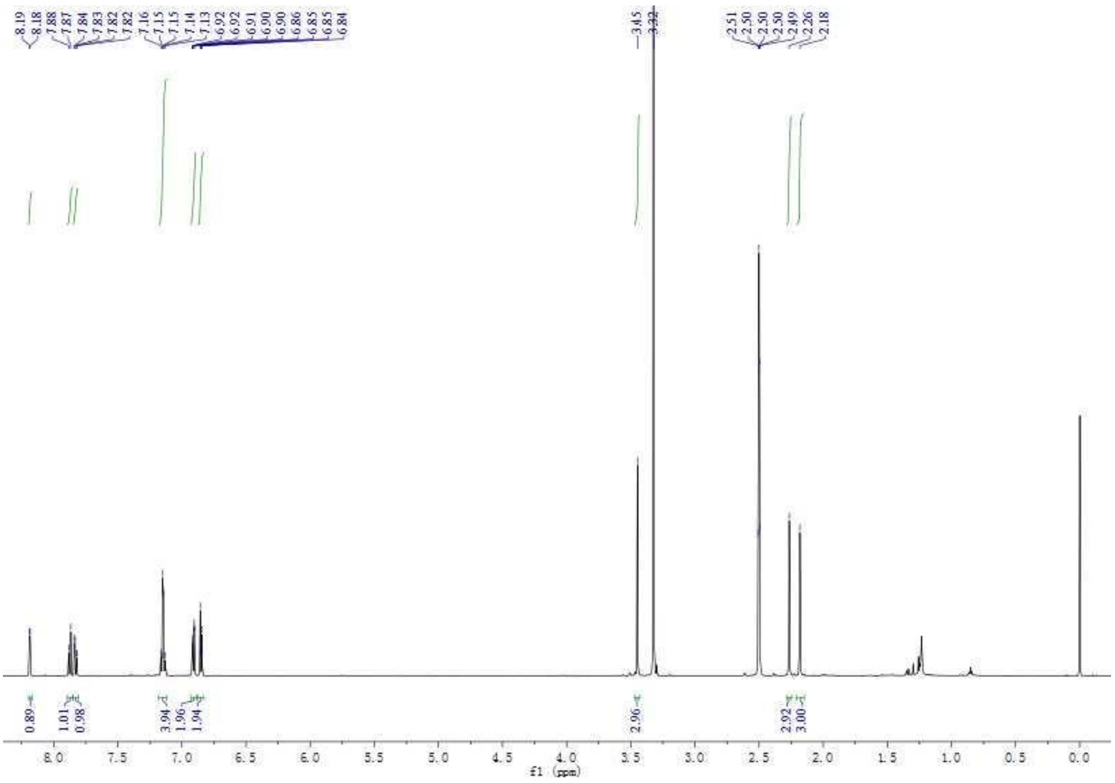

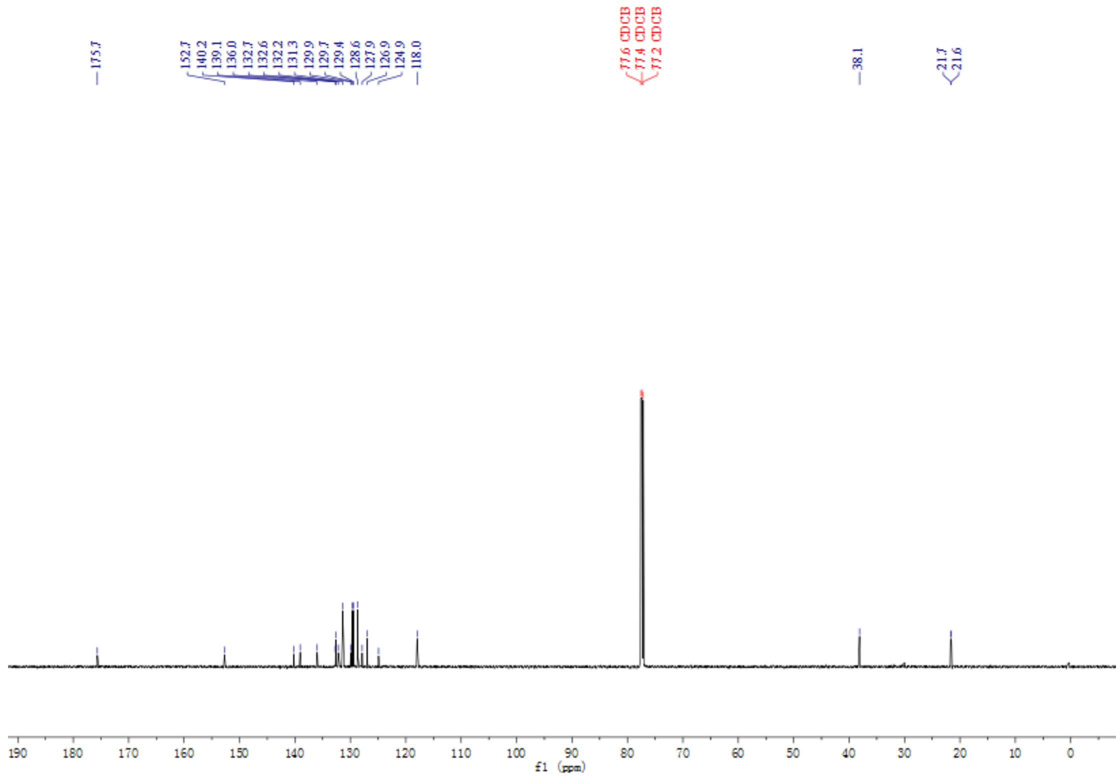

(20) The ^{1}H NMR and ^{13}C NMR spectrum for **4d**

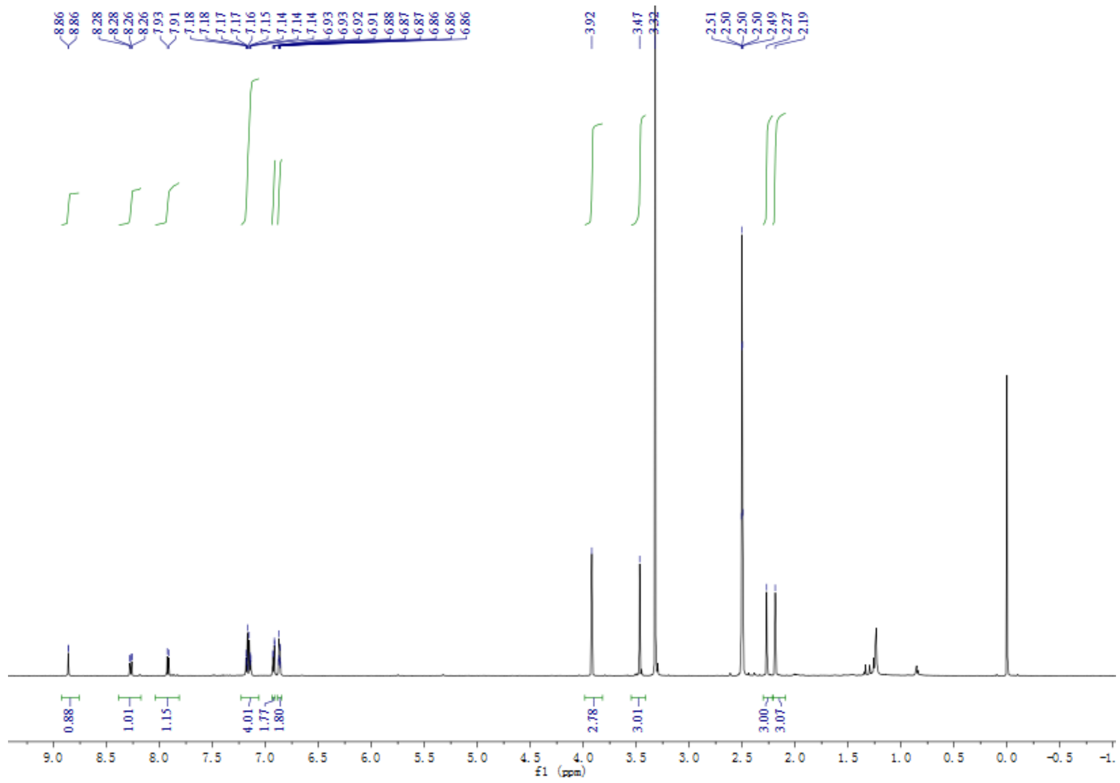

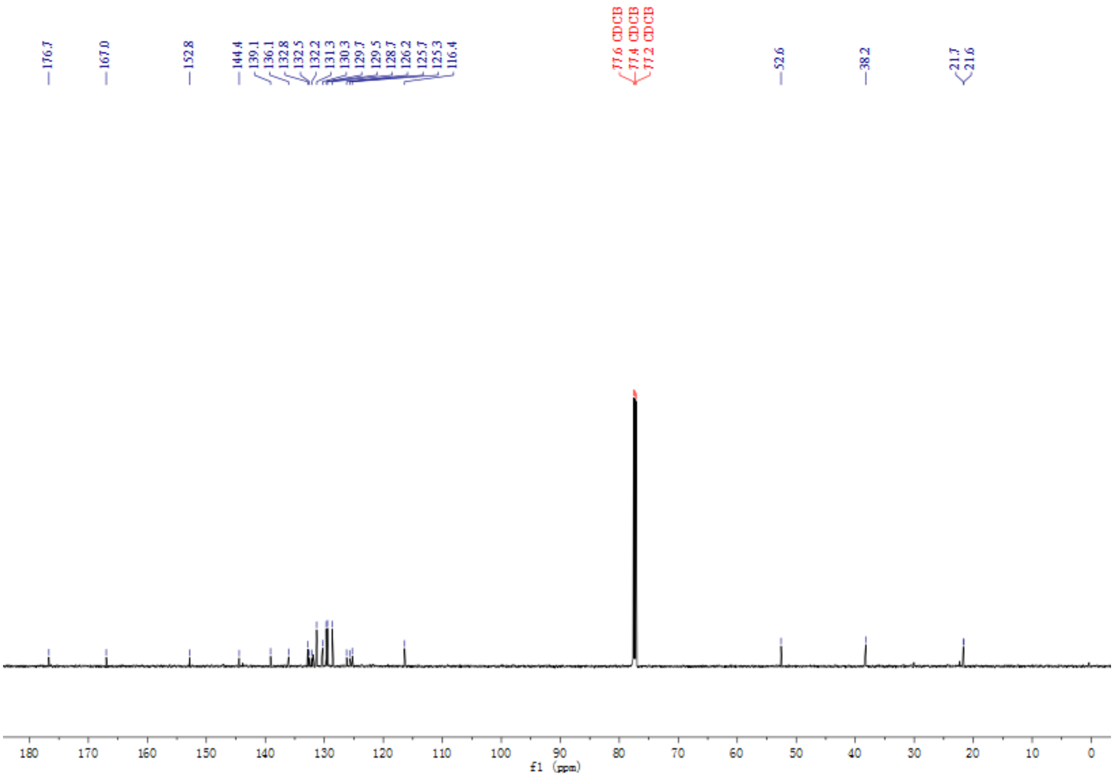

(21) The ^{1}H NMR and ^{13}C NMR spectrum for **4e**

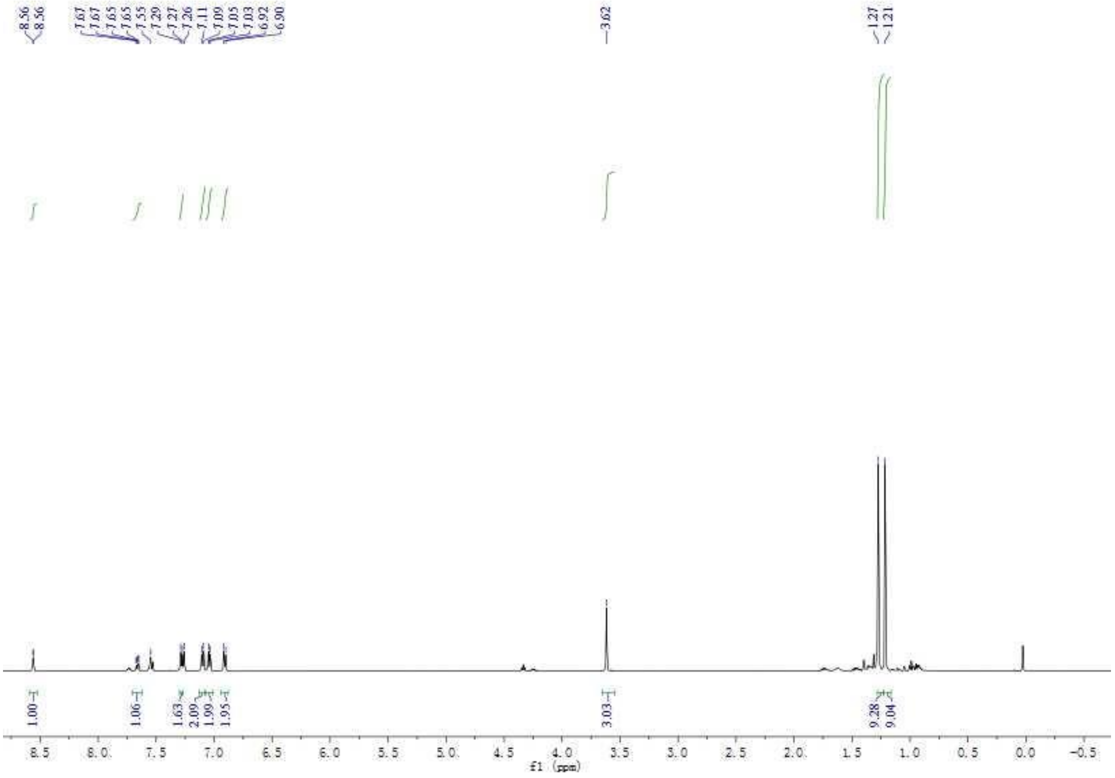

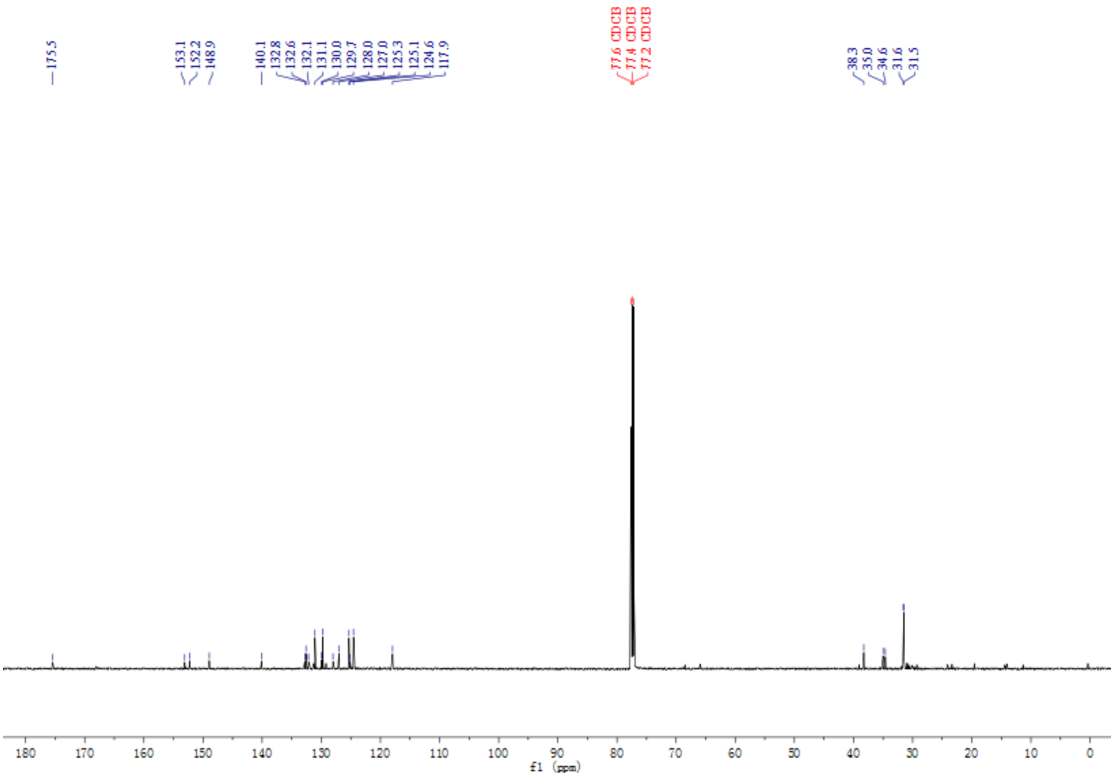

(22) The ^{1}H NMR and ^{13}C NMR spectrum for **4f**

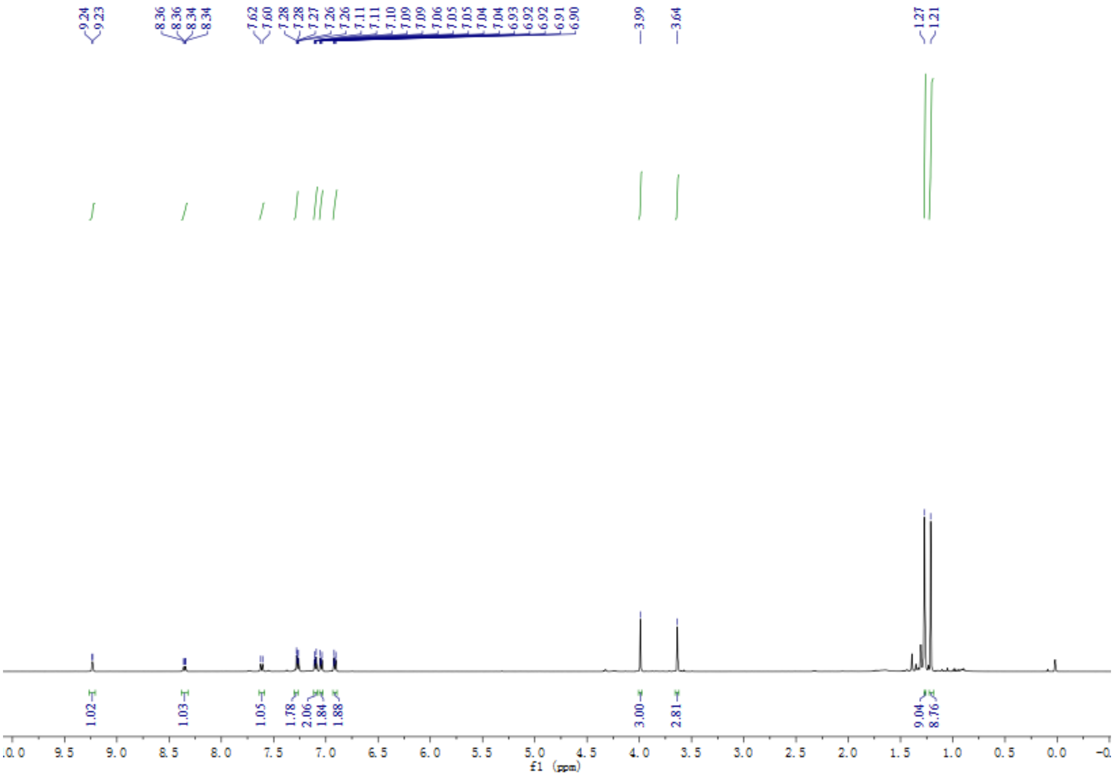

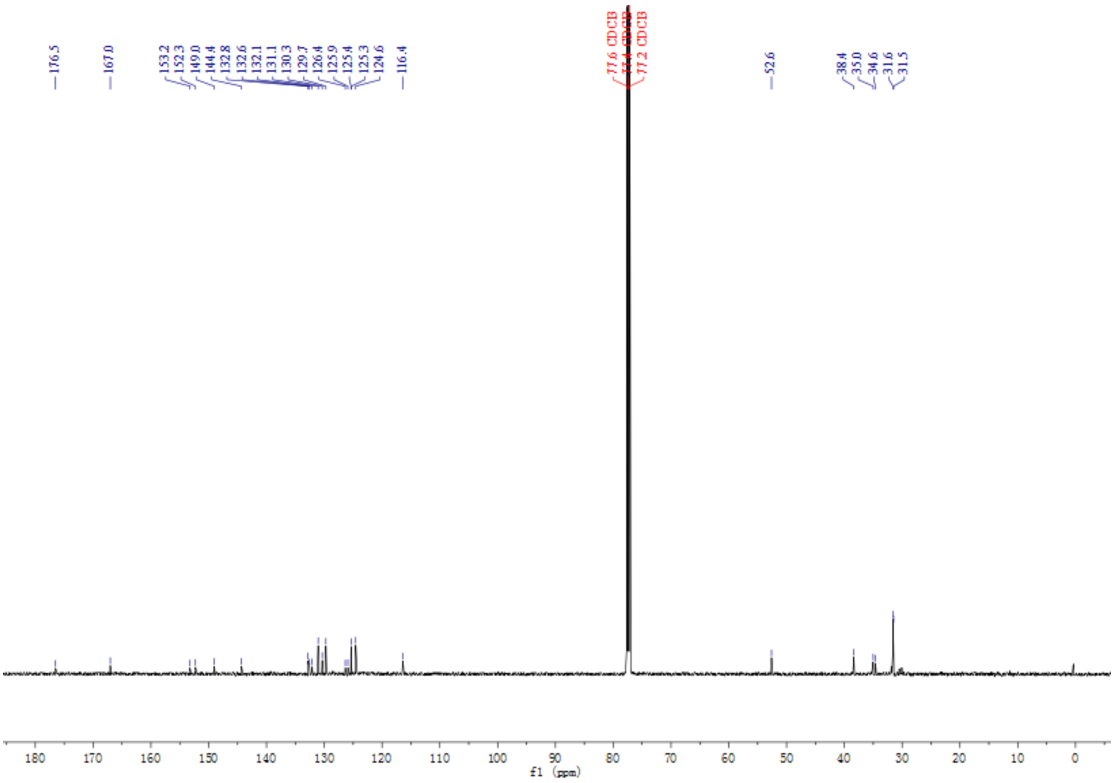

(23) The ^{1}H NMR and ^{13}C NMR spectrum for **4g**

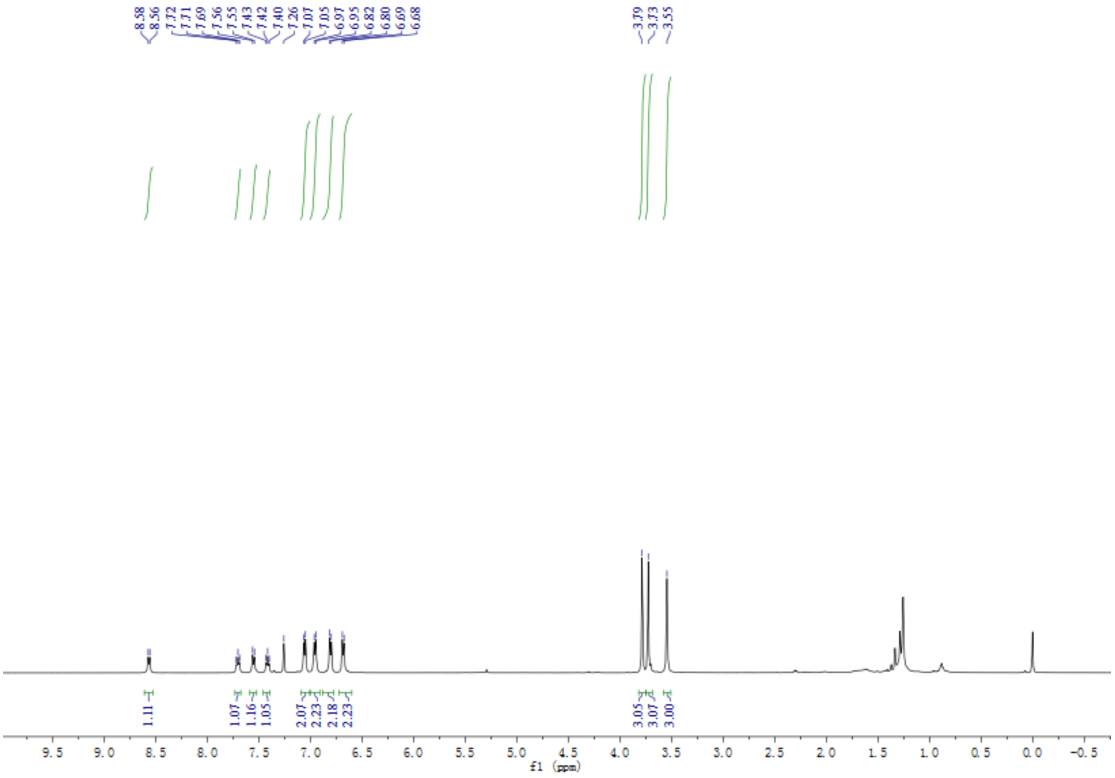

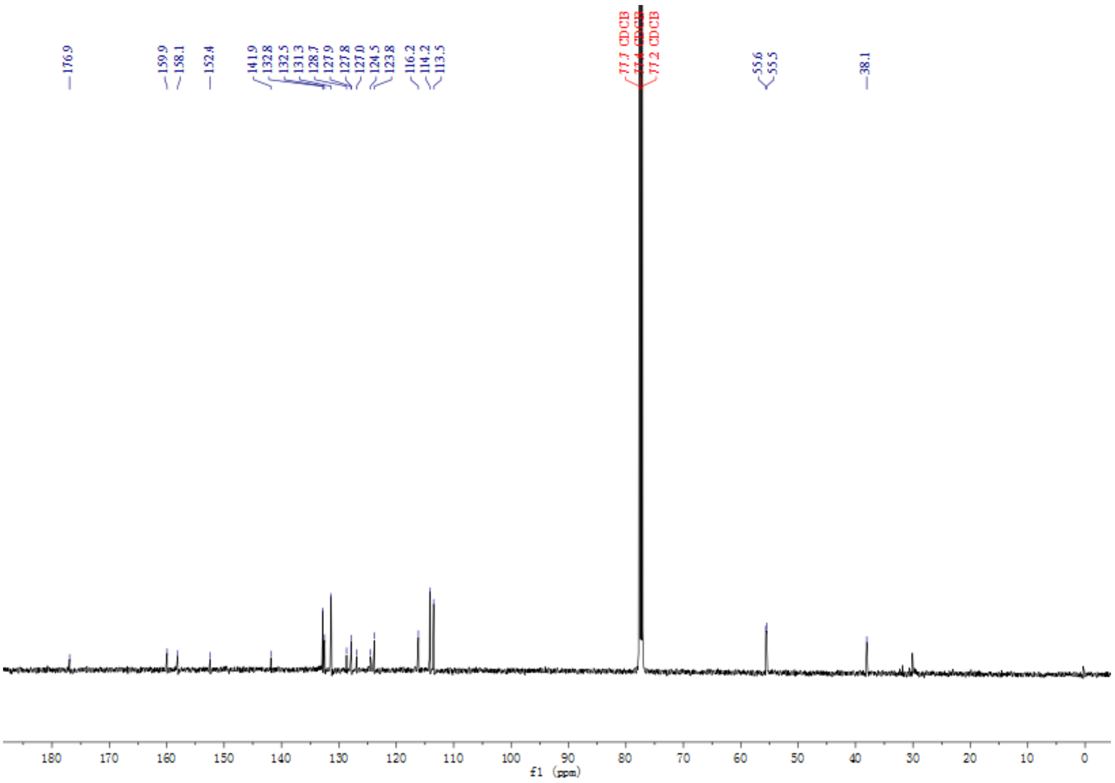

(24) The ^{1}H NMR and ^{13}C NMR spectrum for **4h**

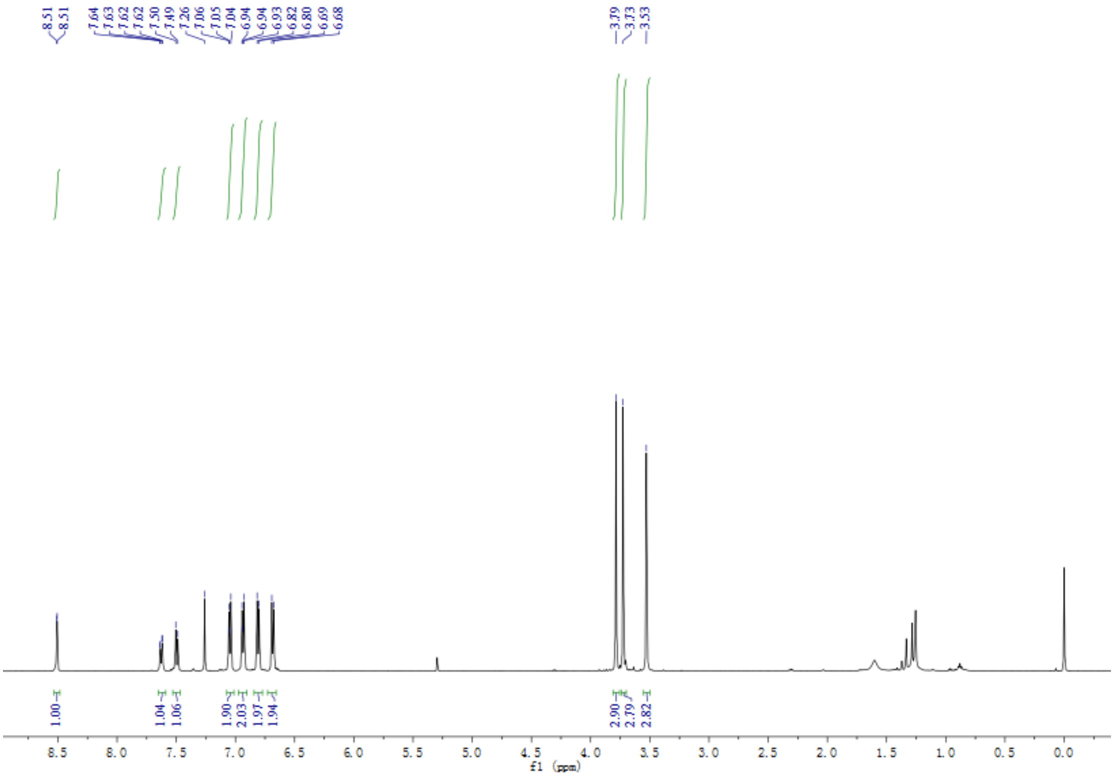

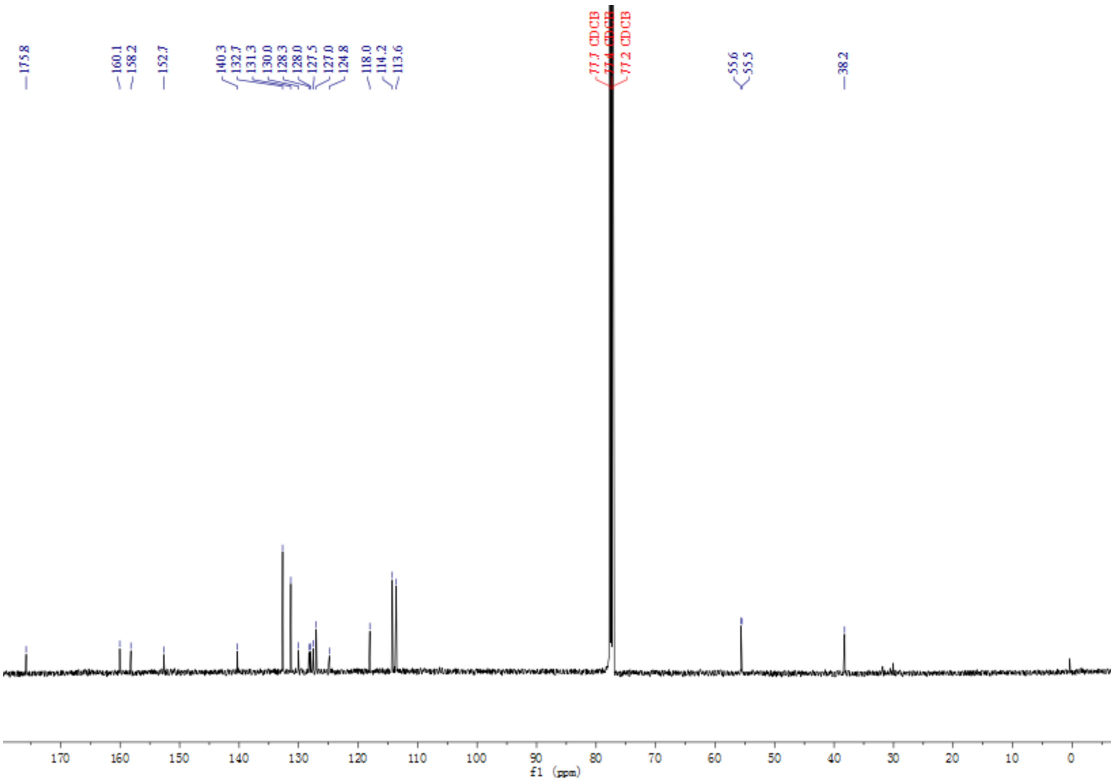

(25) The ^{1}H NMR and ^{13}C NMR spectrum for **4i**

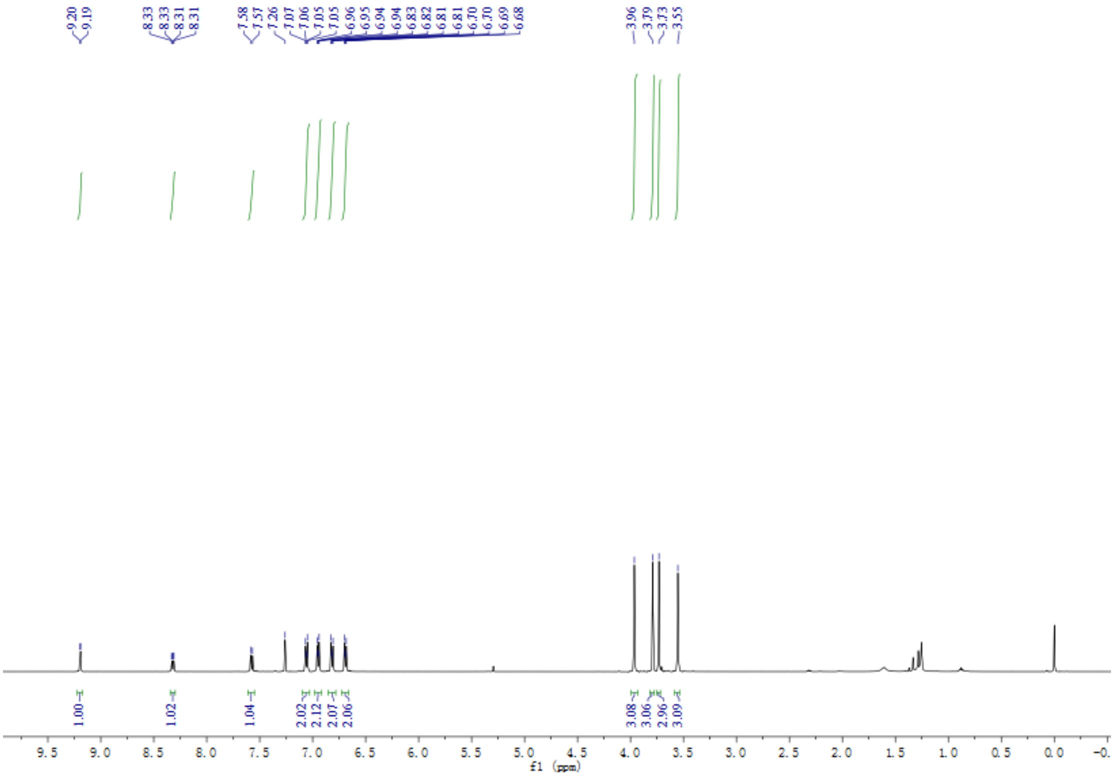

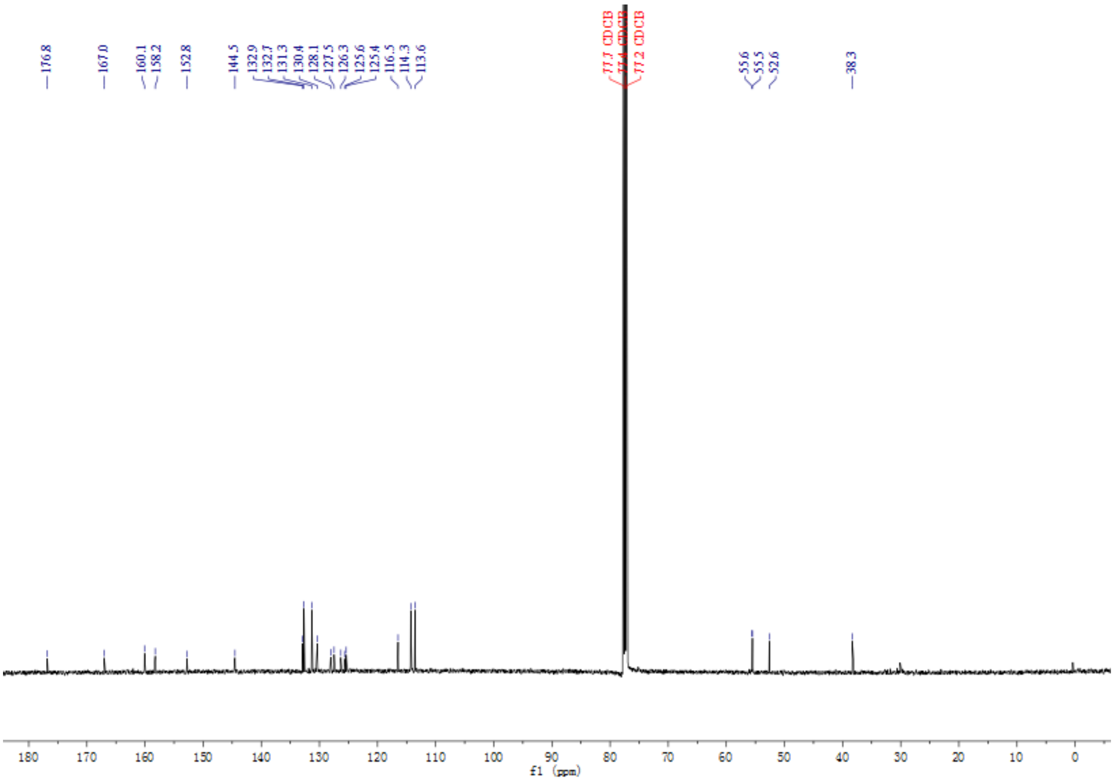

(26) The ^{1}H-NMR, ^{13}C-NMR and ^{19}F-NMRspectra for **4j**

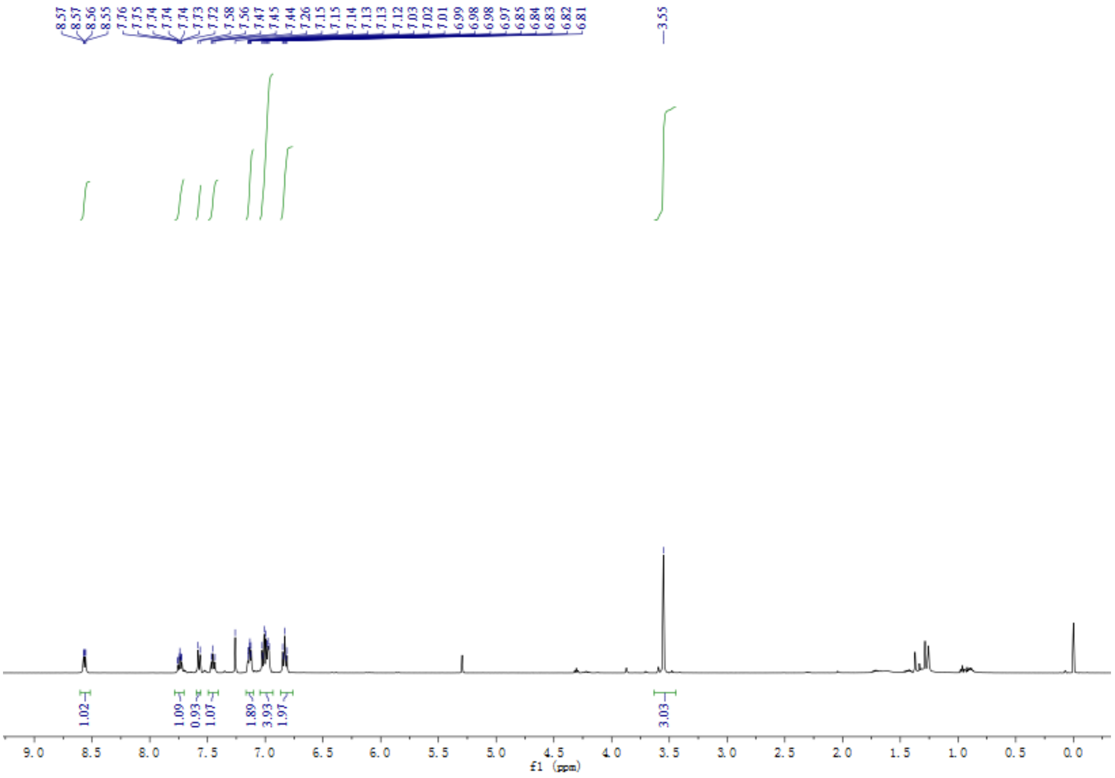

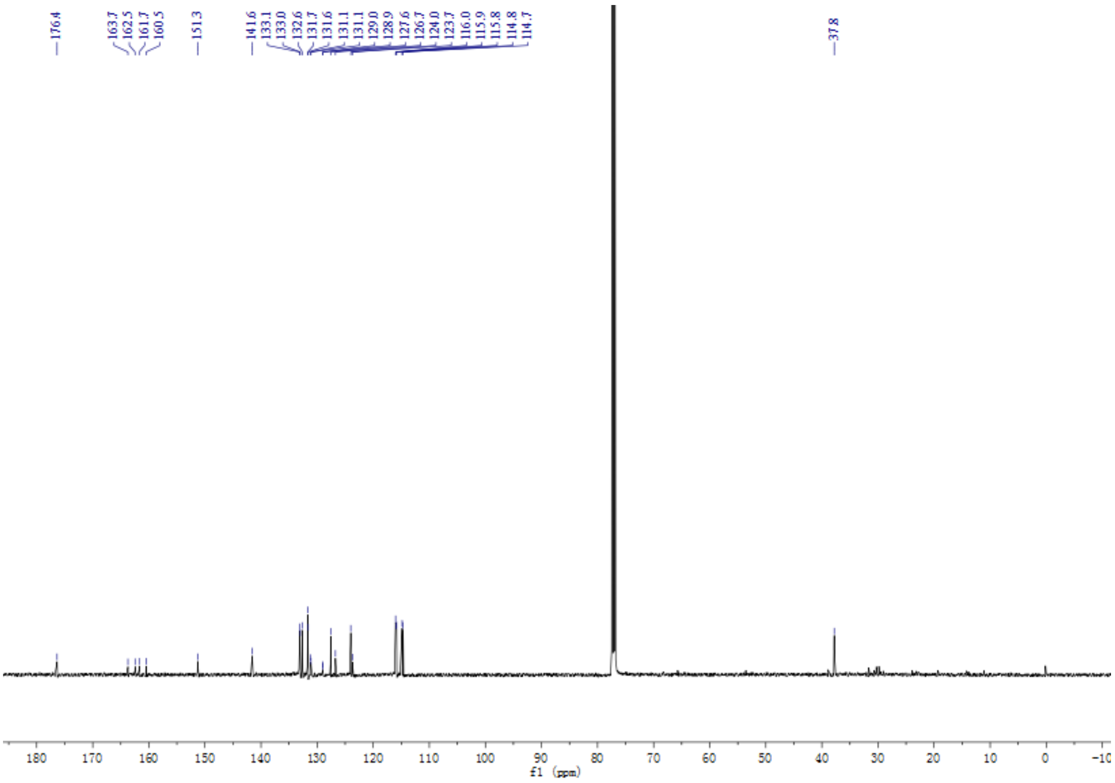
176.4
163.7
162.5
161.7
160.5
151.3
141.6
133.1
133.0
132.6
131.7
131.6
131.1
131.1
129.0
128.9
127.6
126.7
124.0
123.7
116.0
115.9
115.8
114.8
114.7
37.8
f1 (ppm)

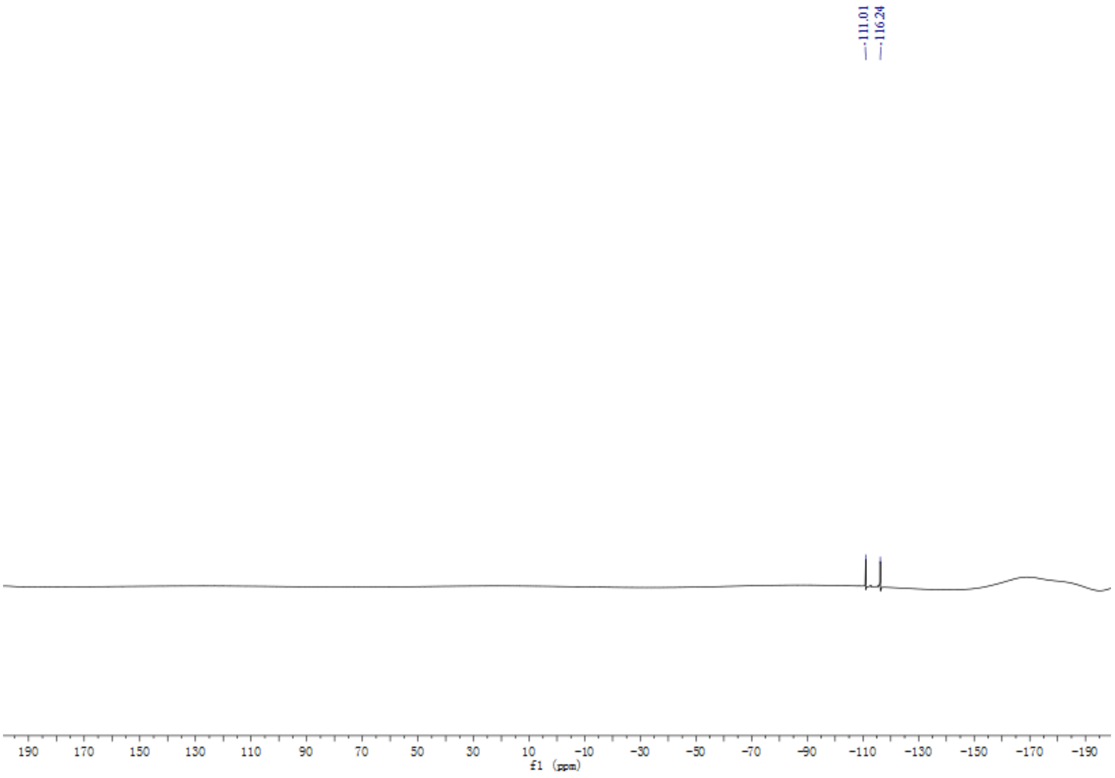
-111.01
-116.24
f1 (ppm)

(27) The ^{1}H-NMR, ^{13}C-NMR and ^{19}F-NMRspectra for **4k**

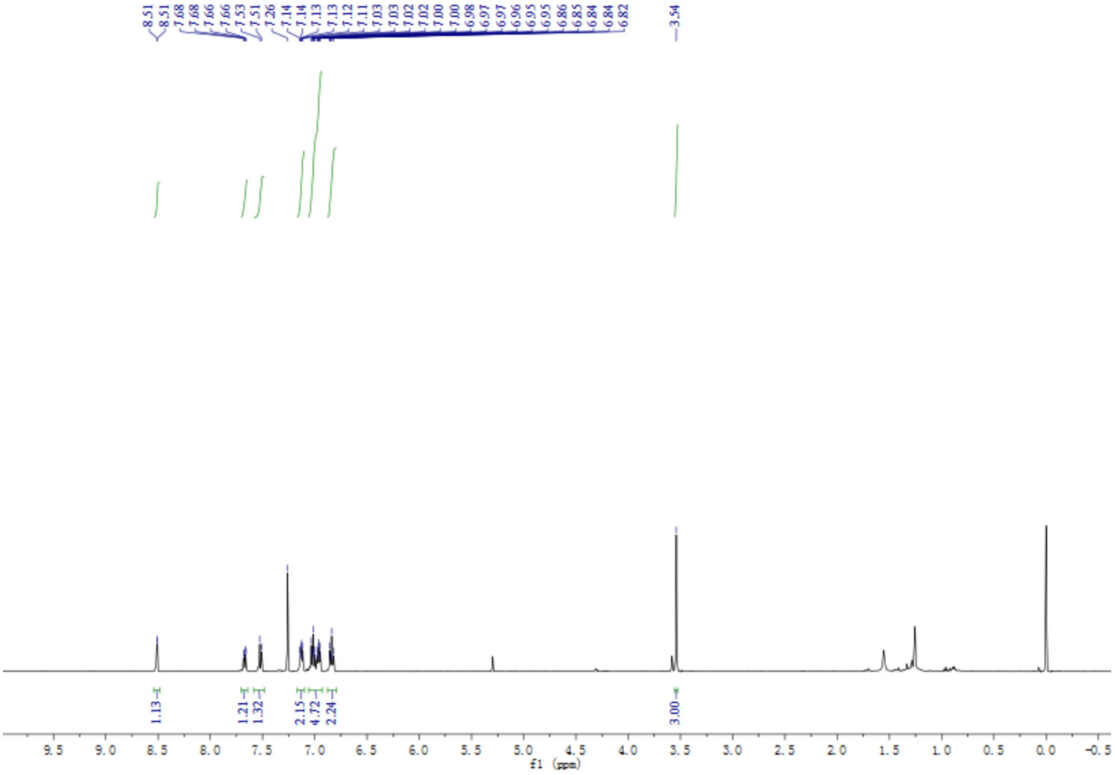

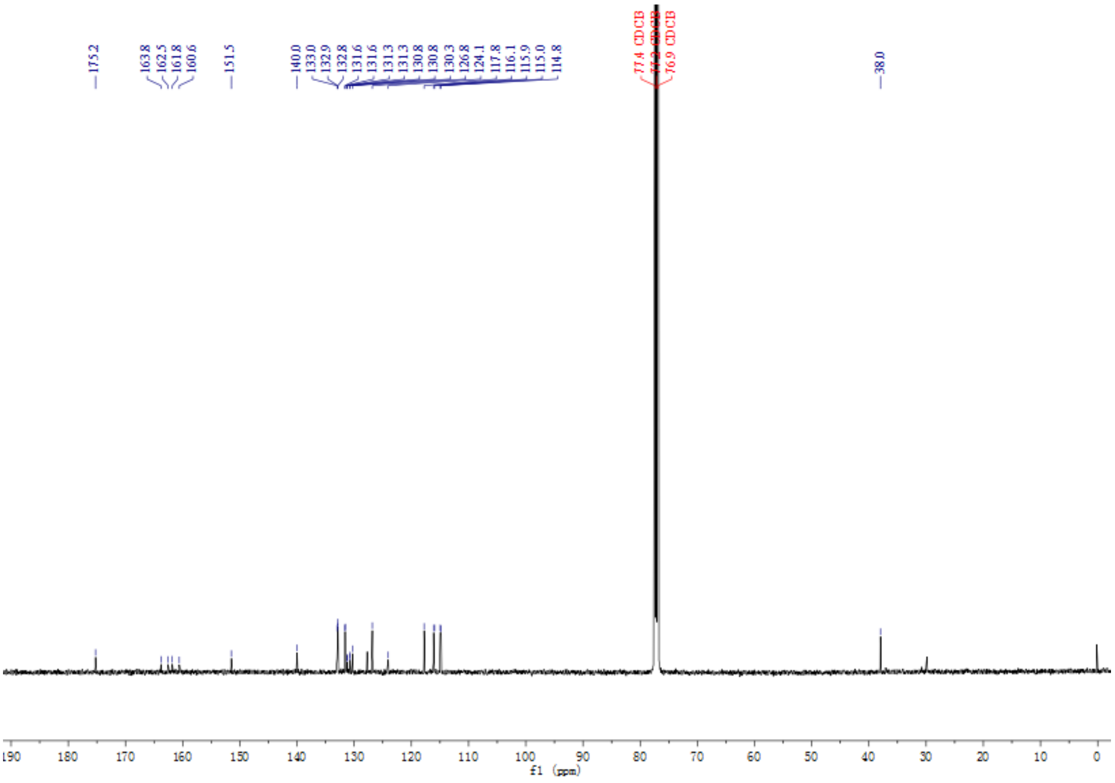

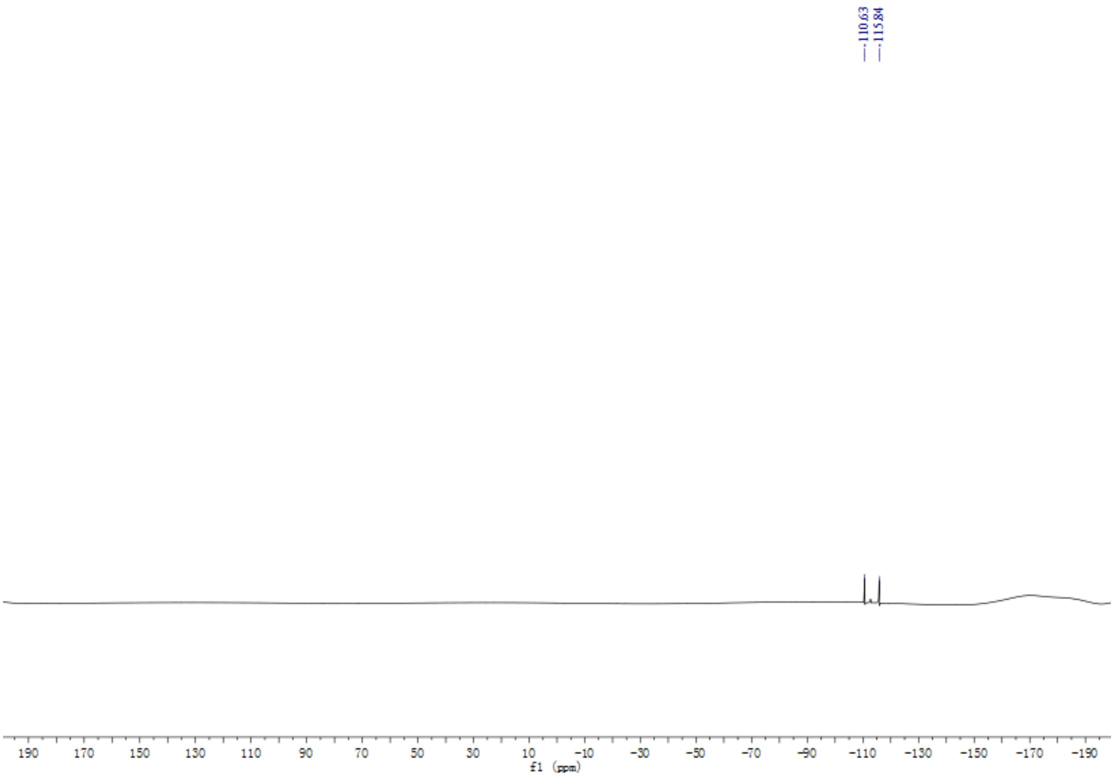

(28) The ¹H-NMR, ¹³C-NMR and ¹⁹F-NMRspectra for **4l**

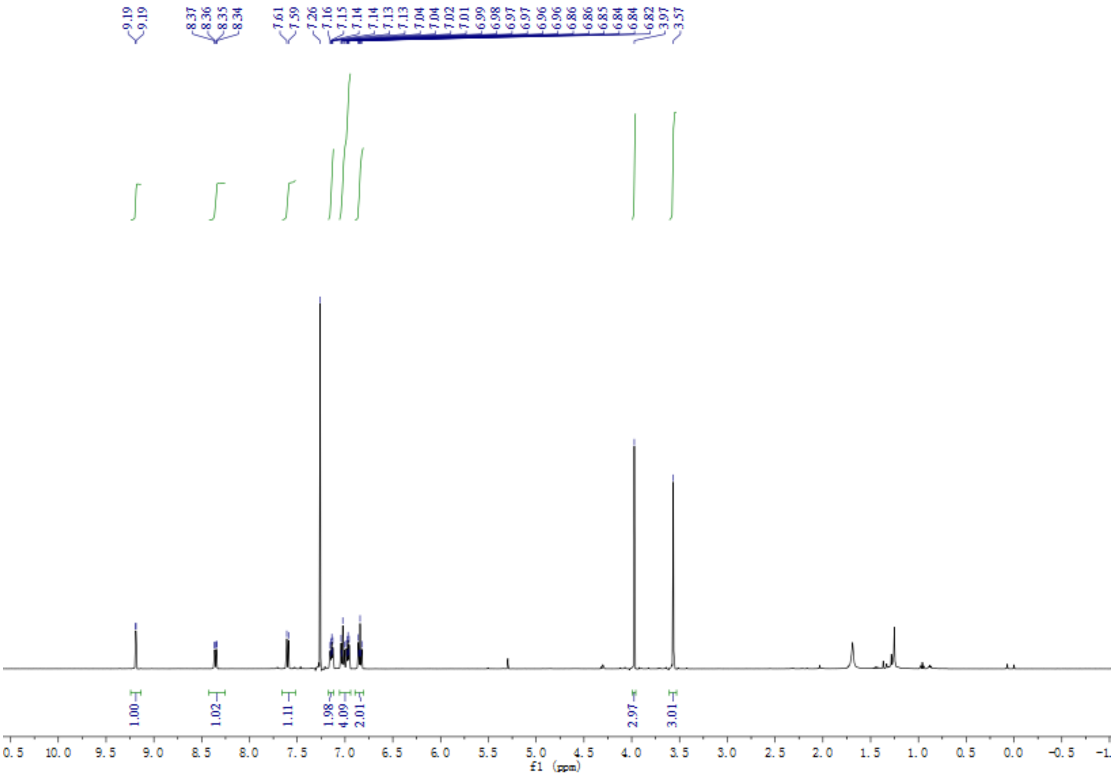

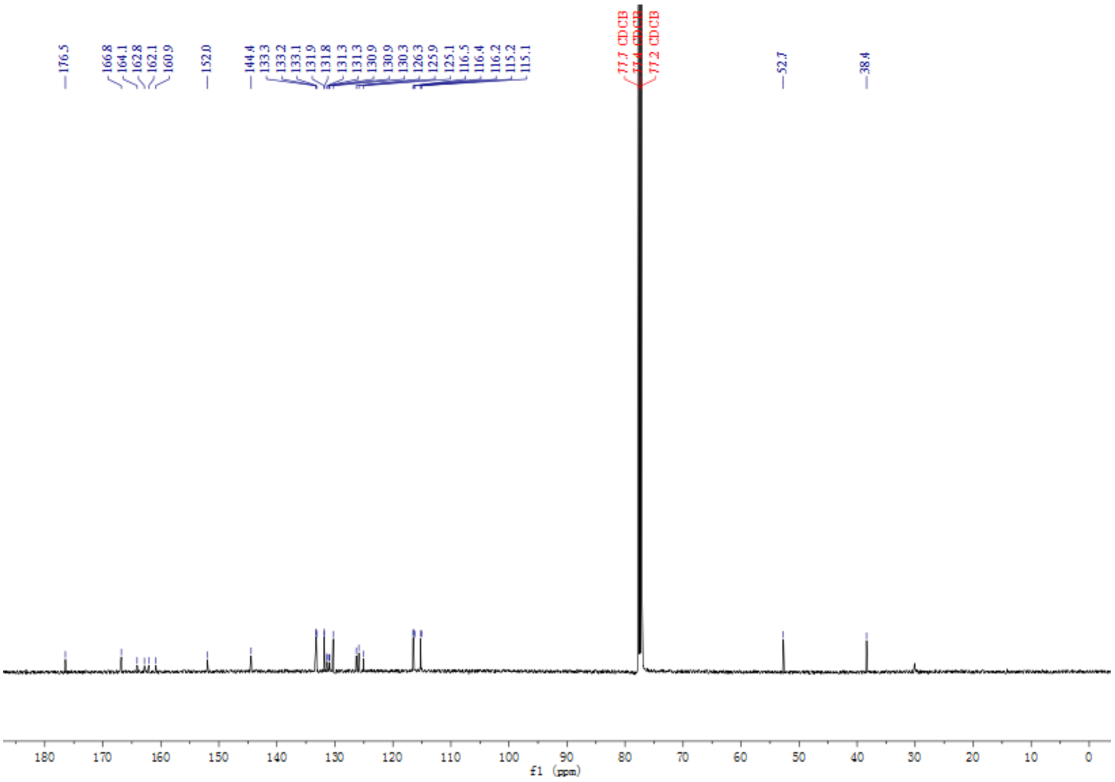
176.5
166.8
164.1
162.8
162.1
160.9
152.0
144.4
133.3
133.2
133.1
131.9
131.8
131.3
131.3
130.9
130.9
130.3
126.3
125.9
125.1
116.5
116.4
116.2
115.2
115.1
77.7 CDCl3
77.2 CDCl3
52.7
38.4
f1 (ppm)

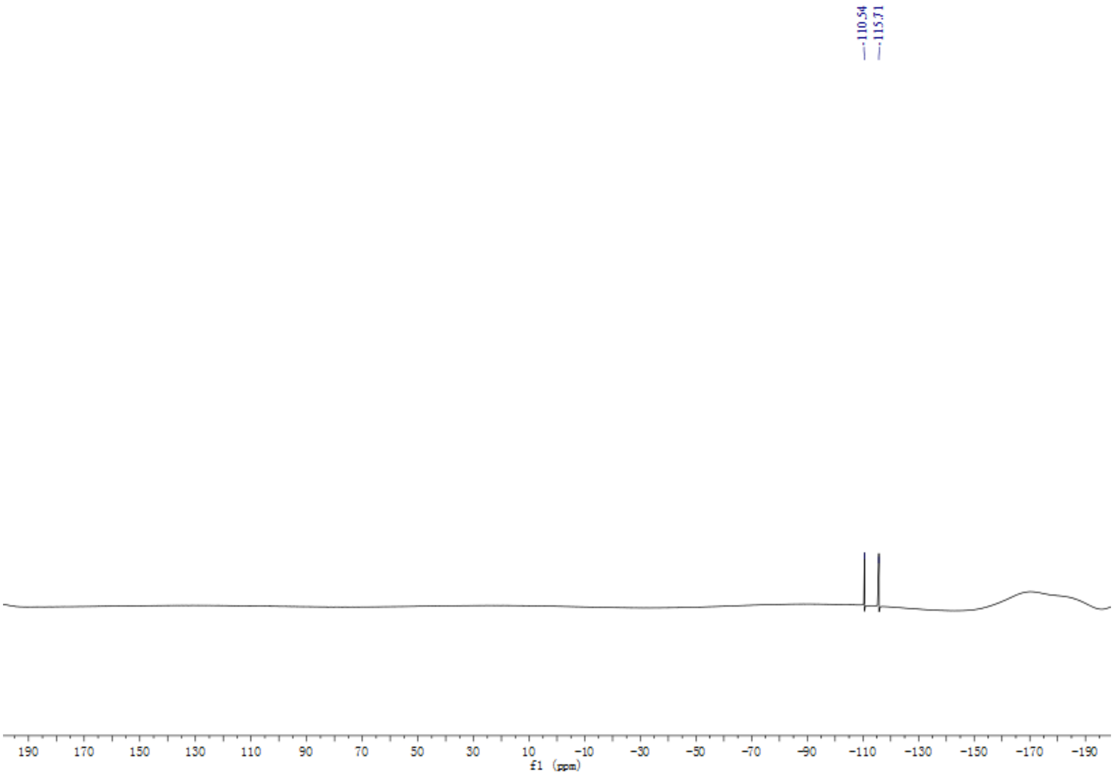
-110.54
-115.71
f1 (ppm)

(29) The ^{1}H NMR and ^{13}C NMR spectrum for **4m**

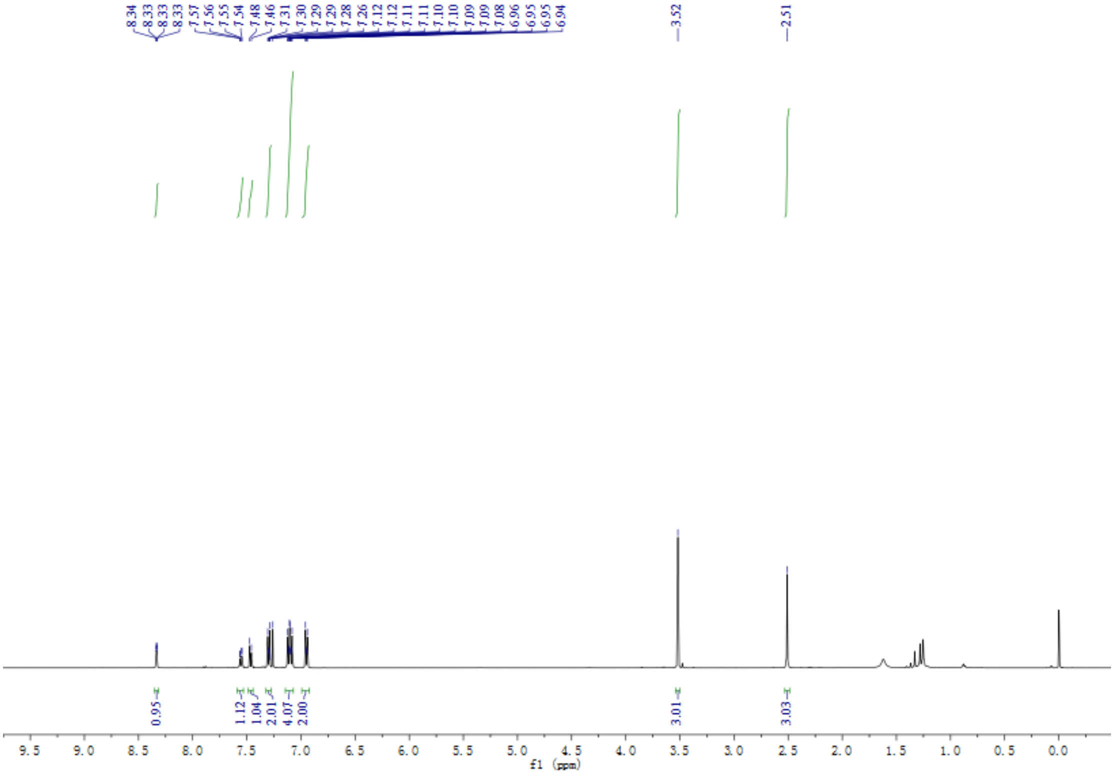

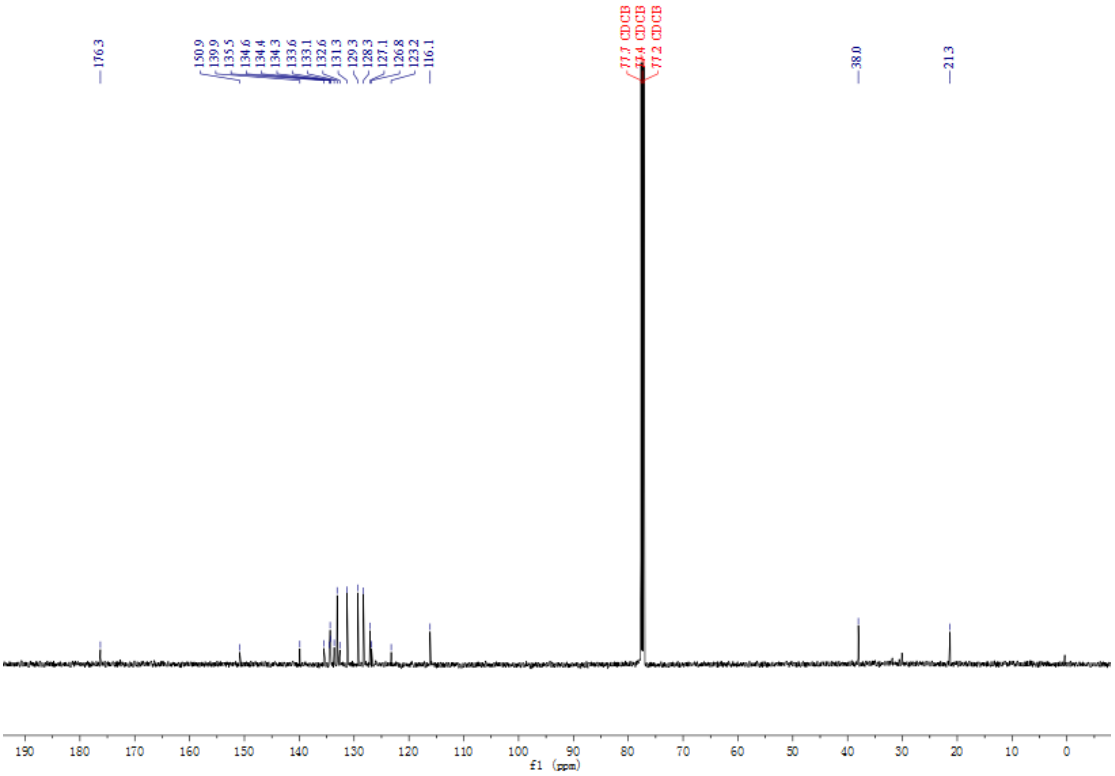

(30) The ^{1}H NMR and ^{13}C NMR spectrum for **4n**

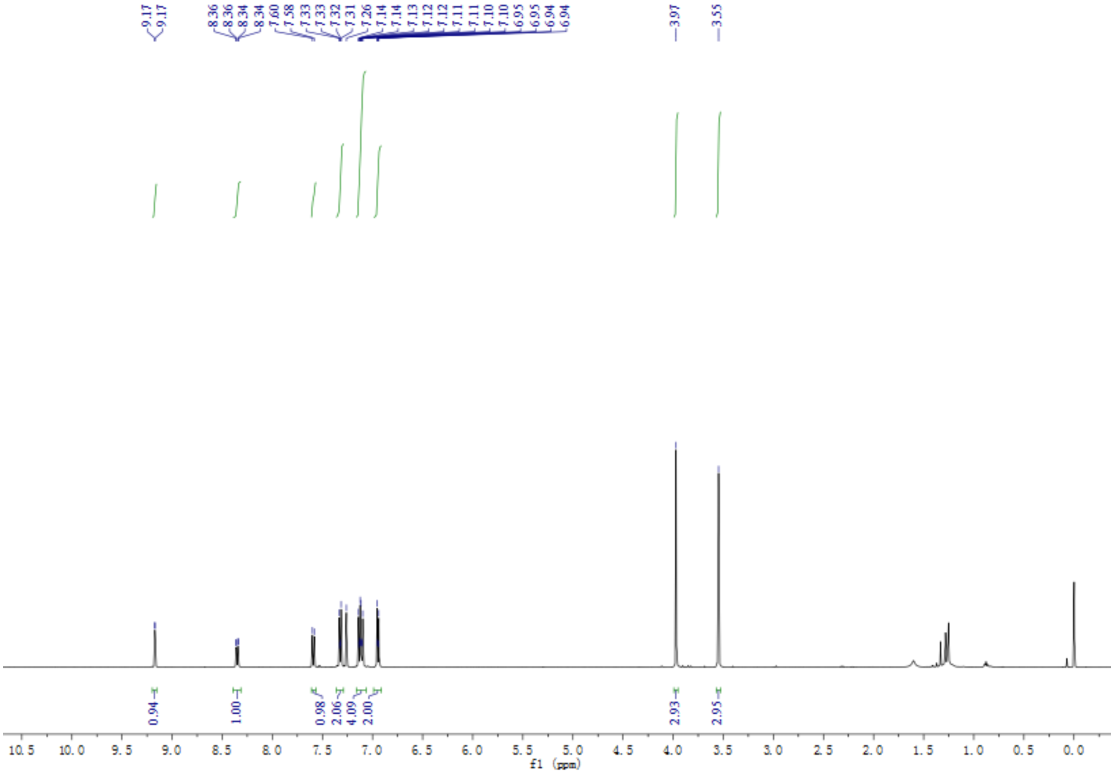

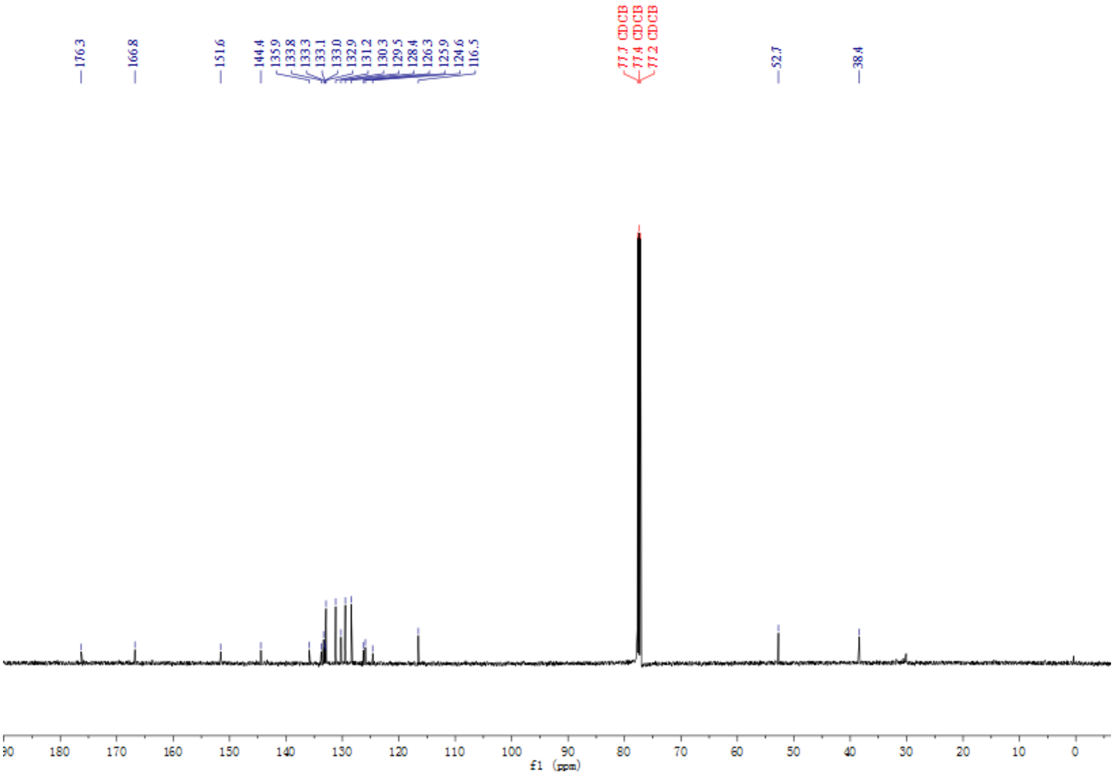

(31) The ^{1}H NMR and ^{13}C NMR spectrum for **5**

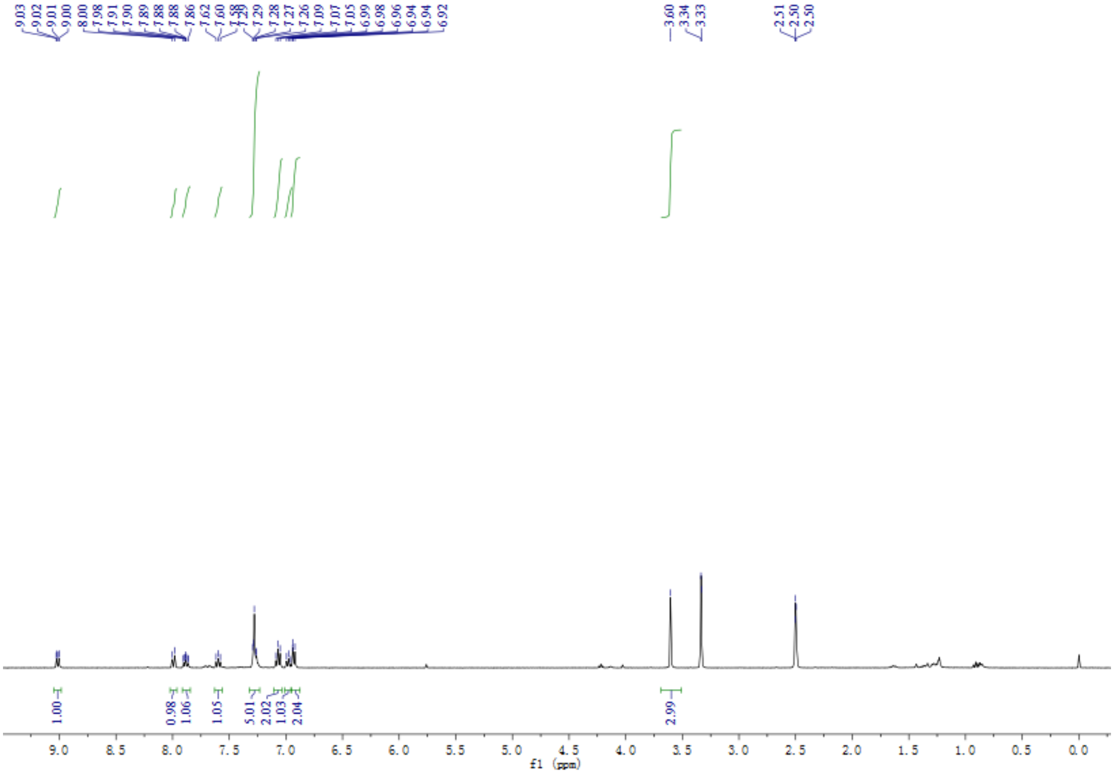

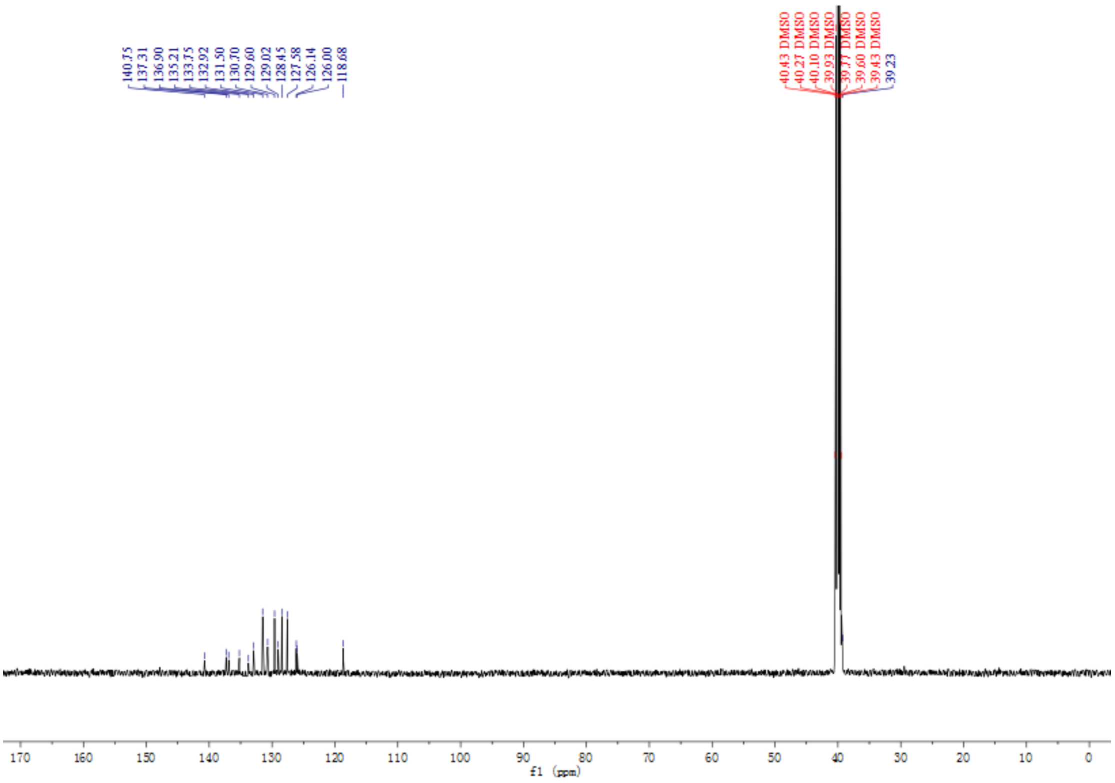

(32) The ^{1}H NMR and ^{13}C NMR spectrum for **6**

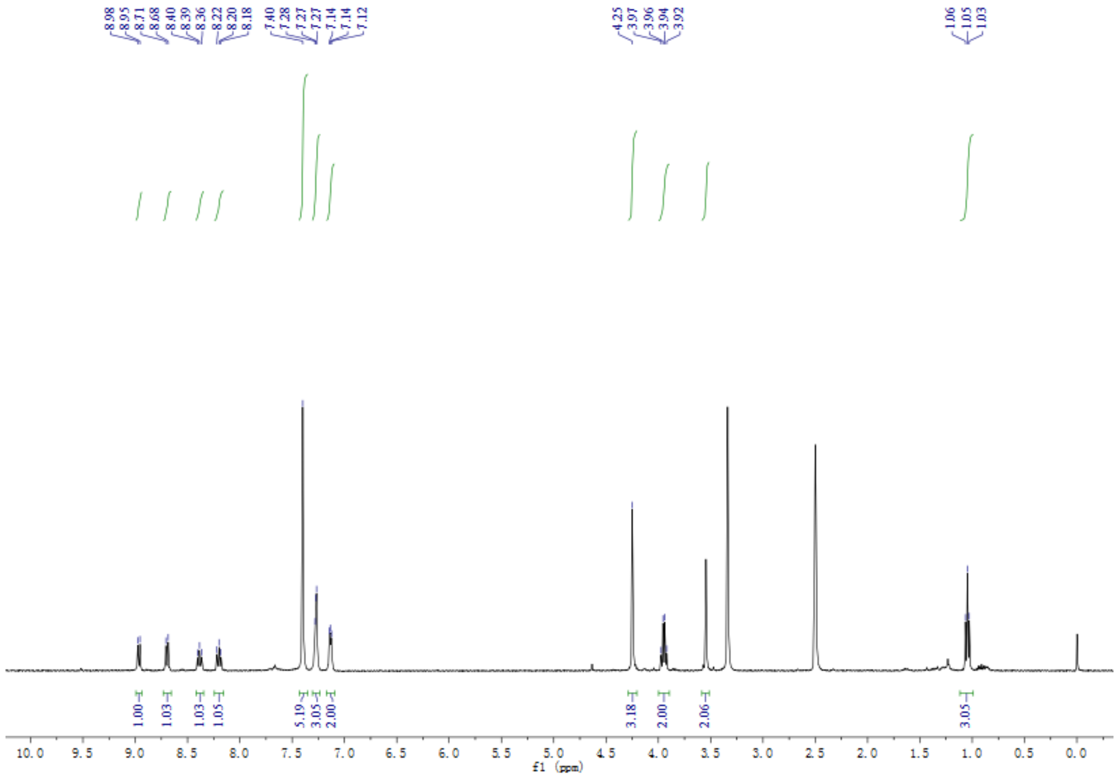

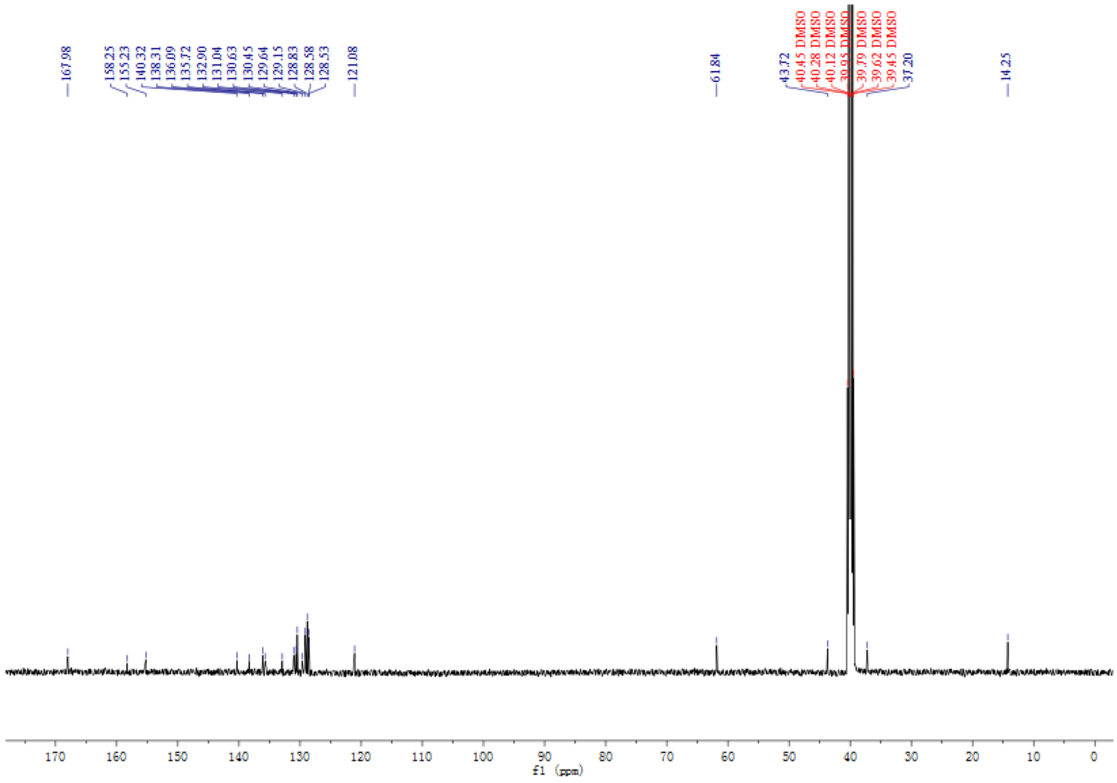

Supplement: Supplementary file 1 [file molecules-25-00268-s001.pdf]
